# A chromosome-scale genome assembly of cucumber (*Cucumis sativus* L.)

**DOI:** 10.1093/gigascience/giz072

**Published:** 2019-06-18

**Authors:** Qing Li, Hongbo Li, Wu Huang, Yuanchao Xu, Qian Zhou, Shenhao Wang, Jue Ruan, Sanwen Huang, Zhonghua Zhang

**Affiliations:** 1 Institute of Vegetables and Flowers, Chinese Academy of Agricultural Sciences, No.12, Haidian District, Beijing 100081, China; 2 Agricultural Genomics Institute at Shenzhen, Chinese Academy of Agricultural Sciences, No. 7, Pengfei Road, Dapeng District, Shenzhen 518124, China; 3 College of Horticulture, Northwest A&F University, Yangling, Shanxi 712100, China

**Keywords:** cucumber, PacBio, Hi-C, genomics, chromosome-scale assembly

## Abstract

**Background:**

Accurate and complete reference genome assemblies are fundamental for biological research. Cucumber is an important vegetable crop and model system for sex determination and vascular biology. Low-coverage Sanger sequences and high-coverage short Illumina sequences have been used to assemble draft cucumber genomes, but the incompleteness and low quality of these genomes limit their use in comparative genomics and genetic research. A high-quality and complete cucumber genome assembly is therefore essential.

**Findings:**

We assembled single-molecule real-time (SMRT) long reads to generate an improved cucumber reference genome. This version contains 174 contigs with a total length of 226.2 Mb and an N50 of 8.9 Mb, and provides 29.0 Mb more sequence data than previous versions. Using 10X Genomics and high-throughput chromosome conformation capture (Hi-C) data, 89 contigs (∼211.0 Mb) were directly linked into 7 pseudo-chromosome sequences. The newly assembled regions show much higher guanine-cytosine or adenine-thymine content than found previously, which is likely to have been inaccessible to Illumina sequencing. The new assembly contains 1,374 full-length long terminal retrotransposons and 1,078 novel genes including 239 tandemly duplicated genes. For example, we found 4 tandemly duplicated tyrosylprotein sulfotransferases, in contrast to the single copy of the gene found previously and in most other plants.

**Conclusion:**

This high-quality genome presents novel features of the cucumber genome and will serve as a valuable resource for genetic research in cucumber and plant comparative genomics.

## Background

Accurate and complete reference genome assembly is essential for genetic and genome-wide studies of individual and multiple species. Cucumber (*Cucumis sativus* L., NCBI:txid3659) is an important vegetable crop and a model plant for sex determination and vascular biology. Four genome assemblies of cucumber, including 1 wild and 3 cultivated accessions, have been released since 2009 [1–6] and were mainly assembled using Illumina short sequences. Compared with the estimated genome size of 350 Mb [4, 5], these assemblies range between 197 and 203 Mb in length; therefore, ∼150 Mb of sequence data are still missing. Cytogenetic and sequence information suggests that ∼100 Mb of satellite sequences, which comprise very large arrays of tandemly repeated DNAs with lengths of 177 or 366 bp, are present in cucumber centromeric/telomeric regions, and these cannot be assembled using current sequencing technology. Current assemblies also have lots of other missing sequences, and this will hamper genetic-based gene isolation, the identification of variations and epigenetic modification sites, and comparative analyses at the population level and across closely related species. The contig and scaffold N50 sizes of the released cucumber genome assembly (version 2.0) are only 30.0 kb and 1.4 Mb, respectively [2], leaving >10,000 gaps. Missing sequences and low contiguity limit the applications of this genome assembly in comparative genomics and genetic research. Therefore, a high-quality and complete cucumber genome assembly is essential.

Repetitive sequences such as transposable elements pose the largest challenge for generating a high-quality genome assembly, especially for plant genomes [7]. The nature of short reads generated by Illumina sequencing technology means that similar repetitive sequences are often collapsed into a single copy. To overcome this limitation, the development of single-molecule real-time (SMRT) sequencing technologies such as Pacific Biosciences (PacBio) and Oxford Nanopore, which generate long reads of >10 kb in size, has advanced in recent years. High-quality genome assemblies for several plants and animals have been generated using these technologies [8–13]. Repetitive sequences in cucumber are estimated to account for 30% of the genome [4], so it is necessary to improve the currently available assembly using long-read sequencing technology.

Scaffolding technologies are critical to accurately order and orient assembled contigs. In past decades, read information from a variety of mate-pair libraries with different insert sizes has been widely used for scaffolding. However, mate-pair library preparation is expensive, and the read information is sometimes also confused by repetitive elements. In recent years, new cost-effective and accurate technologies, including 10X Genomics, optical mapping, and high-throughput chromosome conformation capture (Hi-C), have been developed. These can aid scaffolding by providing long-range contiguity information ranging from ∼50 kb to several megabases [6, 12, 14–16]. These new technologies will greatly benefit the contiguity of the cucumber genome assembly.

## Data Description

Here, we describe the assembly of an improved reference genome assembly for cucumber by combining the read sequence data from PacBio, 10X Genomics, and Hi-C technologies. Comparing the new assembly to the previously released version revealed much improvement in terms of genome completeness and contiguity. This work also presents numerous novel sequences, such as protein-coding genes and intact retrotransposons, and thus provides a robust reference sequence for cucumber genetics.

### Genome sequencing and assembly

The genome of the “Chinese long” inbred line 9930 was assembled several years ago based on Illumina and Sanger sequences [2, 4, 5]. We sequenced this same line using newer technologies; specifically, PacBio, 10X Genomics, and Hi-C. A total of 16.2-Gb PacBio read sequences, representing 46.2-fold genome coverage with a sub-read N50 length of 10.8 kb were generated (Additional File 1). To fully utilize the PacBio data, meta-assembly was performed based on 2 CANU pre-assemblies and 4 FALCON pre-assemblies, resulting in 195 contigs spanning 232.3 Mb in length. Comparing the final assembly with the pre-assemblies showed the complementarity of the 6 initial assemblies (Additional file 2). Assembled contigs containing potential bacteria and plastid contamination were eliminated. Using FinisherSC [17], we aligned raw PacBio reads to the resulting contigs, merged any contigs that could be connected, and the gaps were filled by reads. Illumina sequences were mapped to the assembled sequences to correct any potential sequencing errors (Additional file 3). A total of 49,157 single base pair substitutions and 156,931 small insertion/deletions (indels) were corrected using Pilon [18]. Using 4 genetic maps [3, 19–21], obvious assembly errors were detected, and these contigs were split. All contigs were aligned against the previous assembly (version 2.0), and no obvious errors were observed. Finally, a total of 174 contigs were obtained with a total length of 226.2 Mb and an N50 length of 8.9 Mb (Additional file 4). This represents a ∼234.8-fold improvement in contiguity compared with the previous assembly.

**Figure 1:**
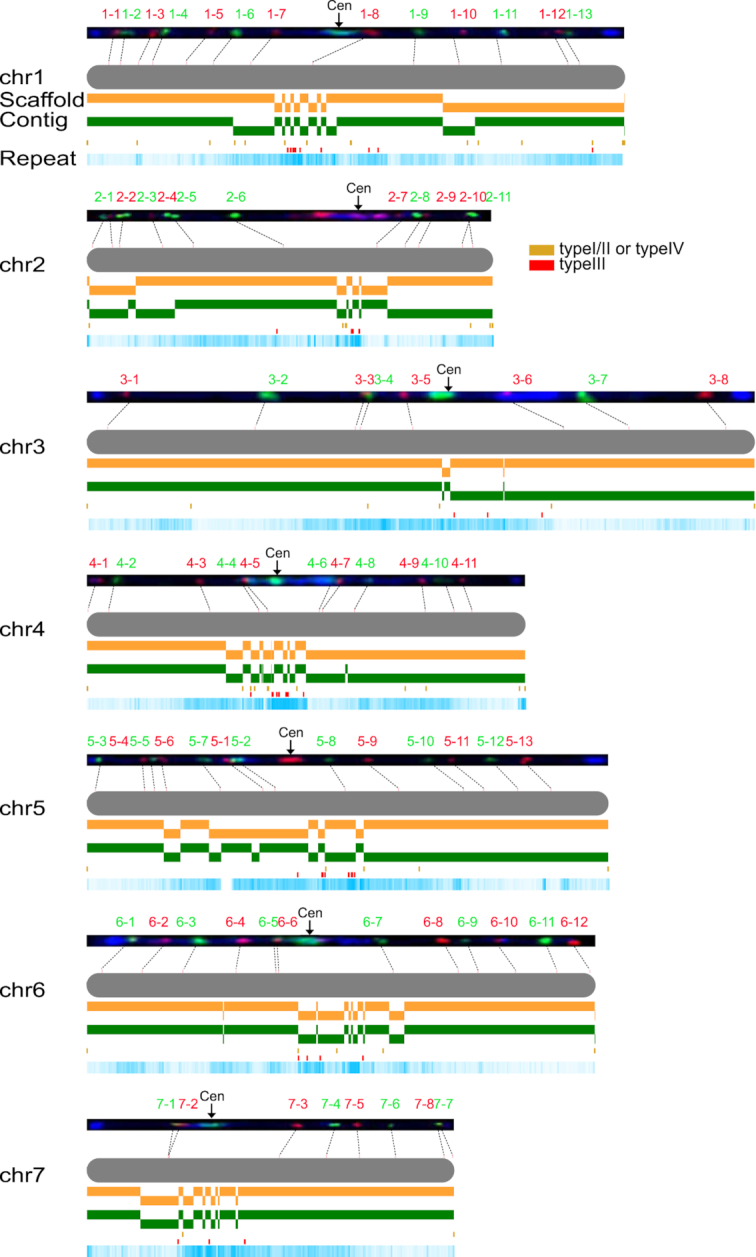
Landscape of the 7 pseudo-chromosome (chr) sequences. All included contigs are shown. Cytogenetic map [22] is integrated with the sequences. Arrows mark positions of the centromeres (Cen). The distribution of satellite and repetitive sequences along the contigs is illustrated below. Fosmid clones are marked in green and red on the 7 chromosomes, and the imaginary lines connect the physical locations and approximate locations of assembled chromosomes.

To build scaffolds, we generated 20.2-Gb linked reads with long-range information of 50-kb DNA fragments using the 10X Genomics platform, and 68.5-Gb long-range contact reads from Hi-C (Additional file 1). Linked reads connected 174 contigs into 157 scaffolds, resulting in an N50 length of 11.5 Mb. On the basis of these scaffolds, we further linked them into 85 super-scaffolds with an N50 of 31.1 Mb using Hi-C data (Additional file 4). Among these super-scaffolds, 7 with a total length of 211.0 Mb corresponded directly to the 7 cucumber chromosomes, thus providing an additional 19.1 Mb of sequence data for the 7 pseudo-chromosome sequences (Fig. 1) relative to the genome v2.0. Lacking Hi-C contact information means that the remaining 78 super-scaffolds (15.2 Mb) cannot be clustered into any of the 7 chromosomes, suggesting that these could be mainly covered by repetitive sequences. Therefore, we present here more complete pseudo-chromosome sequences for the cucumber reference genome.

### Evaluation of the genome quality

To assess the quality of the new genome assembly (v3.0), we mapped 6.0-Gb new Illumina and previous Sanger reads (Additional file 3) to the final assembled sequences. Only 53,179 substitutions and 30,546 small indels were identified as homozygous variations (index >0.9). Thus, the error rates for single base pair and small indels are estimated to be <0.00024 and 0.00014, respectively, which indicates that v3.0 has high accuracy at the single base-pair level.

**Figure 2:**
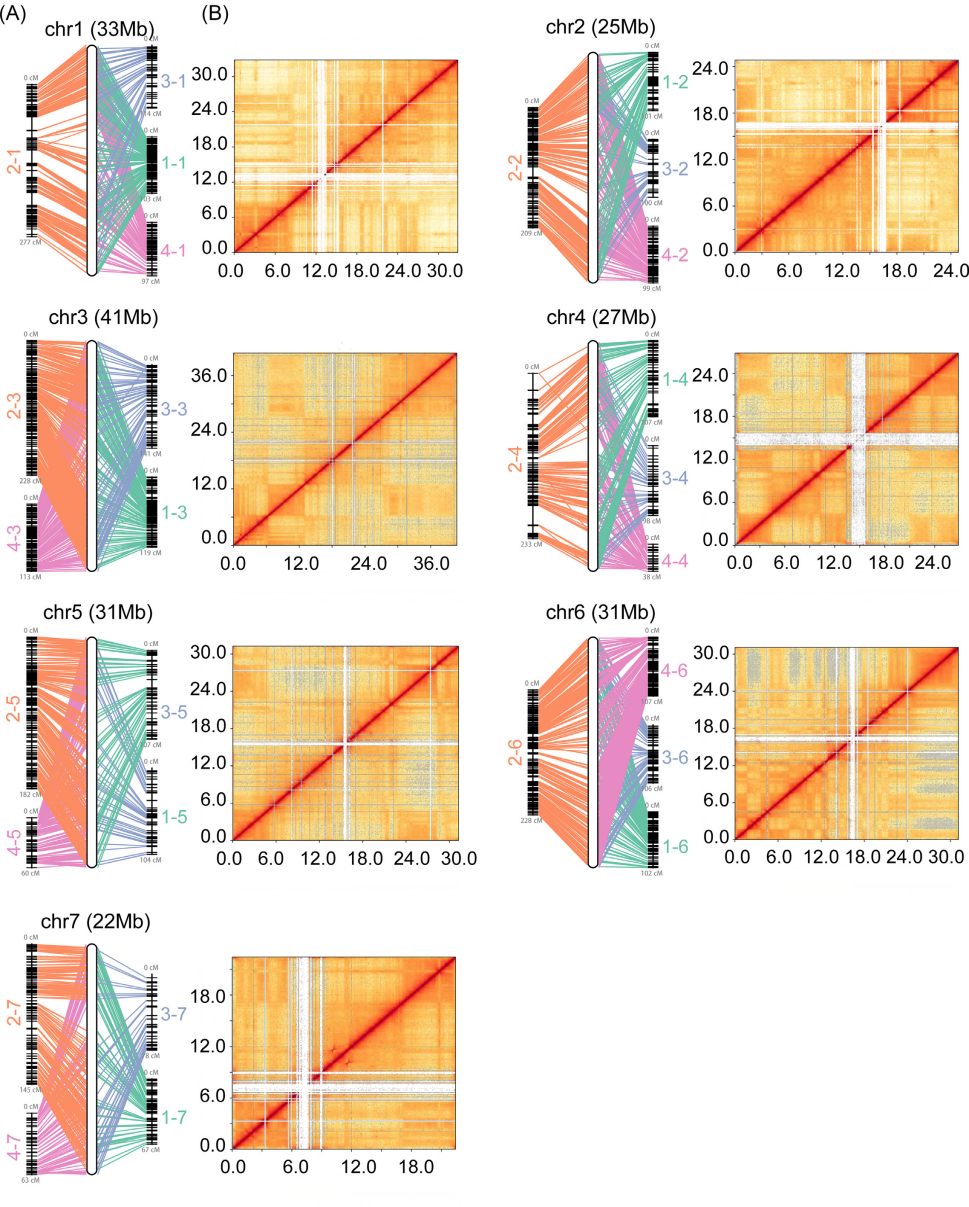
Correlation of genome assembly with genetic maps and Hi-C data. **A**, Integrated genetic and physical maps of the cucumber genome assembly. Super-scaffolds of the genome assembly (middle) were anchored to the 4 linkage groups (left and right): map.1 (green) [3], map.2 (orange) [21], map.3 (light blue) [20], map.4 (pink) [19]. **B**, Heat map of Hi-C contact information. Pixel colors represent different normalized counts of Hi-C links between 30-kb non-overlapping windows for all 7 chromosomes (chr) on a logarithmic scale.

The genome sequences are highly consistent with genetic maps and Hi-C data, which show the high accuracy of contiguity for the assembly (Fig. 2). The orders of genetic markers are consistent with the assembly sequences, with a correlation coefficient of 0.98 on average. From the long-range Hi-C contact information, we can see that most regions show close contacts with nearby sequences, and only the centromeric/telomeric regions have few contacts with other genomic segments.

Integrating the genome assembly with the cytogenetic map [22] reveals the high level of completeness of v3.0 (Fig. 1). Most of the centromeric and telomeric sequences are absent from each of the 7 chromosomes. The main components of the centromere are satellite type III, and these are detected at the ends of the super-scaffolds around the centromeres, indicating their boundaries. Among the 14 ends of the 7 chromosomes, 13 have satellite type I/II/IV components; this constitutes the majority of the telomere, indicating the telomeric boundary. This assembly comprises almost all the genome sequences except for the centromeric and telemetric regions, which are largely made up of satellite sequences and account for ∼30% (∼105 Mb) of total nuclear DNA [23, 24] and thus cannot be assembled using current sequencing technologies [25].

We also explored the consistency between v2.0 and v3.0 genomes using whole-genome alignment (Additional file 5). Many novel sequences appear to be inserted into genome v3.0. The distal sequences on chromosome 5 of v2.0 are translocated to the correct position in v3.0, which is consistent with a previous report [21, 22]. In addition, 2 inversions on chromosomes 4 and 6, which constitute assembly errors in v2.0, were corrected in v3.0. This is supported by the data in the Hi-C heat map (Fig. 2).

To assess the completeness of gene space, we downloaded 121.7 Gb of RNA sequencing (RNA-seq) sequences generated from 39 samples (Additional file 6), including a variety of tissues such as root, stem, leaf, flower, and fruit, and mapped them to assemblies v2.0 and v3.0, respectively. Compared with v2.0, 3.2 Gb additional RNA-seq sequences were mapped in v3.0, resulting in 932.2 kb additional expressed genomic regions. This new assembly therefore represents a higher completeness in terms of gene space.

### Genome annotation reveals novel repetitive sequences and genes

**Figure 3:**
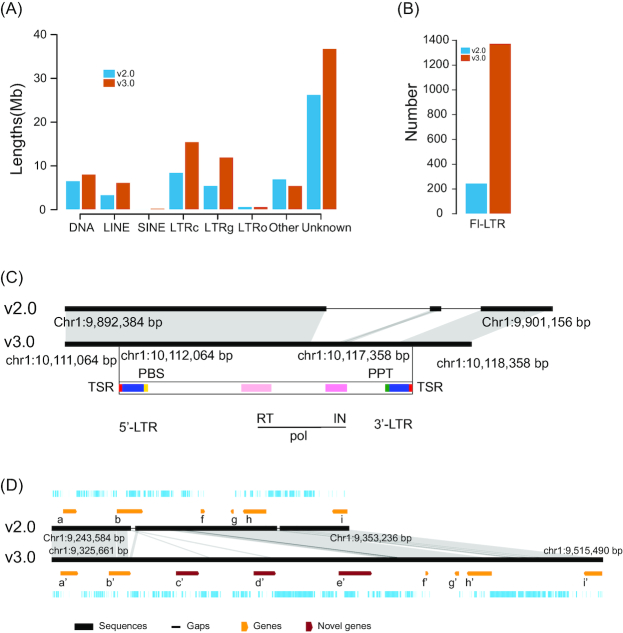
Novel repetitive sequences and genes in assembly v3.0. **A**, Sizes of various types of repetitive sequences in the v2.0 and v3.0 assemblies. DNA, DNA transposons; LINE, Long interspersed nuclear elements; SINE, Short interspersed nuclear elements; LTRc, Copia long terminal repeat retrotransposons; LTRg, Gypsy long terminal repeat retrotransposons; LTRo, Other LTR categories; Unknown, unknown type. **B**, The number of full-length long terminal retrotransposons (FL-LTRs) in v2.0 and v3.0. **C**, A newly predicted FL-LTR in v3.0. TSR, Target site repeat; PBS, Primer bingding site; PPT, Primer polypurine tract; IN, Intergrase; RT, Reverse transcriptase. **D**, An example showing the newly assembled multiple tyrosylprotein sulfotransferase (*TPST*) genes in v3.0. b'-e' are all *TPST* genes, corresponding to CsaV3_1G013960, CsaV3_1G013970, CsaV3_1G013980 and CsaV3_1G013990, respectively.

In v3.0, we identified 82.0 Mb of repetitive sequences, representing 36.43% of the genome (Additional file 7). This is ∼27.6 Mb more than was predicted in v2.0 (54.4 Mb). Among these repetitive sequences, long terminal retrotransposons (LTRs) are the most abundant, and their sizes were markedly increased in v3.0 (Fig. 3A). A total of 1,374 full-length LTRs (FL-LTRs) were predicted in v3.0, 5 times more (267) than in v2.0 (Fig. 3B). Most of these FL-LTRs were partially assembled in v2.0; thus, they were not annotated as FL-LTRs. For example, an FL-LTR on chromosome 1 was not predicted because of the absence of pol-domain and long terminal repeats in v2.0 (Fig. 3C). Insert time analysis of these FL-LTRs reveals that most of them occurred recently in cucumber, explaining the complexity of these regions during the assembly process (Additional file 8) [7].

In v3.0, 24,317 protein-coding genes were predicted by combining 3 methods: *ab initio*, protein homology-based, and transcriptome sequences, using the EVidenceModeler pipeline [26]. Compared with the predicted genes in v2.0, 1,078 genes (Additional file 9) were newly assembled in v3.0, and 2,693 were newly predicted in v3.0 but were not predicted in v2.0 because of sequencing gaps, errors, or annotation pipeline bias. Of the newly assembled genes, 931 are expressed in ≥1 of the above 39 samples with RNA-seq data, indicating their high reliability. Compared with all genes, these genes are characterized by short average length and lower average exon number (Additional file 10). Based on the alignments of genes in v3.0 and v2.0, we also identified 1,970 fragmented genes in v2.0 that correspond to 932 genes in v3.0. Conversely, 687 genes in v2.0 were split into 337 in v3.0 (Additional file 11). Along with the pseudo-chromosomes, the distribution of 1,078 novel genes in v3.0 indicates that 239 are tandemly duplicated genes. For example, in v2.0, only 1 tyrosylprotein sulfotransferase (TPST) was predicted. In most plants, including *Arabidopsis* and tomato, this is a single-copy gene; however, in cucumber v3.0, 4 tandemly duplicated genes were obtained (Fig. 3D). Two predicted TPSTs in the wild cucumber genome [6] also support the presence of multiple TPSTs in the cucumber genome [4]. Therefore, the new genome provides a more complete gene set for functional genomic research in cucumber.

### Features of novel sequences in assembly v3.0

**Figure 4:**
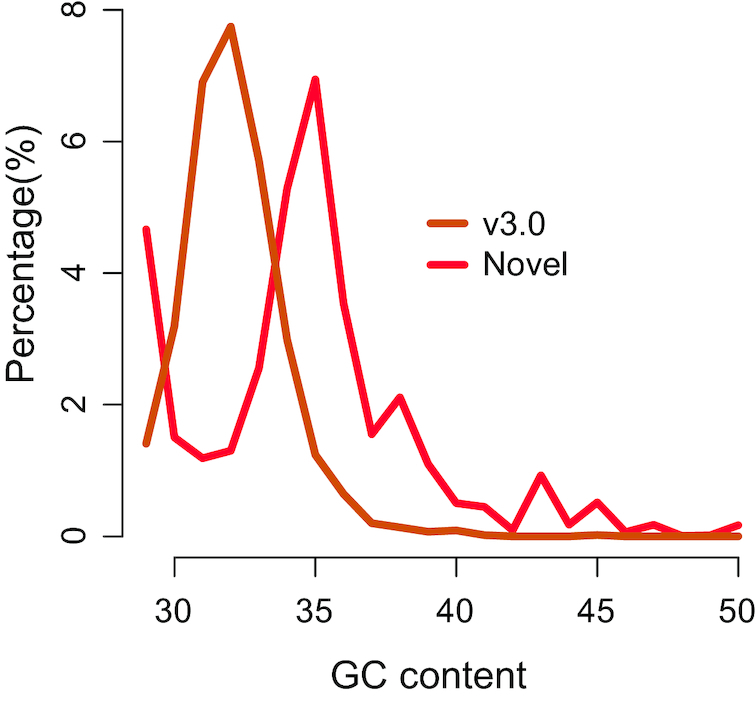
Distribution of GC content for the whole genome and novel sequences in v3.0.

To explore the features of the novel sequences in the new assembly, we analyzed them using Illumina reads and the newly assembled genes. At the whole-genome level, sequences with a guanine-cytosine (GC) content of ∼32.8% were dominantly abundant; however, the distribution of GC content among novel sequences peaked at ∼35.0% (Fig. 4). The newly assembled genes also show a similar GC distribution (Additional file 12). This suggests that sequences with abnormal GC content could only be generated using the PacBio sequencing technology. Among the new genes, >30 domains, including pectinesterase inhibitor (IPR034086, pectinesterase inhibitor, plant; IPR006501, pectinesterase inhibitor domain, etc.), zinc finger, and CCHC-type domain (IPR036875) were significantly enriched (*P* < 0.005) (Additional file 13). These results indicate that the PacBio sequencing technology is advantageous for some types of genes.

## Conclusion

By combining long-read sequences generated by PacBio, long-range information generated by 10X Genomics, and long-range Hi-C contact reads, we provide a high-quality cucumber reference genome for the community. Many repetitive sequences and genes have been identified and added to the assembly, especially sequences with high GC or high adenine-thymine (AT) content, and genes with certain domains. More tandemly duplicated genes were assembled in the new genome. These data provide a valuable resource for comparative genomics, epigenetics, gene isolation, and transposon research.

## Methods

### PacBio sequencing

High-quality genomic DNA was extracted from young leaves of “Chinese long” inbred line 9930 cucumber, using a modified cetyl-trimethylammonium bromide (CTAB) method [27]. Genomic DNA was sheared to a size range of 15–40 kb using a Megaruptor (Diagenode) device (Belgium), and was then used for single-molecule real time (SMRT) library preparation as recommended by PacBio. Two SMRTbellTM templates were prepared in 2014 and 2016, respectively. The first library was sequenced on a PacBio RSII platform, generating 1,470,953 reads (11.0 Gb). The second library was sequenced on a PacBio Sequel platform, generating 628,153 reads (5.2 Gb).

### 10X Genomics linked-read sequencing

A total of 0.3 ng high-molecular-weight DNA was prepared and loaded onto a Chromium Controller chip with 10X Chromium reagents and gel beads, following the recommended protocols [28]. On average, the loaded DNA molecule was ∼50 kb in length. There are ∼1 million droplets on a Chromium Controller chip. Within each droplet, several DNA molecules were sheared, and the sheared DNA fragments were tagged with the same barcode. Then, all barcoded DNA fragments within these droplets were sequenced on an Illumina Hiseq X Ten sequencer to produce 2 × 150 bp paired-end sequences.

### Hi-C read sequencing

Leaves of cucumber line 9930 were fixed with 1% formaldehyde solution, and chromatin was cross-linked and digested using restriction enzyme HindIII. The 5′ overhangs were filled in with biotinylated nucleotides, and free blunt ends were then ligated. After ligation, crosslinks were reversed and the DNA was purified from protein. Purified DNA was treated to remove biotin that was not internal to ligated fragments. The DNA was then sheared into fragment sizes of ∼350 bp. Two sequencing libraries were prepared as described previously [29]. The libraries were sequenced on an Illumina HiSeq X Ten platform. For each library, a total of 223 million paired-end reads of 150 bp in length were generated, representing 195.5-fold coverage of the total cucumber genome. A detailed quality control report for the Hi-C sequencing was yielded by HiCUP (HiCUP, RRID:SCR_005569) [30].

### *De novo* assembly of PacBio reads

Meta-assembly of PacBio reads from SMRT sequencing was performed as previously described [31]. Briefly, meta-assembled contigs were generated using CANU 1.7 (Canu, RRID:SCR_015880) [32] by combining results from 2 CANU and 4 FALCON/til-r assemblies in which the number of contigs ranged from 589 to 1,094 with a contig N50 length between 2.4 and 3.6 Mb (see Additional file 14 for detailed information). Assembled contigs were aligned against bacterial genomes and cucumber plasmid genomes from GenBank using BLAST [33]. If >70% of a contig showed >95% identity with a bacterial or plasmid genome, it was eliminated. Using the FinisherSC pipeline [17] with default parameters, contigs that could be connected by raw PacBio reads were determined, and gaps were filled by reads. To increase the accuracy of contig sequences, previously generated Illumina and Sanger reads (Additional file 3) were aligned to the contigs. Potential sequence errors in the form of single base pair substitutions and indels were corrected by running Pilon (Pilon, RRID:SCR_014731) [18] 2 times, with the following parameters: –fix all –chunksize 20 000 000 –mindepth 0.4 –K 65 –gapmargin 150 000 –vcf –changes –tracks –minmq 10. Corrected contigs were also aligned against the previous genome assembly (v2.0) using MUMmer [34] with default parameters and were anchored onto the 7 linkage groups of the 4 genetic maps [3, 19–21] using ALLMAPS [35]. Conflicting contigs with the orders of molecular markers from the 4 genetic maps were manually checked and split using the alignment results against v2.0. Hi-C data were also aligned to the contigs to check and correct misassemblies.

### Scaffold construction

The final contigs were connected into scaffolds using 10X linked reads by ARKS [36] with the following parameters: m = 20–20000 threads = 20 a = 0.9. By aligning the sequences of genetic markers and Hi-C data to the assembled scaffolds, scaffolds conflicting with the orders of molecular markers or long-range contact information were split. Then, the chromosome-level super-scaffolds were constructed on the basis of the genome-wide chromatin interaction information using the 3d-dna pipeline [14] with the following parameters: -m haploid -i 15000 -r 0. This resulted in 7 chromosome-level super-scaffolds, representing 7 cucumber pseudo-chromosomes, and 78 short-length super-scaffolds that could not be clustered because they lacked interactions with the 7 chromosome-level super-scaffolds.

### Pseudo-chromosome construction

The 7 chromosome-level super-scaffolds were anchored onto the 7 linkage groups of the 4 genetic maps [3, 19–21] and oriented into the 7 pseudo-chromosomes using ALLMAPS [35] with default parameters. The pseudo-chromosomes were further integrated with the cytogenetic map by mapping the marker sequences and satellite sequences (Type I/II/III/IV) onto the assembly using BLASTN (BLASTN, RRID:SCR_001598, v2.2.15) at an e-value cutoff of 0.05. Satellite sequences are abundantly distributed within centromeric and telomeric regions, so the positions of centromeres and telomeres were marked accordingly.

### Genome annotation

#### Repetitive sequences

RepeatModeler (RepeatModeler, RRID:SCR_015027 [37]) was used to search *de novo* for repetitive sequences within genome assemblies v3.0 and v2.0. Identified repeats and the TIGR plant repeat database [38] were then used to identify and mask the repeats in v3.0 and v2.0 using RepeatMasker (RepeatMasker, RRID:SCR_012954 [39]). The repeats were classified into different types based on RepeatMasker annotation. FL-LTRs were also identified using LTR_Finder (LTR_Finder, RRID:SCR_015247, v1.0.6) [40], with the command line “ltr_finder genome.fa -s tRNAdb/Athal-tRNAs.fa -a ps_scan > result.txt”. The long terminal repeats of FL-LTRs were aligned with MUSCLE (MUSCLE, RRID:SCR_011812) [41], and the nucleotide distance (*D*) was estimated using the Kimura 2-parameter (K2p) (transition–transversion ratio) criterion, as implemented in the distmat program of EMBOSS (EMBOSS, v6.60, RRID:SCR_008493) [42]. The insertion time (*T*) of an LTR retrotransposon was calculated using the following formula: *T* = *D*/2μ, where μ = 4.5e–9, and the rate of nucleotide substitution (μ) was inferred according to Nystedt's method [43].

#### Protein-coding genes

Putative protein-coding genes were predicted using EVidenceModeler (EVidenceModeler, RRID:SCR_014659) [26] by integrating several *ab initio* gene predictors, including Augustus (Augustus, RRID:SCR_008417) [44], GlimmerHMM (GlimmerHMM, RRID:SCR_002654) [45], and SNAP (SNAP, RRID:SCR_007936) [46], as well as RNA-seq data and homologous proteins from other plant species. A total of 121.7 Gb RNA-seq sequences generated from 39 samples (Additional file 6), including tissues such as root, stem, leaf, flower, and fruit [2, 47, 48], were used for gene prediction. In addition, genes in v2.0 that were not predicted in v3.0 were added into the protein-coding gene set using Spaln [49].

#### Functional annotation of protein-coding genes

All predicted proteins were aligned against proteins found in UniProt [50] and The Arabidopsis Information Resource (TAIR, [51]) databases. Predicted proteins were annotated as the best-matched protein. Functional annotation was also performed using InterProScan (InterProScan, RRID:SCR_005829). Gene Ontology terms were assigned according to InterPro classification.

### Comparative analyses between assemblies v2.0 and v3.0

#### Evaluating the accuracy of the genome

The accuracy of genome assembly quality was assessed by aligning previous Sanger reads and 6.0 Gb of new Illumina reads to the corrected contigs using BWA (BWA, RRID:SCR_010910) [52]. Genomic variations were called using GenomeAnalysisTK [53] with default parameters. Considering that the sequenced cucumber 9930 is a highly inbred line, we expected a very low heterozygous rate: index > 0.9.

#### Whole-genome alignment

Whole-genome alignment of v3.0 and v2.0 genomes was conducted (Additional file 5) using the nucmer program within MUMmer software (version 4.0.0beta2) [34], with parameters “-l 100 -c 100”. Then, show-coords was used to show and filter nucmer results with the parameters “o -l -r -I 99 -L 1000”. The figure (Additional file 5) was plotted using the Python package svgwrite [54].

#### Mapping RNA-seq data

All downloaded RNA-seq reads were mapped to genome assemblies v2.0 and v3.0 using TopHat 2.1.1 (TopHat, RRID:SCR_013035) with default parameters [55]. On the basis of the alignments, transcripts were assembled using Cufflinks 2.2.1 (Cufflinks, RRID:SCR_014597) without genome guidance [56].

#### Identification of novel genes in v3.0

Coding sequences of predicted genes in v3.0 were aligned against those in v2.0 using BLAST and vice versa. Combined with syntenic information, gene pairs were determined on the basis of the alignments. The corresponding genes were classified as one-to-one, one-to-multiple, or multiple-to-multiple using Python scripts. For the remaining genes in v3.0, gene sequences, including introns, were aligned against the v2.0 genome. If the matched region did not meet the threshold of coverage >50% and identity >95%, then the query gene was considered to be novel in v3.0. Otherwise, the sequences of matched regions in v2.0 were extracted and then aligned against the v3.0 genome. If the matched region in v3.0 covered the whole query gene and the identity was >95%, we considered the v3.0 query gene to have an unpredicted counterpart in v2.0. Genes not classified above were also considered to be novel genes.

#### GC content

Genome sequences were split into multiple non-overlapping 100-kb windows. For each window, the GC content was calculated using a Python script. For the novel sequences in v3.0, the GC content of each DNA fragment was independently calculated.

#### InterPro domain enrichment

To identify enriched InterPro domains for the novel genes, the observed number of each domain among novel genes was compared with the expected number among all genes using the χ^2^ test. InterPro domains with *P*-values <0.005 were regarded as being enriched.

#### Identification of tandemly duplicated genes

OrthoMCL [57] was used to identify orthologous groups in v3.0 genes. Genes in the same orthologous group and located next to each other on 1 chromosome were considered to be tandemly duplicated genes.

## Availability of supporting data and materials

The sequence data supporting the results of this article are available in the NCBI SRA with accession No. SRP139269 (PacBio: SRX5437838 and SRX5437837; Hi-C: SRX3918394, SRX3918395; 10X: SRX3918396). Genome sequences and the corresponding annotations, in GFF3 format, are both available from an International Cucurbits Genomics Initiative (ICUGI) FTP server [58]. Supporting data and materials are also available in the *GigaScience* GigaDB database [59].

## Additional files

**Additional file 1**. Summary of sequencing data from PacBio, 10X Genomics, and Hi-C platforms.

**Additional file 2**. Distribution of gaps across the meta-assembly and the 6 initial assemblies. The outer circle relates to the 7 pseudo-chromosomes of cucumber. Circles a–f indicate the contig tracks of the meta-assembly and 6 initial assemblies, of which gaps are colored in white. a, Meta assembly; b, CANU1 assembly; c, CANU2 assembly; d, FALCON1 assembly; e, FALCON2 assembly; f, FALCON3 assembly; g, FALCON4 assembly.

**Additional file 3**. Summary of the previous sequence reads generated from Illumina and Sanger libraries.

**Additional file 4**. Genome assembly statistics. Scaffolds were built using 10X Genomics linked reads based on assembled contigs and were clustered and ordered into super-scaffolds based on Hi-C data.

**Additional file 5**. Whole-genome synteny for genomes of v3.0 and v2.0. Bold lines in orange and bold lines in blue indicate chromosomes of v3.0 and v2.0, respectively, and gold lines indicate alignment between the v2.0 and v3.0 genomes.

**Additional file 6**. SRA IDs of the RNA-seq data used in this study.

**Additional file 7**. Length and percentage of various repetitive sequences in v3.0.

**Additional file 8**. Distribution of the times of LTR insertion events in v2.0 and v3.0.

**Additional file 9**. Annotation of the newly assembled genes in v3.0.

**Additional file 10**. Box-plot for length of genes, coding sequences, and introns in the whole set of genes and newly predicted genes. The average lengths of genes, exons, and introns are labeled in the figure.

**Additional file 11**. Different alignment types of genes between v2.0 and v3.0. a. Based on the alignments of genomes v3.0 and v2.0, 1,970 fragmented genes were found in v2.0, which correspond to 932 genes in V3.0. b. 687 genes in v2.0 are split into 337 in v3.0.

**Additional file 12**. GC content of novel genes and the whole genome.

**Additional file 13**. Enriched InterPro terms for the novel genes in v3.0.

**Additional file 14**. Summary of the software, parameters, and results of the meta-assembly process.

giz072_GIGA-D-18-00507_Original_Submission

giz072_GIGA-D-18-00507_Revision_1

giz072_GIGA-D-18-00507_Revision_2

giz072_Response_to_Reviewer_Comments_Original_Submission

giz072_Response_to_Reviewer_Comments_Revision_1

giz072_Reviewer_1_Report_Original_Submission JosÃ© Blanca -- 1/24/2019 Reviewed

giz072_Reviewer_2_Report_Original_Submission Zhangjun Fei -- 1/29/2019 Reviewed

giz072_Reviewer_3_Report_Original_Submission Isobel Parkin -- 2/7/2019 Reviewed

giz072_Reviewer_3_Report_Revision_1 Isobel Parkin -- 3/21/2019 Reviewed

giz072_Reviewer_4_Report_Original_Submission Massimo Iorizzo -- 2/19/2019 Reviewed

giz072_Reviewer_4_Report_Revision_1 Massimo Iorizzo -- 4/22/2019 Reviewed

giz072_Supplemental_Files

## Abbreviations

AT: adenine-thymine; BLAST: Basic Local Alignment Search Tool; bp: base pairs; BWA: Burrows-Wheeler Aligner; chr: chromosome; CTAB; cetyl trimethylammonium bromide; EMBOSS: European Molecular Biology Open Software Suite; FL-LTR: full-length long terminal retrotransposon; FTP: file transfer protocol; Gb: gigabase pairs; GC: guanine-cytosine; Hi-C: high-throughput chromosome conformation capture; ICUGI: International Cucurbits Genomics Initiative; indel: insertion/deletion (of bases); kb: kilobase pairs; LINE, long interspersed nuclear elements; LTR: long terminal retrotransposon; Mb: megabase pairs; NCBI: National Center for Biotechnology Information; PacBio: Pacific Biosciences; PBS, primer binding site; PPT, primer polypurine tract; RNA-seq: RNA sequencing; SINE, short interspersed nuclear elements; SMRT: single-molecule real-time; SRA: Sequence Read Archive; TAIR: The Arabidopsis Information Resource; TPST: tyrosylprotein sulfotransferase; TSR, target site repeat.

## Competing interests

The authors declare that they have no competing interests.

## Funding

This work was supported by the China National Key Research and Development Program for Crop Breeding (grant No. 2016YFD0100307 to Z.Z.), the National Science Fund for Excellent Young Scholars (grant No. 31322047 to Z.Z.), the National Natural Science Foundation of China (grant number 31772304 to Z.Z.), and the National Youth Top-notch Talent Support Program in China (Z.Z.). This work was also supported by the Science and Technology Innovation Program of Chinese Academy of Agricultural Science (CAAS-ASTIP-IVFCAAS).

## Authors’ contributions

Z.Z. conceived and designed the research. S.W. and W.H. participated in the material preparation. W.H., J.R., and H.L. performed the genome assembly and scaffolding. Q.L., H.L., Q.Z., and Y.X. performed the annotation and comparative analysis. Z.Z. wrote the manuscript. S.H. revised the manuscript. All authors read and approved the final version of the manuscript.
