# Supplementary material for: A chromosome-scale genome assembly of cucumber (Cucumis sativus L.)
Source: Gigascience. 2019 Jun 18;8(6):giz072. doi: 10.1093/gigascience/giz072 (PMC6582320; doi:10.1093/gigascience/giz072)
Supplement: giz072_GIGA-D-18-00507_Original_Submission [file giz072_giga-d-18-00507_original_submission.pdf]

GigaScience

A chromosome-scale genome assembly of cucumber (Cucumis sativus L.)

--Manuscript Draft--

**Manuscript Number:**	GIGA-D-18-00507
**Full Title:**	A chromosome-scale genome assembly of cucumber (Cucumis sativus L.)
**Article Type:**	Data Note
**Funding Information:**	China National Key Research and Development Program for Crop Breeding (2016YFD0100307) — Prof. Zhonghua Zhang<br>National Natural Science Foundation of China (31322047,31772304) — Prof. Zhonghua Zhang<br>National Youth Top-notch Talent Support Program in China (None) — Prof. Zhonghua Zhang<br>Science and Technology Innovation Program of Chinese Academy of Agricultural Science (None) — Prof. Zhonghua Zhang
**Abstract:**	Background<br>An accurate and complete reference genome assembly is fundamental for biological research. Cucumber is an important vegetable crop and model system for sex determination and vascular biology, and its draft genomes have been assembled using low coverage of Sanger sequences and high coverage of Illumina sequences, but the incompleteness and low quality genome limit the applications in comparative genomics and genetic research. Therefore, a high-quality and complete cucumber genome assembly is of great necessity.<br>Findings<br>We assembled single-molecular real-time (SMRT) sequencing reads into a significantly improved cucumber reference genome including 174 contigs with N50 of 8.9 Mb, providing an extra 29.0 Mb of sequences. Using 10X genomics, and high-throughput chromosome conformation capture (Hi-C) data, 89 contigs were directly linked into the seven pseudo-chromosome sequences. The newly assembled regions abundantly show higher GC content, likely inaccessible to Illumina sequencing. The new assembly contains 1,374 full-length long terminal retrotransposons (LTRs) and 1,078 novel genes including 239 tandemly duplicated genes. For example, four tandemly duplicated tyrosylprotein sulfotransferases (TPSTs), which is a single copy gene in most other plants, were newly assembled, this feature may be specific in cucurbits. Moreover, almost double the number of methylated sites were found in the new assembly in comparison to the previous one, providing a more complete genome for epigenetic analysis.<br>Conclusion<br>This high-quality genome represents novel features of cucumber genome, and will serve as a valuable resource for genetic research in cucumber as well as plant comparative genomics.
**Corresponding Author:**	Zhonghua Zhang<br><br>CHINA
**Corresponding Author Secondary Information:**	
**Corresponding Author's Institution:**	
**Corresponding Author's Secondary Institution:**	
**First Author:**	Zhonghua Zhang
**First Author Secondary Information:**	
**Order of Authors:**	Zhonghua Zhang

Powered by Editorial Manager® and ProduXion Manager® from Aries Systems Corporation

	Qing Li
	Hongbo Li
	Wu Huang
	Yuanchao Xu
	Qian Zhou
	Shenhao Wang
	Jue Ruan
	Sanwen Huang
**Order of Authors Secondary Information:**	
**Additional Information:**	
**Question**	**Response**
Are you submitting this manuscript to a special series or article collection?	No
**Experimental design and statistics**<br><br>Full details of the experimental design and statistical methods used should be given in the Methods section, as detailed in our Minimum Standards Reporting Checklist. Information essential to interpreting the data presented should be made available in the figure legends.<br><br>Have you included all the information requested in your manuscript?	Yes
**Resources**<br><br>A description of all resources used, including antibodies, cell lines, animals and software tools, with enough information to allow them to be uniquely identified, should be included in the Methods section. Authors are strongly encouraged to cite Research Resource Identifiers (RRIDs) for antibodies, model organisms and tools, where possible.<br><br>Have you included the information requested as detailed in our Minimum Standards Reporting Checklist?	Yes
**Availability of data and materials**	Yes

Powered by Editorial Manager® and ProduXion Manager® from Aries Systems Corporation

All datasets and code on which the conclusions of the paper rely must be either included in your submission or deposited in publicly available repositories (where available and ethically appropriate), referencing such data using a unique identifier in the references and in the "Availability of Data and Materials" section of your manuscript.<br><br>Have you have met the above requirement as detailed in our Minimum Standards Reporting Checklist?	

Manuscripts

Click here to download Manuscript cucumber (Cucumis sativus L.)_zzh.docx

A chromosome-scale genome assembly of cucumber (*Cucumis sativus* L.)

Qing Li[1,#], Hongbo Li[1,#], Wu Huang[1,2,#], Yuanchao Xu[1], Qian Zhou[1,2], Shenhao Wang[3], Jue Ruan[2], Sanwen Huang[2], Zhonghua Zhang[1,*]

[1] Institute of Vegetables and Flowers, Chinese Academy of Agricultural Sciences, No.12, Haidian District, Beijing 100081, China.

[2] Agricultural Genomics Institute at Shenzhen, Chinese Academy of Agricultural Sciences, No. 7, Pengfei Road, Dapeng District, Shenzhen 518124, China.

[3] College of Horticulture, Northwest A&F University, Yangling, Shanxi 712100, China

[#] These authors contributed equally to this work.

[*] Address correspondence to Zhonghua Zhang (zhangzhonghua@caas.cn)

Author for Contact details:

Institute of Vegetables and Flowers

Chinese Academy of Agricultural Sciences

No.12 Zhongguancun South St., Haidian District Beijing 10081, P.R.China

Tel: +86-10-62117612

Mobile Phone: +8613699205910

Email: zhangzhonghua@caas.cn

Abstract

Background

An accurate and complete reference genome assembly is fundamental for biological research. Cucumber is an important vegetable crop and model system for sex determination and vascular biology, and its draft genomes have been assembled using low coverage of Sanger sequences and high coverage of Illumina sequences, but the incompleteness and low quality genome limit the applications in comparative genomics and genetic research. Therefore, a high-quality and complete cucumber genome assembly is of great necessity.

Findings

We assembled single-molecular real-time (SMRT) sequencing reads into a significantly improved cucumber reference genome including 174 contigs with N50 of 8.9 Mb, providing an extra 29.0 Mb of sequences. Using 10X genomics, and high-throughput chromosome conformation capture (Hi-C) data, 89 contigs were directly linked into the seven pseudo-chromosome sequences. The newly assembled regions abundantly show higher GC content, likely inaccessible to Illumina sequencing. The new assembly contains 1,374 full-length long terminal retrotransposons (LTRs) and 1,078 novel genes including 239 tandemly duplicated genes. For example, four tandemly duplicated tyrosylprotein sulfotransferases (TPSTs), which is a single copy gene in most other plants, were newly assembled, this feature may be specific in cucurbits. Moreover, almost double the number of methylated sites were found in the new assembly in comparison to the previous one, providing a more complete genome for epigenetic analysis.

Conclusion

This high-quality genome represents novel features of cucumber genome, and will serve as a valuable resource for genetic research in cucumber as well as plant comparative genomics.

Keywords

cucumber; PacBio; Hi-C; genomics; chromosome-scale assembly

Data Description

Introduction

An accurate and complete reference genome assembly is essential for genetic and genome-wide studies of both individual species and multiple species. For cucumber (*Cucumis Sativus* L.), which is an important vegetable crop and has been served as model plant for sex determination and vascular biology, four genome assemblies including one wild and three cultivated accessions have been released since 2009 [1-5], they are based largely on assembling Illumina sequences. In comparison to the estimated genome size of 350 Mb [5], these assemblies ranging from 197 to 203 Mb in length still have approximately 150 Mb of missing sequences. According to the cytogenetic and sequence information, about 100 Mb satellite sequences, which consist of very large arrays of tandemly repeated DNAs with the length of 177 or 366 bp, are mainly present in cucumber centromeric/telemeric regions and cannot be assembled using the current sequencing technology. Besides the satellites, there are still lots of missing sequences in the current assemblies, and this will hamper the genetic-based gene isolation, identification of variations and epigenetic modification sites, and comparative analyses on the population level and across closely related species. Moreover, the contig and scaffold N50 sizes of the released cucumber genome assembly (V2.0) are only 30.0 kb and 1.4 Mb, respectively [3], leaving more than 10,000 gaps. The missing sequences and low contiguity limit the applications of the genome assembly in comparative genomics and genetic research. Therefore, a high-quality and complete cucumber genome assembly is of great necessity.

Repetitive sequences such as transposable elements (TEs) pose the largest challenge for a high-quality genome assembly, especially for plant genomes [6]. The nature of short read from the Illumina sequencing technology often collapsed the similar repetitive sequences into a single copy. To overcome this limitation, single-molecular real-time (SMRT) sequencing technologies such as Pacific Biosciences (PacBio) and Oxford Nanopore, which generate long reads of more than 10kb in size, have been advanced significantly in recent years. For several plants and animals, the high-quality genome assemblies have been generated using these technologies [7-12].

In cucumber, the repetitive sequences are estimated to account for 30%, it is also necessary to improve the assembly using the long read sequencing technology.

Scaffolding technologies are critical for ordering and orienting assembly contigs correctly. For the past decades, reads information from a range of mate-pair libraries with different insert sizes have been widely used for scaffolding. However, preparing mate-pair library is expensive, and the reads information are also confused by repetitive elements. In recent years, new cost-effective and accurate technologies including 10X genomics, optical mapping and high-throughput chromosome conformation capture (Hi-C) have been developed, and they can provide long-range contiguity information ranging from ~50 kb to several mega-bases which aid in scaffolding [11, 13-15]. These new technologies will benefit the contiguity of cucumber genome assembly to a large extent.

Here, we assembled a near-finished reference genome assembly for cucumber by combining the read sequences of PacBio, 10X genomics and Hi-C. Comparison of the new assembly to the previously released version revealed a significant improvement in genome completeness and contiguity. This work represents numerous novel sequences such as protein-coding genes and intact retrotransposons, and thus provides a gold standard reference sequences for genetics in cucumber.

Genome sequencing and assembly

We sequenced the ‘Chinese long’ inbred line 9930, the genome of which was assembled several years ago based on Illumina and Sanger sequences [3, 5], using new technologies including PacBio, 10X genomics, and Hi-C. A total of 16.2 Gb PacBio read sequences representing 46.2-fold genome coverage with a sub-read N50 length of 10.8 kb were generated **(Supplemental Table S1)**. To fully utilize the PacBio data, meta-assembly was performed based on two CANU pre-assemblies and four FALCON pre-assemblies, resulting in a total of 195 contigs spanning 232.3 Mb in length. Comparing the final assembly with the pre-assemblies show the complementarity of the six initial assemblies (**Supplemental Figure S1**). The assembled contigs containing potential bacteria and plastid contamination were eliminated and the resulted sequences were upgraded using FinisherSC. To correct

any potential sequencing errors, those previous Illumina sequences **(Supplemental Table S2)** were mapped onto the initial assembly sequences. A total of 49,157 single base pair substitutions and 156,931 small InDels were corrected using Pilon. Using four genetic maps [4, 16-18], the obvious assembly errors were detected, and these contigs were split. All contigs were aligned against the previous assembly, and no obvious errors were observed. Finally, a total of 174 contigs were obtained with N50 length of 8.9 Mb **(Supplemental Table S4)**, an approximately 234.8-fold improvement in contiguity compared with the previous assembly.

To build scaffolds, we generated 20.2 Gb linked reads with long-range information of 50 Kb DNA fragments using 10X Genomics platform and 68.5 Gb long-range contact reads from Hi-C **(Supplemental Table S1)**. Linked reads connected 174 contigs into 157 scaffolds, resulting in a N50 length of 11.5Mb. On the basis of these scaffolds, we further linked them into 85 super-scaffolds with N50 of 31.1 Mb using Hi-C data **(Supplemental Table S4)**. Among them, seven super-scaffolds with a total length of 211.0 Mb correspond directly to the seven chromosomes of cucumber, thus providing additional 19.1 Mb sequences for the seven pseudo-chromosome sequences (**Figure 1**). Because of lacking contact information, the remaining 78 super-scaffolds cannot be clustered into any of the seven chromosomes, suggesting that these are mainly covered by repetitive sequences. Therefore, we presented here a more complete pseudo-chromosome sequences for cucumber reference genome.

Evaluation of genome quality

To assess the accuracy of the new genome assembly (V3.0), we mapped 6.0 Gb new Illumina and previous Sanger reads (**Supplemental Table S2**) onto final assembly sequences. Only 53,179 substitutions and 30,546 small InDels were identified as homozygous variations (index >0.9), and thus, the error rates for single base pair and small InDels are estimated to be below 0.00024 and 0.00014, respectively, which indicates a high accuracy of V3.0 at the single base-pair level.

The genome sequences are highly consistent with the genetic maps and Hi-C data, which show the high accuracy of contiguity for the assembly (**Figure 2**). The orders of genetic markers are consistent with the assembly sequences with a correlation

coefficient of 0.98 on average. From the long range contact information of Hi-C, we can see that most regions show close contact with nearby sequences, and only the centromeric/telomeric regions have few contacts with other genomic segments.

Integration of the genome assembly with the cytogenetic map [19] revealed the high completeness of V3.0 (**Figure 1**). For all the seven chromosomes, most of the centromeric and telemetric sequences are absent. The main components of the centromere are satellite type III, and they are detected at the ends of the super-scaffolds around the centromeres, indicating the boundaries of them. Among the 14 ends of the seven chromosomes, 13 have satellite type I/II/IV, which constitute the majority of the telomere, indicating the boundary of the telomere. These imply that this assembly consists of almost all the genome sequences except for the centromeric and telemetric regions, which are largely constituted by the satellite sequences and cannot be assembled using current sequencing technologies.

To assess the completeness of gene space, we downloaded 121.7 Gb of RNA-seq sequences generated from 39 samples (**Supplemental Table S5,** SRA ID), including a variety of tissues such as root, stem, leaf, flower, and fruit, and mapped them onto assemblies V2.0 and V3.0. Compared to V2.0, 3.2 Gb additional RNA-seq sequences were mapped on V3.0, resulting in 932.2 Kb additional expressed genomic regions. Therefore, this new assembly represents a higher completeness in gene space.

Genome annotation reveals novel repetitive sequences and genes

In V3.0, we identified a total of 82.0 Mb, representing 36.43% of the genome, as repetitive sequences (**Supplemental Table S6**). In comparison to that (54.4 Mb) of V2.0, approximately 27.6 Mb of extra sequences are predicted as repeats in V3.0. Among the repetitive sequences, the long terminal retrotransposons (LTRs) are the most abundant and the size of them increased markedly in V3.0 (**Figure 3A**). For the LTRs, 1,374 are predicted as full-length LTRs (FL-LTRs) in V3.0, which is five times more than that (267) in V2.0 (**Figure 3B**). Most of these FL-LTRs were partially assembled in V2.0, thus they were not annotated as FL-LTRs. For example, a FL-LTR on chromosome 1 was not predicted because of the absence of pol-domain and long terminal repeats in V2.0 (**Figure 3C**). Dating these FL-LTRs reveals that

most of them recently occurred in cucumber, and this explains the complexity of these regions during the process of assembly (**Supplemental Figure S2**) [6].

A total of 24,317 protein-coding genes in V3.0 were predicted by combining three methods, including *ab initio*, protein homology-based, and transcriptome sequences using the EVM pipeline. In comparison to the predicted genes in V2.0, 1,078 genes (**Supplemental Table S7**) were newly assembled in V3.0, and 2,693 were newly predicted in V3.0 but were not predicted in V2.0 due to sequencing gaps or errors. Among the newly assembled genes, 931 are expressed in at least one of the above 39 samples with RNA-seq data, indicating their high reliability. Distribution analysis of the 1,078 novel genes in V3.0 along the pseudo-chromosomes showed that 239 are tandemly duplicated genes. For example, in V2.0, only one tyrosylprotein sulfotransferase (TPST), which is a single copy gene in most plants such as *Arabidopsis* and tomato, was predicted, but we obtained four in V3.0 (**Figure 3D**). Two predicted TPSTs in the wild cucumber genome also support multiple TPSTs in the cucumber genome. Furthermore, we observed sequencing gaps around the flanking regions of its orthologs in melon and watermelon genomes. These imply that there should be multiple copies of TPSTs in cucurbits, which might be responsible for specific traits in cucurbits. These provide a more complete and contact gene set for functional genomic research in cucumber.

Features of novel sequences in assembly V3.0

To explore the features of the novel sequences in the new assembly, we analyzed the novel sequences that cannot be mapped using Illumina reads as well as the newly assembled genes. The sequences with GC content of approximately 32.8% are dominantly abundant on the whole genome level; however, the GC content distribution of novel sequences shows two peaks at approximately 35.0% and <30.0%, respectively (**Figure 4**). The newly assembled genes also show a similar GC distribution (**Supplemental Figure S3**). These suggest that a number of sequences with abnormal GC content could be only generated using PacBio sequencing technology. Among the new genes, more than 30 domains such as those related to Pectinesterase inhibitor (IPR034086, Pectinesterase inhibitor, plant; IPR006501, Pectinesterase inhibitor domain, etc.) and Zinc finger, the CCHC-type domains

(IPR036875) are significantly enriched (p<0.005) (**Supplemental Table S8**), indicating that the PacBio sequencing technology is advantageous for some types of genes.

More methylation sites detected in assembly V3.0

To assess the value of this assembly for methylation analysis, we compared the methylation sites between the two assemblies using the entire genome bisulfite sequencing data from four samples (**Supplemental Table S9**). Using V3.0 as the reference, the detected methylated sites ranging from 8.9 to 11.2 million were roughly 1.7 times more than that (5.3-7.0 million) using V2.0. For CHG and CHH sites, the numbers using V3.0 were almost double that using V2.0 for each sample (**Figure 5**, **Supplemental Figure S4**). Therefore, a complete genome assembly is critical for methylation analysis.

Conclusion

By combining the long read sequences of PacBio, long range information of 10X genomics and long range contact reads of Hi-C, a high quality cucumber reference genome is provided for the community. A large number of repetitive sequences and genes have been identified and added to the assembly, especially for sequences with high GC or high AT content and genes with certain domains. Among the novel genes, the newly assembled multiple TPSTs may represent the cucurbit-specific feature compared to other plants. These will be a valuable resource for comparative genomics, epigenetics, gene isolation, and transposon research.

Materials and Methods

Genome sequencing.

PacBio sequencing: High quality genomic DNA was extracted from young leaves of ‘Chinese long’ inbred line 9930 using a modified CTAB method [20]. Genomic DNA was sheared to a size range of 15-40 kb by a Megaruptor (Diagenode) device, and then was used for Single-Molecule Real Time (SMRT) library preparation as recommended by Pacific Biosciences. Two SMRTbell^{TM} templates were prepared in 2014 and 2016, respectively. The first library was sequenced on PacBio RSII platform, and 1,470,953 reads (11.0 Gb) were generated. The second library was sequenced on

PacBio Sequel platform, and 628,153 reads (5.2 Gb) were generated. The data set was submitted to NCBI's Small Read Archive (SRP139269).

10X genomics linked-reads sequencing: A total of 0.3 ng high-molecular-weight DNA was prepared and loaded onto Chromium Controller chip with 10X Chromium reagents and gel beads following the recommended protocols (https://support.10xgenomics.com/de-novo-assembly). On average, the loaded DNA molecule is ~50 kb in length. There are about 1 million droplets on a Chromium Controller chip. Within each droplet, several DNA molecules were sheared, and the sheared DNA fragments were tagged with the same barcode. Then all barcoded DNA fragments within these droplets were sequenced on an Illumina Hiseq X Ten sequencer to produce 2 ×150 bp paired-end sequences. The data has been deposited in NCBI's Small Read Archive (SRP139269).

Hi-C reads sequencing: Leaves of cucumber line 9930 were fixed with 1% formaldehyde solution, chromatin was cross-linked and digested using restriction enzyme HindIII. The 5' overhangs were filled in with biotinylated nucleotides, and then free blunt ends were ligated. After ligation, crosslinks were reversed and the DNA purified from protein. Purified DNA was treated to remove biotin that was not internal to ligated fragments. The DNA was then sheared into fragment size of ~350 bp. Two sequencing libraries were prepared as described previously [21]. The libraries were sequenced on an Illumina HiSeq X Ten platform. For each library, a total of 223 millions paired-end reads of 150 bp in length were generated, representing 195.5-fold coverage of cucumber genome in total. A detailed quality control (QC) report for the Hi-C sequencing was yielded by HiCUP [22].

Genome assembly.

De novo assembly of PacBio reads: We performed meta-assembly of the PacBio reads from SMRT sequencing as previously described [23]. In summary, meta-assembled contigs were generated using CANU 1.7 [24] by combining results from two CANU and four FALCON/til-r assemblies in which the number of contigs range from 589 to 1,094 with a contig N50 length between 2.4Mb and 3.6Mb **(see Supplemental Table S3 for detailed information)**. Assembled contigs were aligned against the bacterial genomes and the cucumber plasmid genomes from GenBank

using BLAST [25]. If more than 70% of a contig shows >95% identity with bacterial or plasmid genomes, it was eliminated as sequences from bacterial and plasmid genomes. The resulting contigs were upgraded using FinisherSC [26] with default parameters. To increase the accuracy of the contig sequences, the previously generated Illumina and Sanger reads **(Supplemental Table S2)** were aligned to the contigs. Potential sequence errors in the form of single base pair substitution and insertion/deletion (InDel) were corrected using Pilon [27] with the parameters: --fix all --chunksize 20000000 --mindepth 0.4 --K 65 --gapmargin 150000 --vcf --changes --tracks --minmq 10. In addition, the corrected contigs were aligned against the previous genome assembly (V 2.0) using MUMmer [28] with default parameters and were anchored onto the seven linkage groups of the four genetic maps [4, 16-18] using ALLMAPS [29]. The conflicting contigs with the orders of molecular markers from the four genetic maps were manually checked and split using the alignment results against the previous genome assembly (V2.0). Hi-C data were also aligned to the contigs for checking mis-assemblies.

Scaffold construction: The final contigs were connected into scaffolds using 10X linked reads by ARKS [30] with following parameters: m=20-20000 threads=20 a=0.9. By aligning the sequences of genetic markers and Hi-C data to the assembled scaffolds, we split the scaffolds conflicting with the orders of molecular markers or long-range contact information. Then, the chromosome-level super-scaffolds were constructed on the basis of the genome-wide chromatin interaction information using 3d-dna pipeline [13] with the parameters: -m haploid -i 15000 -r 0, which resulted in seven chromosome-level super-scaffolds, representing seven pseudo-chromosomes of cucumber, and 78 short-length super-scaffolds that cannot be clustered for lacking interactions with the seven chromosome-level super-scaffolds.

Pseudo-chromosome construction: The seven chromosome-level super-scaffolds were anchored onto the seven linkage groups of the four genetic maps [4, 16-18] and orientated into the seven pseudo-chromosomes using ALLMAPS [29] with default parameters. Furthermore, the pseudo-chromosomes were integrated with the cytogenetic map by mapping the marker sequences and satellite sequences (Type I/II/III/IV) onto the assembly using BLASTN (v2.2.15) at the e-value cutoff of 0.05.

The satellite sequences are abundantly distributed within centromeric and telomeric regions so that the positions of centromere and telomere were marked.

Genome annotation.

Repetitive sequences: RepeatModeler (http://www.repeatmasker.org/RepeatModeler/) was used to do *de novo* search for repetitive sequences in the genome assembly V3.0 and V2.0. Identified repeats and the TIGR plant repeats database (http://plantrepeats.plantbiology.msu.edu) were then used to identify and mask the repeats in V3.0 and V2.0 using RepeatMasker (http://www.repeatmasker.org). The repeats were classified into different types based on the annotation of RepeatMasker. Moreover, full length LTR retrotransposons (FL-LTRs) were identified using LTR_Finder(v 1.0.6) [31] with the command line 'ltr_finder genome.fa -s tRNAdb/Athal-tRNAs.fa -a ps_scan > result.txt'. The long terminal repeats of FL-LTRs were aligned with MUSCLE [32] and the nucleotide distance (D) was estimated using the Kimura two-parameter (K2p) (transition-transversion ratio) criterion as implemented in the distmat program of the EMBOSS package (v 6.6.0). The insertion time (T) of an LTR retrotransposon was calculated using the formula: $T= D/2\mu$, where the μ is 4.5e-9, and rate of nucleotide substitution (μ) was inferred according to Nystedt's method [33].

Protein-coding genes: Putative protein-coding genes were predicted using EVidenceModeler [34] by integrating several *ab initio* gene predictors including Augustus (http://augustus.gobics.de), GlimmerHMM [35] and SNAP [36], RNA-seq data and homologous proteins from other plant species. A total of 121.7 Gb RNA-seq sequences generated from 39 samples (**Supplemental Table S5**) including a variety of tissues such as root, stem, leaf, flower and fruit [3, 37, 38] were used for genes prediction. In addition, the genes in V2.0 which are not predicted in V3.0 were added into the protein-coding gene set using Spaln [39].

Functional annotation of protein-coding genes: All predicted proteins were aligned against UniProt (www.uniprot.org) and Arabidopsis proteins (www.arabidopsis.org). The best matched protein is assigned to the predicted proteins. Functional annotation

was also performed using InterProScan. Gene ontology (GO) terms were assigned according to InterPro classification.

Comparative analyses between the assembly V2.0 and V3.0.

Mapping RNA-seq data: All downloaded RNA-seq reads were mapped onto the genome assembly V2.0 and V3.0 using TopHat 2.1.1 with default parameters [40]. On the basis of the alignments, the transcripts were assembled using Cufflinks 2.2.1 without genome guidance [41].

Identification of novel genes in V3.0: The coding sequences of predicted genes in V3.0 were aligned against those in V2.0 using BLAST and *vice versa*. Combining with syntenic information, gene pairs were determined based on the alignments. The corresponding genes are classified into three categories: one to one, one to multiple, multiple to multiple. For the remaining genes in V3.0, the gene sequences including intronic sequences were aligned against V2.0 genome. If the matched region failed the threshold of coverage >50% and identity >95%, the query gene was considered as novel in V3.0. Otherwise, the sequences of matched regions in V2.0 were extracted and then aligned against the V3.0 genome. If the matched region in V3.0 covers the whole query gene and the identity is more than 95%, we considered the query gene in V3.0 had counterpart in V2.0 genome but it was not predicted. The genes which were not classified above are also considered as novel genes.

GC content: The genome sequences were split into multiple non-overlapping 100 kb windows. For each window, the GC content was calculated using python script. For the novel sequences in V3.0, the GC content of each DNA fragment was independently calculated.

InterPro domain enrichment: To identify enriched InterPro domains for the novel genes, the observed number of each domain among novel genes were compared with the expected number among the whole genes using chi-square test. The InterPro domain with p-value < 0.005 is regarded as enriched InterPro domain.

Methylated sites: Genomic DNA was extracted from young leaves of four cucumber accessions including 9930, CG0002, CG6578, CG9160. Bisulfite treatment was applied to determine the cytosine methylation status using the Epitect bisulfite kit (Qiagen). The Bisulfite sequencing was performed on Illumina Hiseq X Ten sequencer. A total of ~66.3 Gb paired-end reads with the length of 150 bp were generated. All reads were mapped onto the genome assembly V2.0 an V3.0, respectively, using BSMAP [42] with following parameters: -p 8 -w 100 -v 0.07 -m 50 -x 300 -s 16. Only those cytosines mapped by at least five unique reads were considered as potential methylated sites. All the above sites were further confirmed by binomial distribution test, and only those with False Discovery Rate (FDR) < 0.01 were retained as methylation sites [43].

Availability of supporting data

The sequence data have been deposited in the NCBI Short Read Archive with accession number SRP139269 (PacBio: SRX3918393; Hi-C: SRX3918394, SRX3918395; 10X: SRX3918396; Methylation: SRX3918397). Novel assembly has been deposited in the NCBI BioProject under accession PRJNA437233. Genome assembly and annotation is also available at www.icugi.org. All supplementary figures and tables are provided in Supplemental.docx and Supplemental.xlsx.

Additional files

Supplemental.docx.

Supplemental TableS7.xlsx.

Supplemental TableS8.xlsx

Abbreviations

TE: transposable element; SMRT: single-molecular real-time; PacBio: Pacific Biosciences; Hi-C: high-throughput chromosome conformation capture; InDels: insertion or deletion of bases; SNP: single-nucleotide polymorphism; RNA-seq: RNA sequencing; SRA: Sequence Read Archive; LTR: long terminal retrotransposons; FL-LTR: full-length LTR; EVM: EVidenceModeler; TPST: tyrosylprotein sulfotransferase; BLAST: Basic Local Alignment Search Tool; NCBI: National Center for Biotechnology Information

Competing interests

All authors report no competing interests.

Acknowledgments

We would like to thank Qingyong Yang for the help of the analysis of Hi-C data.

Author contributions:

Z.Z. conceived and designed the research. S.W. and W.H. participated in the material preparation. W.H., J.R. and H.L. performed the assembly and scaffolding. Q.L., H.L., Q.Z., and Y.X. performed the annotation and comparative analysis. Z.Z. wrote the manuscript. S.H. revised the manuscript.

Funding information:

This work was supported by China National Key Research and Development Program for Crop Breeding (2016YFD0100307 to Z.Z.), National Science Fund for Excellent Young Scholars (31322047 to Z.Z.), National Natural Science Foundation of China (31772304 to Z.Z.), and the National Youth Top-notch Talent Support Program in China (Z.Z.). This work was also supported by the Science and Technology Innovation Program of Chinese Academy of Agricultural Science (CAAS-ASTIP-IVFCAAS).

Figure legends

Figure 1. The landscape of the seven pseudo-chromosome sequences. All included contigs are shown. The cytogenetic map [19] is integrated with the sequences. The arrows mark the positions of the centromeres. The distribution of satellite and repetitive sequences along the contigs is illustrated below.

Figure 2. Genome assembly correlation with genetic maps and Hi-C data. Integrated genetic and physical maps of the cucumber genome assembly. Super-scaffolds of the genome assembly (middle) were anchored to the four linkage groups (left and right): map.1 (green) [4], map.2 (orange) [18], map.3 (light blue) [17], map.4 (pink) [16].

Heat map of Hi-C contact information. The colors of pixels represent different normalized count of Hi-C links between 30 kb non-overlapping windows for all seven chromosomes on a logarithmic scale.

Figure 3. Novel repetitive sequences and genes in assembly V3.0. A. Size of various types of repetitive sequences in the V2.0 and V3.0 assembly. DNA: DNA transposons; LINE: Long Interspersed Nuclear Elements; SINE: Short Interspersed Nuclear Elements; LTRc: Copia Long Terminal Repeat retrotransposons; LTRg: Gypsy Long Terminal Repeat retrotransposons; LTRo: Other LTR categories; Unknown: unknown type. B. The number of full-length LTRs (FL-LTRs) in V2.0 and V3.0. C. A newly predicted full-length LTR in V3.0. D. An example showing the newly assembled multiple tyrosylprotein sulfotransferase (TPST) genes in V3.0.

Figure 4. Distribution of the GC content for the whole genome and novel sequences in V3.0.

Figure 5. The numbers of CHG methylated sites using V2.0 and V3.0 as the reference genome.

Reference

1. Qi J, Liu X, Shen D, Miao H, Xie B, Li X, et al. A genomic variation map provides insights into the genetic basis of cucumber domestication and diversity. Nat Genet. 2013;45:1510-5.
2. Woycicki R, Witkowicz J, Gawronski P, Dabrowska J, Lomsadze A, Pawelkowicz M, et al. The genome sequence of the North-European cucumber (*Cucumis sativus* L.) unravels evolutionary adaptation mechanisms in plants. PLoS one. 2011;6:e22728.
3. Li Z, Zhang Z, Yan P, Huang S, Fei Z and Lin K. RNA-Seq improves annotation of protein-coding genes in the cucumber genome. BMC Genomics. 2011;12:540.
4. Yang L, Koo DH, Li Y, Zhang X, Luan F, Havey MJ, et al. Chromosome rearrangements during domestication of cucumber as revealed by high-density genetic mapping and draft genome assembly. Plant J. 2012;71:895-906.
5. Huang S, Li R, Zhang Z, Li L, Gu X, Fan W, et al. The genome of the cucumber, *Cucumis sativus* L. Nat Genet. 2009;41:1275-81.
6. Maumus F and Quesneville H. Impact and insights from ancient repetitive elements in plant genomes. Curr Opin Plant Biol. 2016;30:41-6.
7. Bickhart DM, Rosen BD, Koren S, Sayre BL, Hastie AR, Chan S, et al. Single-molecule sequencing and chromatin conformation capture enable de novo reference assembly of the domestic goat genome. Nat Genet. 2017;49:643-50.
8. Daccord N, Celton JM, Linsmith G, Becker C, Choisne N, Schijlen E, et al. High-quality de novo assembly of the apple genome and methylome dynamics of early fruit development. Nat Genet. 2017;49:1099-106.
9. Du H, Yu Y, Ma Y, Gao Q, Cao Y, Chen Z, et al. Sequencing and de novo assembly of a near complete indica rice genome. Nat Commun. 2017;8:15324.
10. Gordon D, Huddleston J, Chaisson MJ, Hill CM, Kronenberg ZN, Munson KM, et al. Long-read sequence assembly of the gorilla genome. Science. 2016;352:aae0344.

11. Jiao WB, Accinelli GG, Hartwig B, Kiefer C, Baker D, Severing E, et al. Improving and correcting the contiguity of long-read genome assemblies of three plant species using optical mapping and chromosome conformation capture data. Genome Res. 2017;27:778-86.

12. Jiao Y, Peluso P, Shi J, Liang T, Stitzer MC, Wang B, et al. Improved maize reference genome with single-molecule technologies. Nature. 2017;546:524-7.

13. Dudchenko O, Batra SS, Omer AD, Nyquist SK, Hoeger M, Durand NC, et al. De novo assembly of the Aedes aegypti genome using Hi-C yields chromosome-length scaffolds. Science. 2017;356:92-5.

14. Yeo S, Coombe L, Chu J, Warren RL and Birol I. ARCS: Scaffolding Genome Drafts with Linked Reads. Bioinformatics. 2017; doi:10.1093/bioinformatics/btx675.

15. Zhang GQ, Liu KW, Li Z, Lohaus R, Hsiao YY, Niu SC, et al. The Apostasia genome and the evolution of orchids. Nature. 2017;549:379-83.

16. Ren Y, Zhang Z, Liu J, Staub JE, Han Y, Cheng Z, et al. An integrated genetic and cytogenetic map of the cucumber genome. PLoS One. 2009;4:e5795.

17. Zhang WW, Pan JS, He HL, Zhang C, Li Z, Zhao JL, et al. Construction of a high density integrated genetic map for cucumber (*Cucumis sativus* L.). Theor Appl Genet. 2012;124:249-59.

18. Zhou Q, Miao H, Li S, Zhang S, Wang Y, Weng Y, et al. A sequencing-based linkage map of cucumber. Molecular plant. 2015;8 6:961-3.

19. Sun J, Zhang Z, Zong X, Huang S, Li Z and Han Y. A high-resolution cucumber cytogenetic map integrated with the genome assembly. BMC Genomics. 2013;14:461.

20. Murray MG and Thompson WF. Rapid isolation of high molecular weight plant DNA. Nucleic Acids Res. 1980;8:4321-5.

21. Belton JM, McCord RP, Gibcus JH, Naumova N, Zhan Y and Dekker J. Hi-C: a comprehensive technique to capture the conformation of

genomes. Methods. 2012;58:268-76.

22. Wingett S, Ewels P, Furlan-Magaril M, Nagano T, Schoenfelder S, Fraser P, et al. HiCUP: pipeline for mapping and processing Hi-C data. F1000Res. 2015;4:1310.

23. Raymond O, Gouzy J, Just J, Badouin H, Verdenaud M, Lemainque A, et al. The Rosa genome provides new insights into the domestication of modern roses. Nat Genet. 2018;50:772-7.

24. Koren S, Walenz BP, Berlin K, Miller JR, Bergman NH and Phillippy AM. Canu: scalable and accurate long-read assembly via adaptive k-mer weighting and repeat separation. Genome Res. 2017;27:722-36.

25. Altschul SF, Gish W, Miller W, Myers EW and Lipman DJ. Basic local alignment search tool. J Mol Biol. 1990;215:403-10.

26. Lam KK, LaButti K, Khalak A and Tse D. FinisherSC: a repeat-aware tool for upgrading de novo assembly using long reads. Bioinformatics. 2015;31:3207-9.

27. Walker BJ, Abeel T, Shea T, Priest M, Abouelliel A, Sakthikumar S, et al. Pilon: an integrated tool for comprehensive microbial variant detection and genome assembly improvement. PLoS One. 2014;9:e112963.

28. Kurtz S, Phillippy A, Delcher AL, Smoot M, Shumway M, Antonescu C, et al. Versatile and open software for comparing large genomes. Genome Biol. 2004;5:R12.

29. Tang H, Zhang X, Miao C, Zhang J, Ming R, Schnable JC, et al. ALLMAPS: robust scaffold ordering based on multiple maps. Genome Biol. 2015;16:3

30. Coombe L, Zhang J, Vandervalk BP, Chu J, Jackman SD, Birol I, et al. ARKS: chromosome-scale scaffolding of human genome drafts with linked read kmers. BMC Bioinformatics. 2018;19:234.

31. Xu Z and Wang H. LTR_FINDER: an efficient tool for the prediction of full-length LTR retrotransposons. Nucleic Acids Res. 2007;35:W265-8.

32. Edgar RC. MUSCLE: a multiple sequence alignment method with reduced time and space complexity. BMC Bioinformatics. 2004;5:113.

33. Nystedt B, Street NR, Wetterbom A, Zuccolo A, Lin YC, Scofield DG, et al. The Norway spruce genome sequence and conifer genome

evolution. Nature. 2013;497:579-84.

34. Haas BJ, Salzberg SL, Zhu W, Pertea M, Allen JE, Orvis J, et al. Automated eukaryotic gene structure annotation using EVidenceModeler and the Program to Assemble Spliced Alignments. Genome Biol. 2008;9:R7.

35. Majoros WH, Pertea M and Salzberg SL. TigrScan and GlimmerHMM: two open source *ab initio* eukaryotic gene-finders. Bioinformatics. 2004;20:2878-9.

36. Johnson AD, Handsaker RE, Pulit SL, Nizzari MM, O'Donnell CJ and de Bakker PI. SNAP: a web-based tool for identification and annotation of proxy SNPs using HapMap. Bioinformatics. 2008;24:2938-9.

37. Wei G, Tian P, Zhang F, Qin H, Miao H, Chen Q, et al. Integrative Analyses of Nontargeted Volatile Profiling and Transcriptome Data Provide Molecular Insight into VOC Diversity in Cucumber Plants (*Cucumis sativus*). Plant Physiol. 2016;172:603-18.

38. Qiu L, Jiang B, Fang J, Shen Y, Fang Z, Rm SK, et al. Analysis of transcriptome in hickory (*Carya cathayensis*), and uncover the dynamics in the hormonal signaling pathway during graft process. BMC Genomics. 2016;17:935.

39. Iwata H and Gotoh O. Benchmarking spliced alignment programs including Spaln2, an extended version of Spaln that incorporates additional species-specific features. Nucleic Acids Res. 2012;40:e161.

40. Trapnell C, Pachter L and Salzberg SL. TopHat: discovering splice junctions with RNA-Seq. Bioinformatics. 2009;25:1105-11.

41. Trapnell C, Williams BA, Pertea G, Mortazavi A, Kwan G, van Baren MJ, et al. Transcript assembly and quantification by RNA-Seq reveals unannotated transcripts and isoform switching during cell differentiation. Nat Biotechnol. 2010;28:511-5.

42. Xi Y and Li W. BSMAP: whole genome bisulfite sequence MAPping program. BMC Bioinformatics. 2009;10:232.

43. Schultz MD, Schmitz RJ and Ecker JR. 'Leveling' the playing field for analyses of single-base resolution DNA methylomes. Trends Genet. 2012;28:583-5.

Figure1 Click here to download Figure Figure1.pdf

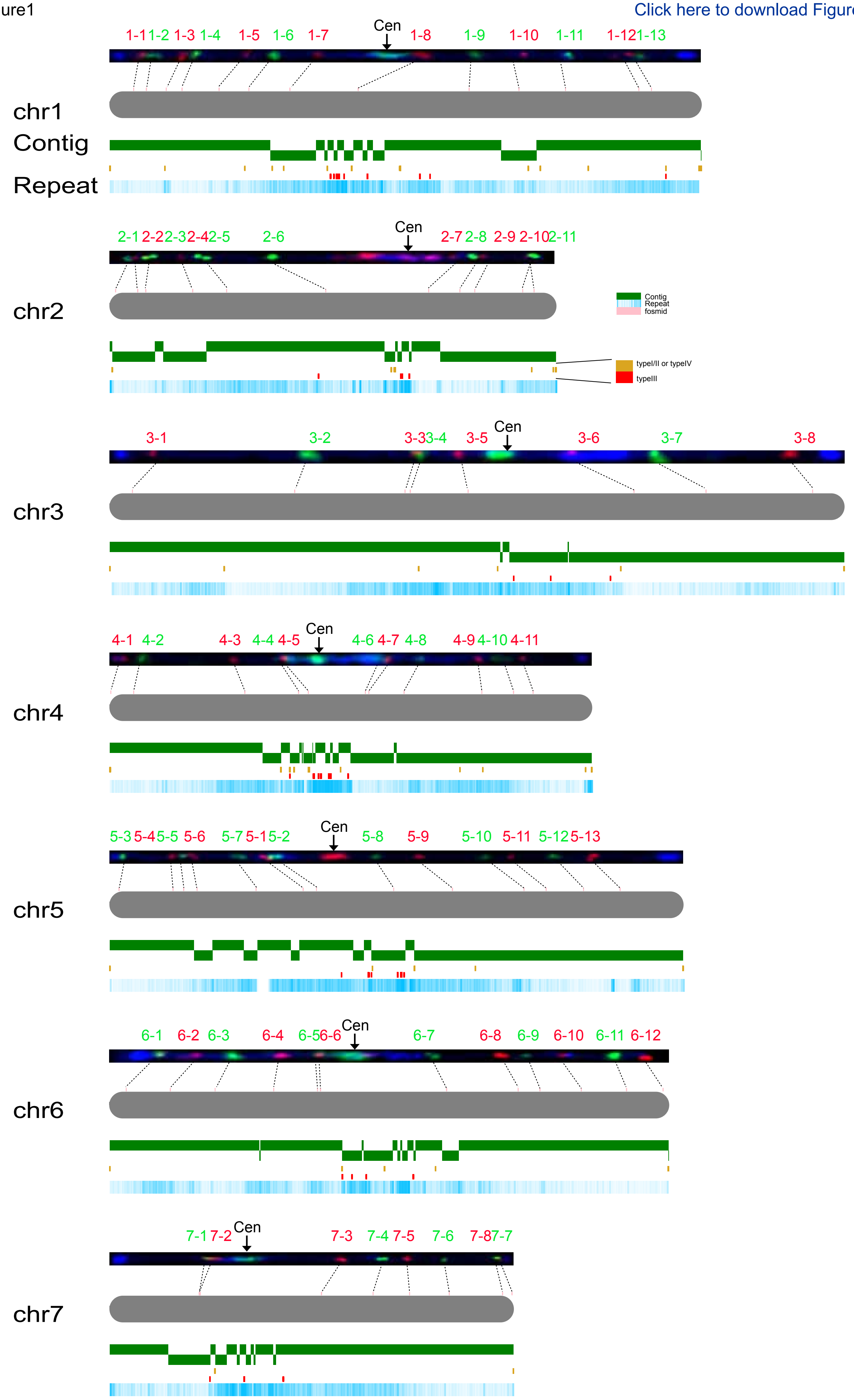

Figure2

Click here to download Figure Figure2.pdf

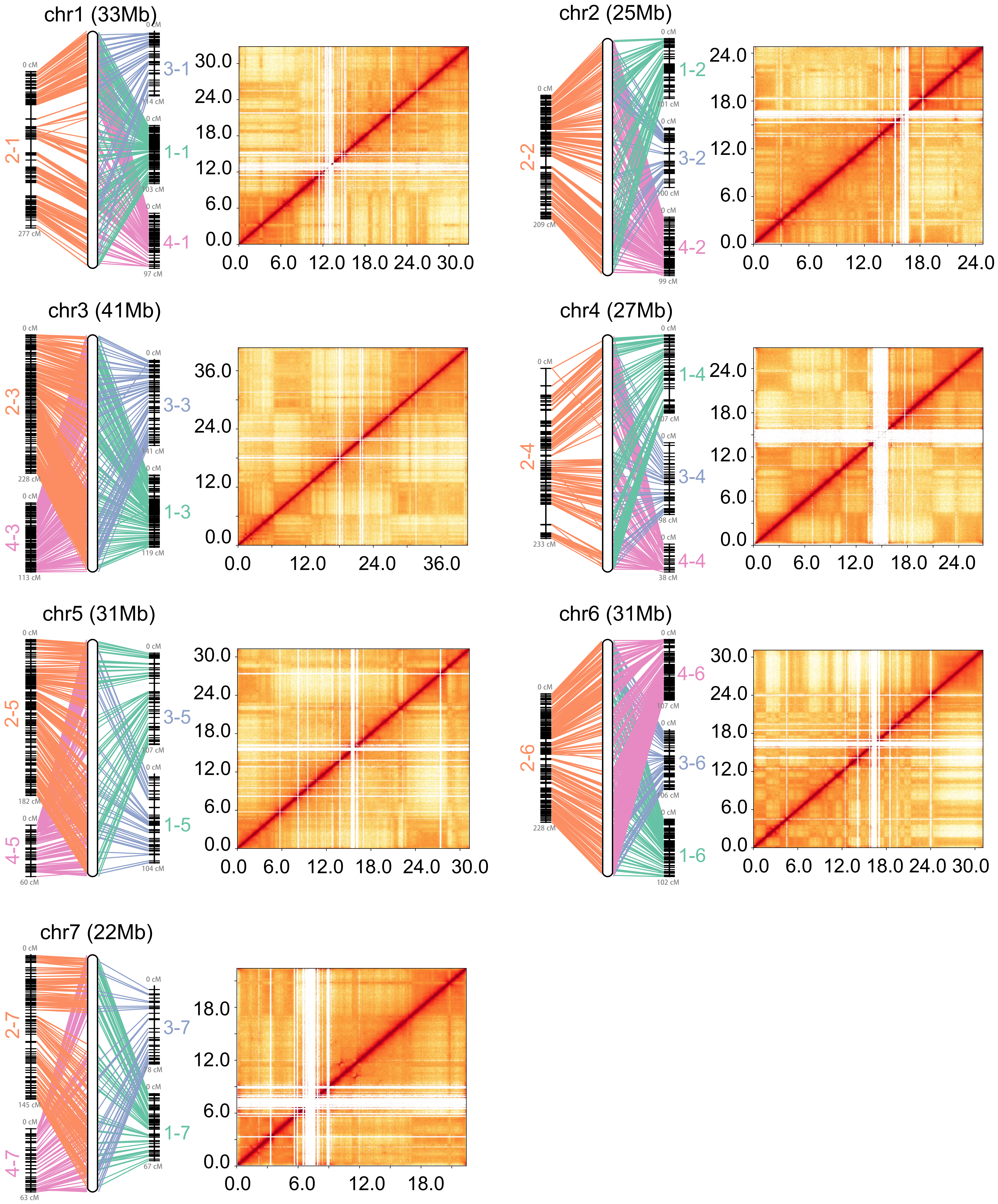

Figure3

Click here to download Figure Figure3.pdf

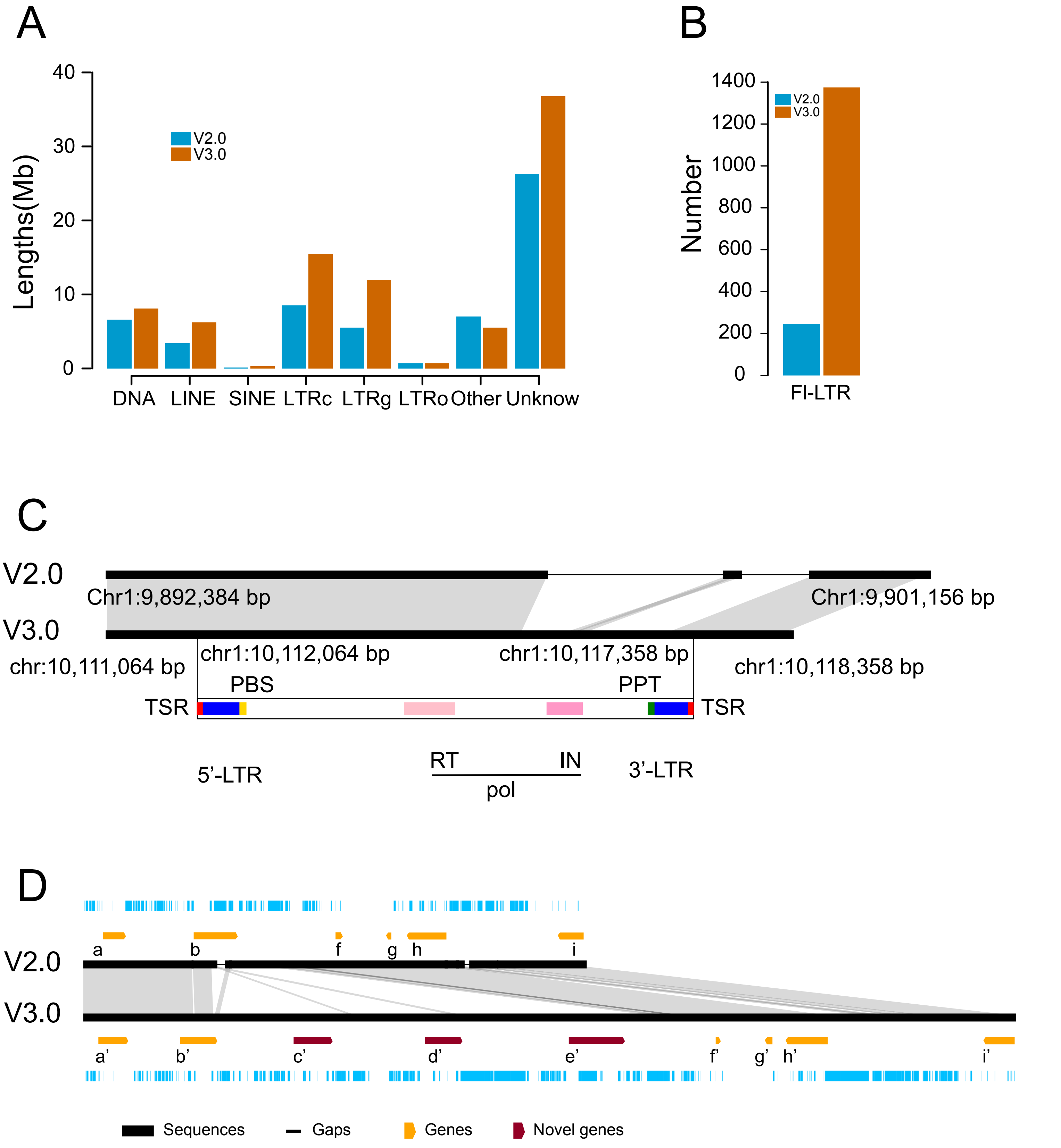

Figure4

Click here to download Figure
Figure4.pdf

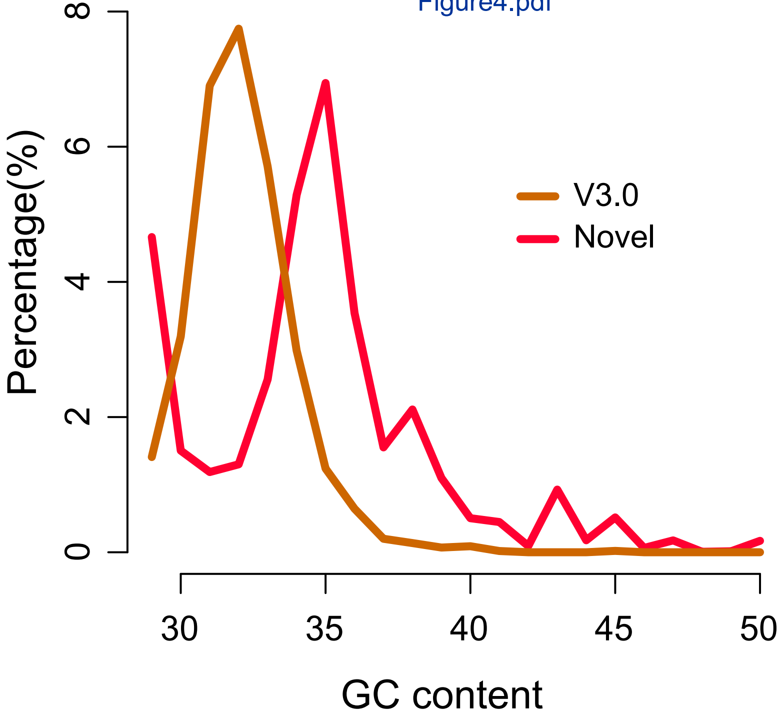

Figure5

Click here to download Figure Figure5.pdf

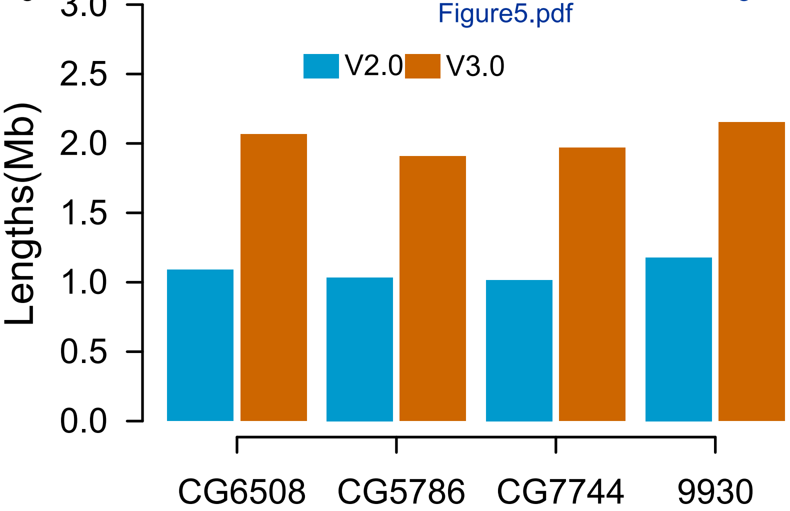

Click here to access/download
Supplementary Material
Supplemental.docx

Click here to access/download
Supplementary Material
Supplemental Table S7.xls

Click here to access/download
Supplementary Material
Supplemental Table S8.xls

 Click here to download Personal Cover Cucumber genome V3.0 CV.docx

Dear Editor:

we would like to submit the manuscript entitled “**A chromosome-scale genome assembly of cucumber (*Cucumis sativus* L.)**” to **GigaScience** for publication as a Data Note.

Cucurbitaceae, or the gourd family, is one of the most economically important botanical families and includes many dietary and biologically valuable species such as cucumber, melon, watermelon and pumpkin. Since our group reported the first version of genome sequence for cucumber (*Cucumis sativus* L.) in 2009, the genomes for melon, watermleon, bottle gourd, pumpkin, bitter gourd, and mangosteen in the gourd family have been sequenced using IIlumina sequencing platform. Like the genomes of other cucurbit species, the cucumber genome assemblies were mostly assembled from the sequencing reads of Illumina sequencing technology, and there are approximately 150 Mb of missing sequences compared to the estimated genome size of 350 Mb. In addition, the contig and scaffold N50 sizes of the released cucumber genome assembly (V2.0) are only approximately 30.0 kb and 1.4 Mb, respectively. The missing sequences and low contiguity limit the applications of the genome assembly in comparative genomics and genetic research. Therefore, a high-quality and complete cucumber genome assembly is of great necessity.

In this study, we generated 16.2 Gb single-molecule real-time (SMRT) sequences, 20.2 Gb linked reads of ~50 Kb DNA fragments and 68.5 Gb long range contact reads (Hi-C) for cucumber. By combing these data, we obtained a significantly improved cucumber reference genome including only 174 contigs, adding an extra 29.0 Mb of sequences. Among these contigs, 89 were directly linked into the seven pseudo-chromosome sequences. Compared with the previous assembly, the contiguity was increased 234.8-fold in terms of contig N50 size, and 22.2-fold in terms of scaffold N50. The newly assembled regions abundantly show higher GC content, likely inaccessible to Illumina sequencing. The new assembly contains 1,248 full-length long terminal retrotransposons (LTRs) and 1,112 novel genes including 303 tandemly duplicated genes. Interestingly, multiple tyrosylprotein sulfotransferases (TPSTs), which is single copy gene in most other plants, were assembled, this feature may be specific in

cucurbits. Moreover, almost double the number of methylated sites were found in the new assembly in comparison to the previous one, providing a more complete genome for epigenetic analysis. This high-quality genome provides new features of cucumber genome, and will serve as a valuable resource for genetic research in cucumber as well as plant comparative genomics.

We would like to recommend the following colleagues as reviewers of the manuscript: Jordi Garcia-Mas (jordi.garcia@irta.cat) at Universitat Autònoma de Barcelona, Zhangjun Fei (zf25@cornell.edu) at Cornell Univerisy, Yiqun Weng (yiqun.weng@ars.usda.gov) at University of Wisconsin Madison.

Thank you in advance for considering this work.

Sincerely yours,

Zhonghua Zhang
Institute of Vegetables and Flowers, Chinese Academy of Agricultural Sciences
No. 12, Zhong Guan Cun Nan Da Jie, Beijing, 100081, China
Tel: +86-10-62117612
Mobile Phone: +8613699205910
Email: zhangzhonghua@caas.cn
