# Supplementary material for: A chromosome-scale genome assembly of cucumber (Cucumis sativus L.)
Source: Gigascience. 2019 Jun 18;8(6):giz072. doi: 10.1093/gigascience/giz072 (PMC6582320; doi:10.1093/gigascience/giz072)
Supplement: giz072_GIGA-D-18-00507_Revision_1 [file giz072_giga-d-18-00507_revision_1.pdf]

GigaScience

# A chromosome-scale genome assembly of cucumber (Cucumis sativus L.)

--Manuscript Draft--

| | |
|---|---|
| Manuscript Number: | GIGA-D-18-00507R1 |
| Full Title: | A chromosome-scale genome assembly of cucumber (Cucumis sativus L.) |
| Article Type: | Data Note |
| Funding Information: | China National Key Research and Development Program for Crop Breeding (2016YFD0100307) — Prof. Zhonghua Zhang<br>National Natural Science Foundation of China (31322047,31772304) — Prof. Zhonghua Zhang<br>National Youth Top-notch Talent Support Program in China (None) — Prof. Zhonghua Zhang<br>Science and Technology Innovation Program of Chinese Academy of Agricultural Science (None) — Prof. Zhonghua Zhang |
| Abstract: | Background<br>An accurate and complete reference genome assembly is fundamental for biological research. Cucumber is an important vegetable crop and model system for sex determination and vascular biology, and its draft genomes have been assembled using low coverage of Sanger sequences and high coverage of short Illumina sequences, but the incompleteness and low quality of these genomes limit their usage in comparative genomics and genetic research. Therefore, a high-quality and complete cucumber genome assembly is of great necessity.<br><br>Findings<br>We assembled single-molecule real-time (SMRT) long reads into a significantly improved cucumber reference genome containing 174 contigs with a total length of 226.2 Mb and an N50 of 8.9 Mb, providing an extra of 29.0 Mb sequences. Using 10X genomics, and high-throughput chromosome conformation capture (Hi-C) data, 89 contigs (~211.0 Mb) were directly linked into the seven pseudo-chromosome sequences. The newly assembled regions abundantly show higher GC or AT content, likely inaccessible to Illumina sequencing. The new assembly contains 1,374 full-length long terminal retrotransposons (LTRs) and 1,078 novel genes including 239 tandemly duplicated genes. For example, four tandemly duplicated tyrosylprotein sulfotransferases (TPSTs), which are a single copy gene in the previous assembly and most other plants, were assembled and predicted.<br><br>Conclusion<br>This high-quality genome represents novel features of the cucumber genome, and will serve as a valuable resource for genetic research in cucumber as well as plant comparative genomics. |
| Corresponding Author: | Zhonghua Zhang<br><br>CHINA |
| Corresponding Author Secondary Information: | |
| Corresponding Author's Institution: | |
| Corresponding Author's Secondary Institution: | |
| First Author: | Qing Li |
| First Author Secondary Information: | |
| Order of Authors: | Qing Li |

Powered by Editorial Manager® and ProduXion Manager® from Aries Systems Corporation

| | |
|---|---|
| | Hongbo Li |
| | Wu Huang |
| | Yuanchao Xu |
| | Qian Zhou |
| | Shenhao Wang |
| | Jue Ruan |
| | Sanwen Huang |
| | Zhonghua Zhang |
| **Order of Authors Secondary Information:** | |
| **Response to Reviewers:** | Dear Dr. Hongling Zhou<br><br>We greatly appreciate the dedication of the reviewers and the editor to help us to improve the manuscript. According to their comments, we have revised our manuscript and we provide a point-by-point response to the editor/reviewers' concerns and indicate how we have modified the manuscript.<br><br>Reviewer reports:<br><br>Reviewer #1:<br>24-1-2019<br><br>The authors have kindly offered access to the genome sequence and they also have explained that the genome sequence will be available in SRA by the time the paper is published. This completely solves the issue regarding the genome sequence. However, I still have doubts regarding the annotation. Will be the annotation made available in the genebank along with the sequence? As a GFF file in ICUGI? With a GFF file attached to the manuscript as supplementary material? Any of those options would be fine, because they will ease the use of the genome sequence by the community. I hope this issue is clarified soon and so the community can enjoy the great advance presented by the authors. I have no doubts that this paper will be a methodological reference.<br>Response:<br>We thank the reviewer for the comments and suggestions. The genome sequences and the corresponding annotations in GFF3 format are available at ICUGI ftp server: ftp://cucurbitgenomics.org/pub/cucurbit/genome/cucumber/Chinese_long/. To further ease the use of the genome by the community,  the genome and annotation can also be accessed via Genome Browser at ICUGI.<br>------------------------------------------------------------------------------------------------<br><br>21-1-2019<br><br>The reported V3.0 of the cucumber genome is not just a very useful tool for the cucumber community, but a great standard to follow in future genome assembly projects.<br>The methods used are appropriate and the results just great.<br>The manuscript is well written and clear. I have just detected a minor mistake, the authors have misspell telomeric as telemeric. (Although there could be more mistakes, I am not a native English speaker).<br>Response :<br>Thanks for your correction. we corrected all the misspell words in the manuscript.<br><br>Are the methods appropriate to the aims of the study, are they well described, and are necessary controls included?<br>Response:<br>We believe that we used appropriate methods to achieve our aims. Meta-assembled contigs were generated using CANU 1.7 [1] by combining results from two CANU and four FALCON/til-r assemblies with proper parameters (Supplemental Table S8). As a result, we obtained a more completed and accurate genome containing 174 contigs with a total size of 226.2 Mb and an N50 length of 8.9 Mb. To increase the accuracy of |

the contig sequences, the previously generated Illumina and Sanger reads (Supplemental Table S2) were aligned to the contigs. The potential sequence errors including single nucleotide polymorphism (SNP) and insertion/deletions (InDel) were corrected using Pilon [3] two times with the parameters: --fix all --chunksize 20000000 --mindepth 0.4 --K 65 --gapmargin 150000 --vcf --changes --tracks --minmq 10. The conflicting contigs with the orders of molecular markers from the four genetic maps were manually checked and split using the alignment results against the previous genome assembly (V2.0). Hi-C data were also aligned to the contigs for checking and correcting mis-assemblies. To clarify the methods more clearly, we added more details of genome assembly and annotation in the method section.

Given the experience of the authors I would encourage them to add a brief paragraph recommending which of the current technologies would they use in other future genome assembly projects, specially when the resources are limited and not all approaches could be carried out.
Response:
Thanks for the reviewer's suggestions. Some technologies in this manuscript have been used in several genome projects, and will be used for more species in the future. Recommending technologies for other genome projects should be a long story. In this manuscript, we think it is better to focus on the improved cucumber genome assembly.

I am also glad to see that the authors have used SRA and ICUGI to make the data available. This is a critical point, the usefulness of a genome assembly depends critically on the data availability, and in this case these are the best two avenues. I haven't been able to find the genome assembly, but I guess that it is not yet public because the manuscript is still under review, but, of course, the sequence should be made public in SRA by the time the manuscript is finally accepted. Also the genome sequence would be much less useful if the annotation is not provided in a easy to use format. So, with the sequence a GFF v3 file with the annotation should be provided.
Response:
The genome sequences and annotations in GFF format are both available in ICUGI under the ftp server:
ftp://cucurbitgenomics.org/pub/cucurbit/genome/cucumber/Chinese_long/. The corresponding sequences have been uploaded to SRA and will be made public.

Reviewer #2: This manuscript describes a much improved genome sequence of cucumber assembled from PacBio long reads, 10X linked reads and Hi-C contact maps. Overall the work is well performed and the manuscript is well written. I have made some minor changes and provided some comments directly in the word file of the manuscript, which is attached.
Response:
We thank the reviewer for corrections, and point-to-point responses were added in the revised manuscript.

Reviewer #3: Review of Li et al

The 'Data Note' describes a new assembly of the reference cucumber genome using long read PacBio data and provides a comparison with the previous Illumina based assembly. In general, the note describes the process well and details the main differences between the two assemblies.

As a minor comment, the introduction emphasizes the fact that the current available assemblies have ~150 Mb of missing data, yet this new assembly has only added at most 29 Mb, of which 28 Mb are repeat sequence. So although I agree that the authors have added to the available reference it is not as significant as one would have hoped with long read data.

I would recommend a few clarifications and improvements, which I think would be useful.
Response:
The highly repeated satellite sequences in cucumber, which account for ~30% (~105Mb) of the total nuclear DNA [10, 11], mainly distribute in telomeric, centromeric regions [12]. Given the highly repeated nature of the sequences, PacBio reads are not long enough to encompass these regions, and thus most of the satellite sequences

were lost in our assembly. In that case, most boundaries of these genomic segments were assembled, as we can see from the low-contact regions in Hi-C heat map (Main text, Fig. 2). This should be the reason that PacBio reads could not add more extra sequences as we expected. We added some description in the revised version.

It might be interesting to show the actual alignment of the two genomes as a figure.
Response:
We conducted whole-genome alignment between genomes 9930_V3 and 9930_V2 using nucmer program of MUMmer software (version 4.0.0beta2) [4] with parameters "-l 100 -c 100". Based on the alignments, the figure was plotted using python package: svgwrite (https://pypi.org/project/svgwrite/). Poorly-aligned and gap regions mainly reside in centromeres and heterochromatin. A translocation region in chromosome 5 between 9930 V3.0 and 9930 V2.0 was shown, which has been proved to be a mis-assembly in the genome of 9930 V2.0 [13, 14]. Two inversions in chromosome 4 and 6 were validated to be assembly errors in 9930 V2.0 because no obviously abnormal chromatin interaction was identified in these regions as shown in the Hi-C heat map (Main text, Fig. 2). We added the alignment figure into the supplementary figures (Supplemental Figure S2).

With respect to the assembly can the authors indicate how many contigs had to be split based on genetic data and/or HiC.
Response:
A total of three contigs were split based on the genetic map (Segkk155 Segkk31 Segkk90), and eight (Segkk107 Segkk113 Segkk118 Segkk148 Segkk17 Segkk35 Segkk45 Segkk56) due to the inconsistency with the Hi-C heat map.

For evaluation of the genome quality, how did the authors assess the errors, was this again using Pilon, since this is not described in the materials and methods. Also the authors only mention homozygous errors, what was the rate of heterozygous errors, these could be indicative of problematic assembly regions.
Response：
We used Pilon to polish the genome assembly. To evaluate the genome accuracy, we aligned previous Sanger and 6.0 Gb new Illumina reads onto the contigs using BWA [15]. Based on the alignments, genomic variations were called using GenomeAnalysisTK (http://www.broadinstitute.org/gatk) with default parameters. Considering that the sequenced cucumber 9930 is a high inbreed line, we would expect an extremely low heterozygous rate. And thus only homozygous variations were considered as errors. The heterozygous variations may derive from the incorrect alignments, or sequencing errors, or assembly errors. In fact, only 33 SNPs were identified as heterozygous by filtering the SNP index: 0.3<=SNP index<=0.7.

When looking at the gene annotation, the authors mention 2,693 newly predicted genes that were newly predicted compared to V2, but were present in V3. What were the error types that caused these genes not to be annotated in V2?
Response：
These can be classified into two categories. (1) The whole or partial sequences in V2.0 are absent because of limit of the Illumina sequencing technology. The incorrected or uncompleted assembly may destroy the gene structure and effect the prediction of genes. (2) More RNA-seq data and known protein sequences from uniport (Supplemental Table S4) were used, and some genes with new evidence were predicted.

Also in the materials and methods it is mentioned that there were genes annotated in V2 that were not annotated in V3 but were added to the V3 annotation. How many genes fell into this category and was there an explanation for this - errors in the V3 assembly?
Response：
We updated the annotation pipeline for predicting gene models in genome V3.0. A total of 2,617 genes in V2.0 were not predicted in V3.0. Their genomic sequences in V3.0 are identical with those in V2.0, and thus these should not be derived from the assembly errors. We are also confusing about the results. Aligning these genes against Uniprot protein database found that 84.5% can't find the homologs. We speculate that these may be due to the different sensitivity of updated pipeline. To

Powered by Editorial Manager® and ProduXion Manager® from Aries Systems Corporation

facilitating the community researchers to find the corresponding genes of V3.0 and V2.0, we still added these genes into the final gene models.

In general, the figure legends need improving (see below) but for the TPST gene figure the actual gene should be indicated in the figure. I am assuming it is b'? It does seem slightly odd that there is sequence data present in V2 at this point but it is not homologous with V3 and there is no apparent gap in the V2 assembly at this point - are the author sure that this is not an assembly error? Can they see single long reads that encompass multiple genes?
Response：
The figure legends have been revised. According to the genomic synteny between V2.0 and V3.0, the other three TPST genes are located in the gaps in V2.0. To further confirm the multiple copies of TPST, we aligned the PacBio long reads onto the assembled genome using minimap2 [17] with parameters "-ax map-pb", and show these result in Response Fig.3 using python package: svgwrite (https://pypi.org/project/svgwrite/). As a result, we found high coverage of PacBio reads that encompass multiple genes.

I am not sure the addition of methylation sites is meaningful since it basically reflects the increase in repeats.
Response：
Thanks for your comments. We remove this in the revised manuscript.

Figure 1 legend: Need to explain the different tracks, what are the colours and numbers indicating in the cytogenetic map? What are the orange and red marks? Etc.

Figure 3 legend: Unknown instead of Unknow. In c) what are the acronyms - TSR, PBS, PPT etc. In d) Which is the TPST gene?

Figure 5 legend: Not sure this figure is particularly informative, but what does the y-axis mean - it indicates Lengths (Mb) but in the text it is stated that the value is number of methylation sites.
Response:
We modified the manuscript according to the reviewer's suggestions.

Reviewer #4: Review of the manuscript titled "A chromosome-scale genome assembly of cucumber (Cucumis sativus L.)".
In this manuscript the authors presented a new PacBio chromosome scale genome assembly of the cucumber genome (called V3). The study employed data from different sequencing technologies (Sanger sequencing, PacBio, 10X, Hi-C and Illumina) for de-novo assembly, scaffolding, polishing and gene prediction analysis. Different genetic linkage maps were used for the anchoring and orienting the genome into chromosomes.
The manuscript offers a valuable resource including a high-quality chromosome scale reference genome, a new gene prediction and annotation. However, there are some points that should be appropriately addressed before the publication. See below.

Major: (In general, it would have been helpful if page number were added to the manuscript)
1. Will require some English revisions. Some clear examples: P5 L10-17. "has been served", this sentence in too long. P5 L28-36, sentence too long. P5 L40 "the applications" should be the application. There are more cases throughout the manuscript. I suggest to have a professional reviewer to edit the manuscript.
Response:
We modified the manuscript according to the reviewer's suggestions.

2. The PacBio Sequel raw data are not deposited into NCBI SRA. All the other data PacBio RSII, Hi-C, 10X data are deposited and available.
Response:
The PacBio data are available in NCBI SRA under accession SRP139269 (SRX5437838 for PacBio RSII and SRX5437837 for PacBio Sequel).

3. Missing references: in some parts of the manuscript, would be better to add

| | |
|---|---|
| | reference(s):<br>a. P7, L1: In cucumber, the repetitive sequences are estimated to account for 30%, (please add a ref)<br>b. P9, L27: Two predicted TPSTs in the wild cucumber genome also support multiple TPSTs in the cucumber genome (please add a ref)<br>c. P11, L2: NCBI's Short Read Archive (please add a ref or a link)<br>Response:<br>We modified the manuscript according to the reviewer's suggestions.<br><br>4. Although authors conducted some analysis for identification of novel predicted genes in the newly assembled genome v3 comparing to the v2 genome assembly, but no comparative analysis checking the improved gene models in v3 vs v2 (checking for fragmented gene models, split genes models etc.) is conducted. I would suggest to add this analysis in the manuscript, the improved genome could have improved the structure of predicted genes especially with the use of IsoSeq data.<br>Response：<br>Based on the alignments of genome V3.0 and V2.0, we identified 1,970 fragmented genes in V2.0 which correspond to 932 genes in V3.0. Conversely, 687 genes in V2.0 are split into 337 in V3.0. These were added in the revised manuscript (Supplemental Figure S5).<br>In the future, we will further improve the gene models using IsoSeq data.<br><br>5. Although authors report that 239 out of 1,078 novel genes were tandemly duplicated in V3.0 genome "Distribution analysis of the 1,078 novel genes in V3.0 along the pseudo-chromosomes showed that 239 are tandemly duplicated genes", however no description about how these analysis were conducted is presented in the manuscript.<br>Response：<br>To identify the tandemly duplicated genes in these novel genes, we detected all tandemly duplicated genes (TDGs) in V3.0 genome. Next, OrthoMCL [20] was used to identify orthologous groups in V3.0 genes. If the genes are in the same orthologous group and located next to each other on one chromosome, they are considered as TDGs. These were added into the method section.<br><br>6. Authors reported that four copies of TPST genes were predicted and identified in the v3 genome while this gene is in single copy in most other plants, proposing that this gene is responsible for some specific traits in cucurbits. In another place authors also report sequencing gaps around the flanking regions of this gene in melon and watermelons, but in none of the above cases no further information is reported. I believe that at least the ID of these genes, their chromosomal position/distribution, and some plots demonstrating what is presented should be presented.<br>Response：<br>Thanks for your suggestion. We added the information of TPST genes, include the gene ID, chromosomal position and function description as supplemental file (Supplemental Table S6).<br><br>7. Please also include the L50 number in Supplemental table S4<br>Response:<br>We have added the L50 number in Supplemental table S3. The L50 number of Scaffold and Super-scaffold are 8 and 4, respectively (Supplemental table S3).<br><br>8.In the introduction the authors indicated that about 150Mb assembly are missing in the V2 assembly. The authors should comment and could explore the PacBio data for this missing portion of the genome.<br>Response:<br>The highly repeated satellite sequences in cucumber, which account for ~30% (~105Mb) of the total nuclear DNA [10, 11], mainly distribute in telomeric, centromeric regions [12]. Given the highly repeated nature of the sequences, PacBio reads are not long enough to encompass these regions, and thus most of the satellite sequences were lost in our assembly. In that case, most boundaries of these genomic segments were assembled, as we can see from the low-contact regions in Hi-C heat map (Main text, Fig. 2). This should be the reason that PacBio reads could not add more extra sequences as we expected. We added some description in the revised version. |

Powered by Editorial Manager® and ProduXion Manager® from Aries Systems Corporation

| | |
|---|---|
| | Minor:<br>1. Legends in some of the plots are not clear<br>a. fig1: please specify that numbers (eg. 1-1) correspond to BAC-clone?<br>b. The font by the color legend (eg. the repeat type) is too small<br>c. I could not also find the Fosmid tracks in fig1.<br>d. Please separate the Hic Plot and Linkage map plots by a. and b. and reflect this change in the figure legend.<br>2. P5, L1: "chromosome scale assembly" => "chromosome scale genome assembly"<br>3. P6, L30: "additional 19.1 Mb sequences": please specify that is comparing to the v2 genome assembly…<br>4. P6, L32: "lacking contact information" => "lacking Hi-C contact information"<br>5. P7, L21: the sentence is confusing. Please rephrase (e.g. using such technologies, the contiguity of the cucumber genome assembly can be significantly increased)<br>6. P7, L36: "genetics": you mean "genetic studies"?<br>7. P8, L30: SRA ID is missing<br>8. P8, L51: "markedly" => "remarkably" or "significantly"<br>9. P8, L60: "Dating"? please rephrase, the message is not clear<br>10. P9 L10-14:<br>a. Move the "(Supplemental table S7)" to the end of the paragraph<br>Response:<br>We modified the manuscript according to the reviewer's suggestions.<br><br>b. if you are listing those newly assembled, please also list those newly predicted with the coordinates and structure if possible<br>Response：<br>We added the information of these genes, include the gene ID, chromosomal position and function description as supplemental file (Supplemental Table S6).<br><br>c. a simple statistic for the newly predicted genes (avg. ln, average # of exons etc.) wuold be useful<br>Response：<br>We compared the average length and number of exons and intron of genes between the whole set of genes and newly predicted ones. The average number of exons per gene in whole and newly genes are five and four, respectively. They were added into the revised manuscript (Supplemental Figure S4).<br><br>11. P9, L34: "contact" is not clear<br>12. P9, L43-45: cannot be mapped? What do you mean by this? The use of Illumina reads here is confusing and not clear. Authors might want to rephrase the sentence.<br>13. P11, L2: "NCBI's Small Read Archive" => "NCBI Short Read Archive"<br>14. P11, L57-58: "(See supplemental table S3 for detailed information)", Please follow the same format when referring to the supplemental information<br>15. Please also include the L50 number in Supplemental table S4<br>Response:<br>We modified the manuscript according to the reviewer's suggestions.<br><br>Reference<br>1.Koren S, Walenz BP, Berlin K, Miller JR, Bergman NH and Phillippy AM. Canu: scalable and accurate long-read assembly via adaptive k-mer weighting and repeat separation. Genome Res. 2017;27 5:722-36. doi:10.1101/gr.215087.116.<br>2.Lam KK, LaButti K, Khalak A and Tse D. FinisherSC: a repeat-aware tool for upgrading de novo assembly using long reads. Bioinformatics. 2015;31:3207-9.<br>3.Walker BJ, Abeel T, Shea T, Priest M, Abouelliel A, Sakthikumar S, et al. Pilon: an integrated tool for comprehensive microbial variant detection and genome assembly improvement. PLoS One. 2014;9:e112963.<br>4.Kurtz S, Phillippy A, Delcher AL, Smoot M, Shumway M, Antonescu C, et al. Versatile and open software for comparing large genomes. Genome Biol. 2004;5:R12.<br>5.Yang LM, Koo DH, Li YH, Zhang XJ, Luan FS, Havey MJ, et al. Chromosome rearrangements during domestication of cucumber as revealed by high-density genetic mapping and draft genome assembly. Plant J. 2012;71:895-906.<br>6.Ren Y, Zhang Z, Liu J, Staub JE, Han Y, Cheng Z, et al. An integrated genetic and cytogenetic map of the cucumber genome. PLoS One. 2009;4:e5795. |

Powered by Editorial Manager® and ProduXion Manager® from Aries Systems Corporation

| | |
|---|---|
| | 7.Zhang WW, Pan JS, He HL, Zhang C, Li Z, Zhao JL, et al. Construction of a high density integrated genetic map for cucumber (Cucumis sativus L.). Theor Appl Genet. 2012;124:249-59.<br>8.Zhou Q, Miao H, Li S, Zhang S, Wang Y, Weng Y, et al. A Sequencing-Based Linkage Map of Cucumber. Mol Plant. 2015;8:961-3.<br>9.Tang H, Zhang X, Miao C, Zhang J, Ming R, Schnable JC, et al. ALLMAPS: robust scaffold ordering based on multiple maps. Genome Biol. 2015;16:3. doi:10.1186/s13059-014-0573-1.<br>10.Ganall M and Hemleben R. Organization and sequence analysis of two related satellite DNAs in cucumber (Cucumis sativus L.). Journal of Molecular Evolution. 1986;23 1:23-30.<br>11.Ganall M and Hemleben R. Insertion and amplification of a DNA sequence in satellite DNA of Cucumis sativus L. (cucumber). Theoretical and Applied Genetics. 1988;75 2:357-61.<br>12.Han YH, Zhang ZH, Liu JH, Lu JY, Huang SW and Jin WW. Distribution of the tandem repeat sequences and karyotyping in cucumber (Cucumis sativus L.) by fluorescence in situ hybridization. Cytogenet Genome Res. 2008;122 1:80-8. doi:10.1159/000151320.<br>13.Zhou Q, Miao H, Li S, Zhang S, Wang Y, Weng Y, et al. A sequencing-based linkage map of cucumber. Molecular plant. 2015;8 6:961-3.<br>14.Sun JY, Zhang ZH, Zong X, Huang SW, Li ZY and Han YH. A high-resolution cucumber cytogenetic map integrated with the genome assembly. Bmc Genomics. 2013;14  doi:Artn 46110.1186/1471-2164-14-461.<br>15.Li H and Durbin R. Fast and accurate short read alignment with Burrows-Wheeler transform. Bioinformatics. 2009;25:1754-60.<br>16.Gotoh O. A space-efficient and accurate method for mapping and aligning cDNA sequences onto genomic sequence. Nucleic Acids Res. 2008;36:2630-8.<br>17.Li H. Minimap2: pairwise alignment for nucleotide sequences. Bioinformatics. 2018;34 18:3094-100. doi:10.1093/bioinformatics/bty191.<br>18.Li H and Durbin R. Fast and accurate short read alignment with Burrows-Wheeler transform. Bioinformatics. 2009;25 14:1754-60. doi:10.1093/bioinformatics/btp324.<br>19.Thorvaldsdottir H, Robinson JT and Mesirov JP. Integrative Genomics Viewer (IGV): high-performance genomics data visualization and exploration. Brief Bioinform. 2013;14 2:178-92. doi:10.1093/bib/bbs017.<br>20.Li L, Stoeckert CJ, Jr. and Roos DS. OrthoMCL: identification of ortholog groups for eukaryotic genomes. Genome Res. 2003;13:2178-89. |
| Additional Information: | |
| Question | Response |
| Are you submitting this manuscript to a special series or article collection? | No |
| **Experimental design and statistics**<br><br>Full details of the experimental design and statistical methods used should be given in the Methods section, as detailed in our Minimum Standards Reporting Checklist. Information essential to interpreting the data presented should be made available in the figure legends.<br><br>Have you included all the information requested in your manuscript? | Yes |
| **Resources** | Yes |

Powered by Editorial Manager® and ProduXion Manager® from Aries Systems Corporation

| | |
|---|---|
| A description of all resources used, including antibodies, cell lines, animals and software tools, with enough information to allow them to be uniquely identified, should be included in the Methods section. Authors are strongly encouraged to cite Research Resource Identifiers (RRIDs) for antibodies, model organisms and tools, where possible.<br><br>Have you included the information requested as detailed in our Minimum Standards Reporting Checklist? | |
| **Availability of data and materials**<br><br>All datasets and code on which the conclusions of the paper rely must be either included in your submission or deposited in publicly available repositories (where available and ethically appropriate), referencing such data using a unique identifier in the references and in the “Availability of Data and Materials” section of your manuscript.<br><br>Have you have met the above requirement as detailed in our Minimum Standards Reporting Checklist? | Yes |

Manuscript

Click here to access/download;Manuscript;cucumber_paper_GigaScience.doc

Click here to view linked References

# A chromosome-scale genome assembly of cucumber (*Cucumis sativus* L.)

Qing Li[1,#], Hongbo Li[1,#], Wu Huang[1,2,#], Yuanchao Xu[1], Qian Zhou[1,2], Shenhao Wang[3], Jue Ruan[2], Sanwen Huang[2], Zhonghua Zhang[1,*]

[1] Institute of Vegetables and Flowers, Chinese Academy of Agricultural Sciences, No.12, Haidian District, Beijing 100081, China.

[2] Agricultural Genomics Institute at Shenzhen, Chinese Academy of Agricultural Sciences, No. 7, Pengfei Road, Dapeng District, Shenzhen 518124, China.

[3] College of Horticulture, Northwest A&F University, Yangling, Shanxi 712100, China

[#] These authors contributed equally to this work.

[*] Address correspondence to Zhonghua Zhang (zhangzhonghua@caas.cn)

**Author for Contact details:**

Institute of Vegetables and Flowers

Chinese Academy of Agricultural Sciences

No.12 Zhongguancun South St., Haidian District Beijing 10081, P.R.China

Tel: +86-10-62117612

Mobile Phone: +8613699205910

Email: zhangzhonghua@caas.cn

## Abstract

### Background

An accurate and complete reference genome assembly is fundamental for biological research. Cucumber is an important vegetable crop and model system for sex determination and vascular biology, and its draft genomes have been assembled using low coverage of Sanger sequences and high coverage of short Illumina sequences, but the incompleteness and low quality of these genomes limit their usage in comparative genomics and genetic research. Therefore, a high-quality and complete cucumber genome assembly is of great necessity.

### Findings

We assembled single-molecule real-time (SMRT) long reads into a significantly improved cucumber reference genome containing 174 contigs with a total length of 226.2 Mb and an N50 of 8.9 Mb, providing an extra of 29.0 Mb sequences. Using 10X genomics, and high-throughput chromosome conformation capture (Hi-C) data, 89 contigs (~211.0 Mb) were directly linked into the seven pseudo-chromosome sequences. The newly assembled regions abundantly show higher GC or AT content, likely inaccessible to Illumina sequencing. The new assembly contains 1,374 full-length long terminal retrotransposons (LTRs) and 1,078 novel genes including 239 tandemly duplicated genes. For example, four tandemly duplicated tyrosylprotein sulfotransferases (TPSTs), which are a single copy gene in the previous assembly and most other plants, were assembled and predicted.

### Conclusion

This high-quality genome represents novel features of the cucumber genome, and will serve as a valuable resource for genetic research in cucumber as well as plant comparative genomics.

### Keywords

cucumber; PacBio; Hi-C; genomics; chromosome-scale assembly

## Data Description

### Introduction

An accurate and complete reference genome assembly is essential for genetic and genome-wide studies of both individual species and multiple species. For cucumber (*Cucumis Sativus* L.), which is an important vegetable crop and has been served as model plant for sex determination and vascular biology. Four genome assemblies of cucumber including one wild and three cultivated accessions have been released since 2009 [1-5], which were mainly assembled using Illumina short sequences. In comparison to the estimated genome size of 350 Mb [4], these assemblies range from 197 to 203 Mb in length, and therefore still have approximately 150 Mb of missing sequences. According to the cytogenetic and sequence information, about 100 Mb satellite sequences, which consist of very large arrays of tandemly repeated DNAs with the length of 177 or 366 bp, are mainly present in cucumber centromeric/telomeric regions and cannot be assembled using the current sequencing technology. Besides the satellites, there are still lots of missing sequences in the current assemblies, and this will hamper the genetic-based gene isolation, identification of variations and epigenetic modification sites, and comparative analyses on the population level and across closely related species. Moreover, the contig and scaffold N50 sizes of the released cucumber genome assembly (V2.0) are only 30.0 kb and 1.4 Mb, respectively [2], leaving more than 10,000 gaps. The missing sequences and low contiguity limit the applications of the genome assembly in comparative genomics and genetic research. Therefore, a high-quality and complete cucumber genome assembly is of great necessity.

Repetitive sequences such as transposable elements (TEs) pose the largest challenge for a high-quality genome assembly, especially for plant genomes [6]. The nature of short reads from the Illumina sequencing technology often collapsed the similar repetitive sequences into a single copy. To overcome this limitation, single-molecule real-time (SMRT) sequencing technologies such as Pacific Biosciences (PacBio) and Oxford Nanopore, which generate long reads of more than 10kb in size, have been advanced significantly in recent years. For several plants and animals, high-quality genome assemblies have been generated using these technologies [7-12]. In cucumber, the repetitive sequences are estimated to account for 30% [4], it is also necessary to improve the assembly using the long-read sequencing technology.

Scaffolding technologies are critical for accurately ordering and orienting assembled contigs. For the past decades, read information from a range of mate-pair libraries with different insert sizes have been widely used for scaffolding. However, preparing mate-pair library is expensive, and the read information are sometimes also confused by repetitive elements. In recent years, new cost-effective and accurate technologies including 10X genomics, optical mapping and high-throughput chromosome conformation capture (Hi-C) have been developed, and they can provide long-range contiguity information ranging from ~50 kb to several mega-bases which aid in scaffolding [11, 13-15]. These new technologies will benefit the contiguity of cucumber genome assembly to a large extent.

Here, we assembled a significantly improved reference genome assembly for cucumber by combining the read sequences of PacBio, 10X genomics and Hi-C. Comparison of the new assembly to the previously released version revealed a significant improvement in genome completeness and contiguity. This work represents numerous novel sequences such as protein-coding genes and intact retrotransposons, and thus provides a robust reference sequences for genetics in cucumber.

**Genome sequencing and assembly**

We sequenced the ‘Chinese long’ inbred line 9930, the genome of which was assembled several years ago based on Illumina and Sanger sequences [2, 4], using new technologies including PacBio, 10X genomics, and Hi-C. A total of 16.2 Gb PacBio read sequences representing 46.2-fold genome coverage with a sub-read N50 length of 10.8 kb were generated **(Supplemental Table S1)**. To fully utilize the PacBio data, meta-assembly was performed based on two CANU pre-assemblies and four FALCON pre-assemblies, resulting in a total of 195 contigs spanning 232.3 Mb in length. Comparing the final assembly with the pre-assemblies showed the complementarity of the six initial assemblies (**Supplemental Figure S1**). Assembled contigs containing potential bacteria and plastid contamination were eliminated. Using FinisherSC [16], we aligned the raw PacBio reads to the resulted contigs and merged the contigs which could be connected, and the gaps were filled by reads. To correct any potential sequencing errors, Illumina sequences **(Supplemental Table S2)** were mapped to the assembled sequences.

A total of 49,157 single base pair substitutions and 156,931 small InDels were corrected using Pilon [17]. Using four genetic maps [3, 18-20], the obvious assembly errors were detected, and these contigs were split. All contigs were aligned against the previous assembly (V2.0), and no obvious errors were observed. Finally, a total of 174 contigs were obtained with a total length of 226.2 Mb and an N50 length of 8.9 Mb **(Supplemental Table S3)**, an approximately 234.8-fold improvement in contiguity compared with the previous assembly.

To build scaffolds, we generated 20.2 Gb linked reads with long-range information of 50 Kb DNA fragments using 10X Genomics platform and 68.5 Gb long-range contact reads from Hi-C **(Supplemental Table S1)**. Linked reads connected 174 contigs into 157 scaffolds, resulting in an N50 length of 11.5 Mb. On the basis of these scaffolds, we further linked them into 85 super-scaffolds with an N50 of 31.1 Mb using the Hi-C data **(Supplemental Table S3)**. Among them, seven super-scaffolds with a total length of 211.0 Mb correspond directly to the seven chromosomes of cucumber, thus providing additional 19.1 Mb sequences for the seven pseudo-chromosome sequences (**Figure 1**) relative to the genome V2.0. Because of lacking Hi-C contact information, the remaining 78 super-scaffolds (15.2 Mb) cannot be clustered into any of the seven chromosomes, suggesting that these could be mainly covered by repetitive sequences. Therefore, we presented here a more complete pseudo-chromosome sequences for the cucumber reference genome.

**Evaluation of the genome quality**

To assess the quality of the new genome assembly (V3.0), we mapped 6.0 Gb new Illumina and previous Sanger reads (**Supplemental Table S2**) to the final assembled sequences. Only 53,179 substitutions and 30,546 small InDels were identified as homozygous variations (index >0.9), and thus, the error rates for single base pair and small InDels are estimated to be below 0.00024 and 0.00014, respectively, which indicates a high accuracy of V3.0 at the single base-pair level.

The genome sequences are highly consistent with the genetic maps and Hi-C data, which show the high accuracy of contiguity for the assembly (**Figure 2**). The orders of genetic markers are consistent with the assembly sequences with a correlation coefficient of 0.98 on average. From

the long ranges contact information of Hi-C, we can see that most regions show close contact with nearby sequences, and only the centromeric/telomeric regions have few contacts with other genomic segments.

Integration of the genome assembly with the cytogenetic map [21] reveals the high completeness of V3.0 (**Figure 1**). For all the seven chromosomes, most of the centromeric and telomeric sequences are absent. The main components of the centromere are satellite type III, and they are detected at the ends of the super-scaffolds around the centromeres, indicating the boundaries of them. Among the 14 ends of the seven chromosomes, 13 have satellite type I/II/IV, which constitute the majority of the telomere, indicating the boundary of the telomere. These assembly consists of almost all the genome sequences except for the centromeric and telemetric regions which are largely constituted by the satellite sequences account for ~30% (~105Mb) of total nuclear DNA [22, 23] and cannot be assembled using current sequencing technologies [24].

We also explored the consistency between V2.0 and V3.0 genomes using whole-genome alignment **(Supplemental Figure S2)**. It is apparent that lots of novel sequences are inserted into the genome V3.0. The distal sequences on the chromosome 5 of V2.0 are translocated to the correct position in V3.0, this is consistent with the previous report [20, 21]. In addition, two inversions on the chromosome 4 and 6, which are assembly errors in V2.0, were corrected in V3.0 which are supported by the data in the Hi-C heat map **(Figure 2)**.

To assess the completeness of gene space, we downloaded 121.7 Gb of RNA-seq sequences generated from 39 samples (**Supplemental Table S4,** SRA ID), including a variety of tissues such as root, stem, leaf, flower, and fruit, and mapped them to assemblies V2.0 and V3.0, respectively. Compared to V2.0, 3.2 Gb additional RNA-seq sequences were mapped on V3.0, resulting in 932.2 Kb additional expressed genomic regions. Therefore, this new assembly represents a higher completeness in gene space.

**Genome annotation reveals novel repetitive sequences and genes**

In V3.0, we identified a total of 82.0 Mb, representing 36.43% of the genome, as repetitive sequences (**Supplemental Table S5**), approximately 27.6 Mb more than those predicted in V2.0 (54.4 Mb). Among the repetitive sequences, the long terminal retrotransposons (LTRs) are the most abundant and the size of them increased remarkably in V3.0 (**Figure 3A**). A total of 1,374 full-length LTRs (FL-LTRs) were predicted in V3.0, five times more than that (267) in V2.0 (**Figure 3B**). Most of these FL-LTRs were partially assembled in V2.0, thus they were not annotated as FL-LTRs. For example, a FL-LTR on chromosome 1 was not predicted because of the absence of pol-domain and long terminal repeats in V2.0 (**Figure 3C**). The insert times analysis of these FL-LTRs reveals that most of them occurred recently in cucumber, and this result explains the complexity of these regions during the assembly process (**Supplemental Figure S3**) [6].

A total of 24,317 protein-coding genes in V3.0 were predicted by combining three methods, including *ab initio*, protein homology-based, and transcriptome sequences, using the EVM pipeline [25]. In comparison to the predicted genes in V2.0, 1,078 genes (**Supplemental Table S6**) were newly assembled in V3.0, and 2,693 were newly predicted in V3.0 but not predicted in V2.0 due to sequencing gaps or errors or the bias of annotation pipeline. Among the newly assembled genes, 931 are expressed in at least one of the above 39 samples with RNA-seq data, indicating their high reliability. These genes are characterized by short average length and less average exon number compared to all genes (**Supplemental Figure S4**). Based on the alignments of genes in V3.0 and V2.0, we also identified 1,970 fragmented genes in V2.0 which correspond to 932 genes in V3.0. Conversely, 687 genes in V2.0 are split into 337 in V3.0 (**Supplemental Figure S5**). Distribution of the 1,078 novel genes in V3.0 along the pseudo-chromosomes indicates that 239 are tandemly duplicated genes. For example, in V2.0, only one tyrosylprotein sulfotransferase (TPST), which is a single copy gene in most plants such as Arabidopsis and tomato, was predicted, but four tandemly duplicated genes were obtained in V3.0 (**Figure 3D**). Two predicted TPSTs in the wild cucumber genome [5] also support multiple TPSTs in the cucumber genome [4]. Therefore, the new genome provides a more complete gene set for functional genomic research in cucumber.

## Features of novel sequences in assembly V3.0

To explore the features of the novel sequences in the new assembly, we analyzed the novel sequences using Illumina reads as well as the newly assembled genes. The sequences with GC content of approximately 32.8% are dominantly abundant on the whole genome level; however, the GC content distribution of novel sequences shows peak at approximately 35.0% (**Figure 4**). The newly assembled genes also show a similar GC distribution (**Supplemental Figure S6**). These suggest that a number of sequences with abnormal GC content could be only generated using the PacBio sequencing technology. Among the new genes, more than 30 domains such as pectinesterase inhibitor (IPR034086, pectinesterase inhibitor, plant; IPR006501, pectinesterase inhibitor domain, etc.), zinc finger, and CCHC-type domain (IPR036875) are significantly enriched ($p<0.005$) (**Supplemental Table S7**). These results indicate that the PacBio sequencing technology is advantageous for some types of genes.

## Conclusion

By combining the long-read sequences of PacBio, long range information of 10X genomics and long range contact reads of Hi-C, a high quality cucumber reference genome is provided for the community. A large number of repetitive sequences and genes have been identified and added to the assembly, especially for sequences with high GC or high AT content and genes with certain domains. More tandemly duplicated genes were assembled in the new genome. These data provide a valuable resource for comparative genomics, epigenetics, gene isolation, and transposon research.

## Materials and Methods

### Genome sequencing.

*PacBio sequencing*: High quality genomic DNA was extracted from young leaves of 'Chinese long' inbred line 9930 using a modified CTAB method [26]. Genomic DNA was sheared to a size range of 15-40 kb by a Megaruptor (Diagenode) device, and then was used for Single-Molecule Real Time (SMRT) library preparation as recommended by Pacific Biosciences. Two SMRTbell$^{TM}$ templates were prepared in 2014 and 2016, respectively. The first library was

sequenced on PacBio RSII platform, and 1,470,953 reads (11.0 Gb) were generated. The second library was sequenced on PacBio Sequel platform, and 628,153 reads (5.2 Gb) were generated.

*10X genomics linked-read sequencing*: A total of 0.3 ng high-molecular-weight DNA was prepared and loaded onto Chromium Controller chip with 10X Chromium reagents and gel beads following the recommended protocols (https://support.10xgenomics.com/de-novo-assembly). On average, the loaded DNA molecule is ~50 kb in length. There are about 1 million droplets on a Chromium Controller chip. Within each droplet, several DNA molecules were sheared, and the sheared DNA fragments were tagged with the same barcode. Then all barcoded DNA fragments within these droplets were sequenced on an Illumina Hiseq X Ten sequencer to produce 2 ×150 bp paired-end sequences.

*Hi-C read sequencing*: Leaves of cucumber line 9930 were fixed with 1% formaldehyde solution, and chromatin was cross-linked and digested using restriction enzyme HindIII. The 5' overhangs were filled in with biotinylated nucleotides, and then free blunt ends were ligated. After ligation, crosslinks were reversed and the DNA purified from protein. Purified DNA was treated to remove biotin that was not internal to ligated fragments. The DNA was then sheared into fragment size of ~350 bp. Two sequencing libraries were prepared as described previously [27]. The libraries were sequenced on an Illumina HiSeq X Ten platform. For each library, a total of 223 million paired-end reads of 150 bp in length were generated, representing 195.5-fold coverage of cucumber genome in total. A detailed quality control (QC) report for the Hi-C sequencing was yielded by HiCUP [28].

**Genome assembly.**

*De novo assembly of PacBio reads*: We performed meta-assembly of the PacBio reads from SMRT sequencing as previously described [29]. In summary, meta-assembled contigs were generated using CANU 1.7 [30] by combining results from two CANU and four FALCON/til-r assemblies in which the number of contigs range from 589 to 1,094 with a contig N50 length between 2.4Mb and 3.6Mb **(see Supplemental Table S8 for detailed information)**. Assembled contigs were aligned against the bacterial genomes and the cucumber plasmid genomes from GenBank using BLAST [31]. If more than 70% of a contig shows >95% identity with bacterial or plasmid genomes, it was eliminated.. Using the FinisherSC pipeline [16] with default

parameters, we determined the contigs that could be connected by raw PacBio reads, and gaps were filled by reads. To increase the accuracy of the contig sequences, the previously generated Illumina and Sanger reads **(Supplemental Table S2)** were aligned to the contigs. Potential sequence errors in the form of single base pair substitution and insertion/deletion (InDel) were corrected using Pilon [17] two times with the parameters: --fix all --chunksize 20000000 --mindepth 0.4 --K 65 --gapmargin 150000 --vcf --changes --tracks --minmq 10. In addition, the corrected contigs were aligned against the previous genome assembly (V 2.0) using MUMmer [32] with default parameters and were anchored onto the seven linkage groups of the four genetic maps [3, 18-20] using ALLMAPS [33]. The conflicting contigs with the orders of molecular markers from the four genetic maps were manually checked and split using the alignment results against the previous genome assembly (V2.0). Hi-C data were also aligned to the contigs for checking and correcting mis-assemblies.

*Scaffold construction*: The final contigs were connected into scaffolds using 10X linked reads by ARKS [34] with following parameters: m=20-20000 threads=20 a=0.9. By aligning the sequences of genetic markers and Hi-C data to the assembled scaffolds, we split the scaffolds conflicting with the orders of molecular markers or long-range contact information. Then, the chromosome-level super-scaffolds were constructed on the basis of the genome-wide chromatin interaction information using 3d-dna pipeline [13] with the parameters: -m haploid -i 15000 -r 0, which resulted in seven chromosome-level super-scaffolds, representing seven pseudo-chromosomes of cucumber, and 78 short-length super-scaffolds that cannot be clustered for lacking interactions with the seven chromosome-level super-scaffolds.

*Pseudo-chromosome construction*: The seven chromosome-level super-scaffolds were anchored onto the seven linkage groups of the four genetic maps [3, 18-20] and orientated into the seven pseudo-chromosomes using ALLMAPS [33] with default parameters. Furthermore, the pseudo-chromosomes were integrated with the cytogenetic map by mapping the marker sequences and satellite sequences (Type I/II/III/IV) onto the assembly using BLASTN (v2.2.15) at an e-value cutoff of 0.05. The satellite sequences are abundantly distributed within centromeric and telomeric regions so the positions of centromere and telomere were marked accordingly.

**Genome annotation.**

*Repetitive sequences*: RepeatModeler (http://www.repeatmasker.org/RepeatModeler/) was used to perform *de novo* searching for repetitive sequences in the genome assembly V3.0 and V2.0. Identified repeats and the TIGR plant repeat database (http://plantrepeats.plantbiology.msu.edu) were then used to identify and mask the repeats in V3.0 and V2.0 using RepeatMasker (http://www.repeatmasker.org). The repeats were classified into different types based on the annotation of RepeatMasker. Moreover, full length LTR retrotransposons (FL-LTRs) were identified using LTR_Finder (v 1.0.6) [35] with the command line 'ltr_finder genome.fa -s tRNAdb/Athal-tRNAs.fa -a ps_scan > result.txt'. The long terminal repeats of FL-LTRs were aligned with MUSCLE [36] and the nucleotide distance (D) was estimated using the Kimura two-parameter (K2p) (transition-transversion ratio) criterion as implemented in the distmat program of the EMBOSS [37] package (v 6.6.0). The insertion time (T) of an LTR retrotransposon was calculated using the formula: $T= D/2\mu$, where the $\mu$ is 4.5e-9, and rate of nucleotide substitution ($\mu$) was inferred according to Nystedt's method [38].

*Protein-coding genes*: Putative protein-coding genes were predicted using EVidenceModeler [25] by integrating several *ab initio* gene predictors including Augustus (http://augustus.gobics.de), GlimmerHMM [39] and SNAP [40], RNA-seq data and homologous proteins from other plant species. A total of 121.7 Gb RNA-seq sequences generated from 39 samples (**Supplemental Table S4**) including a variety of tissues such as root, stem, leaf, flower and fruit [2, 41, 42] were used for gene prediction. In addition, genes in V2.0 that are not predicted in V3.0 were added into the protein-coding gene set using Spaln [43].

*Functional annotation of protein-coding genes*: All predicted proteins were aligned against UniProt (www.uniprot.org) and Arabidopsis proteins (www.arabidopsis.org). Annotation of the best matched protein is assigned to the predicted proteins. Functional annotation was also performed using InterProScan. Gene ontology (GO) terms were assigned according to InterPro classification.

**Comparative analyses between the assembly V2.0 and V3.0.**

*Evaluating the accuracy of genome:* We accessed the accuracy of genome assembly quality by aligning previous Sanger and 6.0 Gb new Illumina reads to the corrected contigs using BWA [44]. Genomic variations were called using GenomeAnalysisTK (http://www.broadinstitute.org/gatk) with default parameters. Considering that the sequenced cucumber 9930 is a high inbreed line, we would expect an extremely low heterozygous rate: index>0.9.

*Whole-genome alignment:* We conducted whole-genome alignment for genomes of V3.0 and V2.0 (**Supplemental Figure S2**) using the nucmer program of MUMmer software (version 4.0.0beta2) [32] with parameters "-l 100 -c 100", After that, we used show-coords to show and filter the result of nucmer with parameter: "o -l -r -I 99 -L 1000". The figure was plotted using python package: svgwrite (https://pypi.org/project/svgwrite/).

*Mapping RNA-seq data:* All downloaded RNA-seq reads were mapped to the genome assembly V2.0 and V3.0 using TopHat 2.1.1 with default parameters [45]. On the basis of the alignments, the transcripts were assembled using Cufflinks 2.2.1 without genome guidance [46].

*Identification of novel genes in V3.0*: The coding sequences of predicted genes in V3.0 were aligned against those in V2.0 using BLAST and *vice versa*. Combining with syntenic information, gene pairs were determined based on the alignments. The corresponding genes are classified into three categories: one to one, one to multiple, multiple to multiple by using python scripts. For the remaining genes in V3.0, the gene sequences including intronic sequences were aligned against V2.0 genome. If the matched region did not meet the threshold of coverage >50% and identity >95%, the query gene was considered as novel in V3.0. Otherwise, the sequences of matched regions in V2.0 were extracted and then aligned against the V3.0 genome. If the matched region in V3.0 covers the whole query gene and the identity is more than 95%, we considered the query gene in V3.0 had counterpart in V2.0 genome but it was not predicted. Genes which were not classified above are also considered as novel genes.

*GC content:* The genome sequences were split into multiple non-overlapping 100 kb windows. For each window, the GC content was calculated using a python script. For the novel sequences in V3.0, the GC content of each DNA fragment was independently calculated.

*InterPro domain enrichment:* To identify enriched InterPro domains for the novel genes, the observed number of each domain among novel genes were compared with the expected number among the whole genes using chi-square test. InterPro domains with p-values < 0.005 are regarded as enriched.

*Identification of tandemly duplicated genes (TDG):* OrthoMCL [47] was used to identify orthologous groups in V3.0 genes. If the genes are in the same orthologous group and located next to each other on one chromosome, they are considered as TDGs.

**Availability of supporting data**

The sequence data have been deposited in the NCBI Sequence Read Archive with accession number SRP139269 (PacBio: SRX5437838 and SRX5437837; Hi-C: SRX3918394, SRX3918395; 10X: SRX3918396). The genome sequences and the corresponding annotations in GFF3 format are both available in ICUGI under the ftp server: ftp://cucurbitgenomics.org/pub/cucurbit/genome/cucumber/Chinese_long/. All supplemental figures and tables are provided in Supplemental.docx and Supplemental.xlsx.

**Additional files**

Supplemental.docx: Supplemental information contain figures and tables.

Supplemental TableS6.xlsx: Annotation of the newly assembled genes in V3.0.

Supplemental TableS7.xlsx: Enriched InterPro terms for the novel genes in V3.0.

**Abbreviations**

TE: transposable element; SMRT: single-molecular real-time; PacBio: Pacific Biosciences; Hi-C: high-throughput chromosome conformation capture; InDels: insertion or deletion of bases; SNP: single-nucleotide polymorphism; RNA-seq: RNA sequencing; SRA: Sequence Read Archive; LTR: long terminal retrotransposons; FL-LTR: full-length LTR; EVM: EVidenceModeler; TPST: tyrosylprotein sulfotransferase; BLAST: Basic Local Alignment Search Tool; NCBI: National Center for Biotechnology Information; TSP: Target Site Repeat; PBS: Primer Binding Site; PPT: Primer Polypurine Tract; IN: Integrase; RT: Reverse Transcriptase.

**Competing interests**

All authors report no competing interests.

**Acknowledgments**

We would like to thank Qingyong Yang for the help of the analysis of Hi-C data.

**Author contributions:**

Z.Z. conceived and designed the research. S.W. and W.H. participated in the material preparation. W.H., J.R. and H.L. performed the assembly and scaffolding. Q.L., H.L., Q.Z., and Y.X. performed the annotation and comparative analysis. Z.Z. wrote the manuscript. S.H. revised the manuscript.

**Funding information:**

This work was supported by China National Key Research and Development Program for Crop Breeding (2016YFD0100307 to Z.Z.), National Science Fund for Excellent Young Scholars (31322047 to Z.Z.), National Natural Science Foundation of China (31772304 to Z.Z.), and the National Youth Top-notch Talent Support Program in China (Z.Z.). This work was also supported by the Science and Technology Innovation Program of Chinese Academy of Agricultural Science (CAAS-ASTIP-IVFCAAS).

**Figure legends**

**Figure 1. Landscape of the seven pseudo-chromosome sequences.** All included contigs are shown. The cytogenetic map [21] is integrated with the sequences. Arrows mark the positions of the centromeres. The distribution of satellite and repetitive sequences along the contigs is illustrated below. Fosmid clones are marked in green and red on the seven chromosomes, and the imaginary lines connect the physical locations and approximate locations of assembled chromosomes.

**Figure 2. Genome assembly correlation with genetic maps and Hi-C data.**

**A.** Integrated genetic and physical maps of the cucumber genome assembly. Super-scaffolds of the genome assembly (middle) were anchored to the four linkage groups (left and right): map.1 (green) [3], map.2 (orange) [20], map.3 (light blue) [19], map.4 (pink) [18].

**B.** Heat map of Hi-C contact information. The colors of pixels represent different normalized count of Hi-C links between 30 kb non-overlapping windows for all seven chromosomes on a logarithmic scale.

**Figure 3. Novel repetitive sequences and genes in assembly V3.0.** A. Size of various types of repetitive sequences in the V2.0 and V3.0 assembly. DNA: DNA transposons; LINE: Long Interspersed Nuclear Elements; SINE: Short Interspersed Nuclear Elements; LTRc: Copia Long Terminal Repeat retrotransposons; LTRg: Gypsy Long Terminal Repeat retrotransposons; LTRo: Other LTR categories; Unknown: unknown type. B. The number of full-length LTRs (FL-LTRs) in V2.0 and V3.0. C. A newly predicted full-length LTR in V3.0. TSP: Target Site Repeat; PBS: Primer Binding Site; PPT: Primer Polypurine Tract; IN: Integrase; RT: Reverse Transcriptase. D. An example showing the newly assembled multiple tyrosylprotein sulfotransferase (TPST) genes in V3.0. b'-e' are all TPST genes, these four genes correspond to CsaV3_1G013960, CsaV3_1G013970, CsaV3_1G013980 and CsaV3_1G013990.

**Figure 4. Distribution of the GC content for the whole genome and novel sequences in V3.0.**

**Reference**


1. Woycicki R, Witkowicz J, Gawronski P, Dabrowska J, Lomsadze A, Pawelkowicz M, et al. The genome sequence of the North-European cucumber (*Cucumis sativus* L.) unravels evolutionary adaptation mechanisms in plants. PLoS One. 2011;6:e22728.
2. Li Z, Zhang Z, Yan P, Huang S, Fei Z and Lin K. RNA-Seq improves annotation of protein-coding genes in the cucumber genome. BMC Genomics. 2011;12:540.
3. Yang L, Koo D, Li Y, Zhang X, Luan F, Havey M, et al. Chromosome rearrangements during domestication of cucumber as revealed by high-density genetic mapping and draft genome assembly. Plant J. 2012;71:895-906.

4. Huang S, Li R, Zhang Z, Li L, Gu X, Fan W, et al. The genome of the cucumber, *Cucumis sativus* L. Nat Genet. 2009;41:1275-81.

5. Qi J, Liu X, Shen D, Miao H, Xie B, Li X, et al. A genomic variation map provides insights into the genetic basis of cucumber domestication and diversity. Nat Genet. 2013;45:1510-5.

6. Maumus F and Quesneville H. Impact and insights from ancient repetitive elements in plant genomes. Curr Opin Plant Biol. 2016;30:41-6.

7. Bickhart DM, Rosen BD, Koren S, Sayre BL, Hastie AR, Chan S, et al. Single-molecule sequencing and chromatin conformation capture enable de novo reference assembly of the domestic goat genome. Nat Genet. 2017;49:643-50.

8. Daccord N, Celton JM, Linsmith G, Becker C, Choisne N, Schijlen E, et al. High-quality de novo assembly of the apple genome and methylome dynamics of early fruit development. Nat Genet. 2017;49:1099-106.

9. Du H, Yu Y, Ma Y, Gao Q, Cao Y, Chen Z, et al. Sequencing and de novo assembly of a near complete indica rice genome. Nat Commun. 2017;8:15324.

10. Gordon D, Huddleston J, Chaisson MJ, Hill CM, Kronenberg ZN, Munson KM, et al. Long-read sequence assembly of the gorilla genome. Science. 2016;352:aae0344.

11. Jiao WB, Accinelli GG, Hartwig B, Kiefer C, Baker D, Severing E, et al. Improving and correcting the contiguity of long-read genome assemblies of three plant species using optical mapping and chromosome conformation capture data. Genome Research. 2017;27:778-86.

12. Jiao Y, Peluso P, Shi J, Liang T, Stitzer MC, Wang B, et al. Improved maize reference genome with single-molecule technologies. Nature. 2017;546:524-7.

13. Dudchenko O, Batra SS, Omer AD, Nyquist SK, Hoeger M, Durand NC, et al. *De novo* assembly of the Aedes aegypti genome using Hi-C yields chromosome-length scaffolds. Science. 2017;356:92-5.

14. Yeo S, Coombe L, Warren RL, Chu J and Birol I. ARCS: scaffolding genome drafts with linked reads. Bioinformatics. 2018;34:725-31.

15. Zhang G, Liu K, Li Z, Lohaus R, Hsiao Y, Niu S, et al. The Apostasia genome and the evolution of orchids. Nature. 2017;549:379-83.

16. Lam KK, LaButti K, Khalak A and Tse D. FinisherSC: a repeat-aware tool for upgrading de novo assembly using long reads. Bioinformatics. 2015;31:3207-9.

17. Walker BJ, Abeel T, Shea T, Priest M, Abouelliel A, Sakthikumar S, et al. Pilon: an integrated tool for comprehensive microbial variant detection and genome assembly improvement. PLoS One. 2014;9:e112963.

18. Ren Y, Zhang Z, Liu J, Staub JE, Han Y, Cheng Z, et al. An integrated genetic and cytogenetic map of the cucumber

genome. PLoS One. 2009;4:e5795.

19. Zhang W, Pan J, He H, Zhang C, Li Z, Zhao J, et al. Construction of a high density integrated genetic map for cucumber (*Cucumis sativus* L.). Theor Appl Genet. 2012;124:249-59.

20. Zhou Q, Miao H, Li S, Zhang S, Wang Y, Weng Y, et al. A Sequencing-Based Linkage Map of Cucumber. Mol Plant. 2015;8:961-3.

21. Sun J, Zhang Z, Zong X, Huang S, Li Z and Han Y. A high-resolution cucumber cytogenetic map integrated with the genome assembly. BMC Genomics. 2013;14:461.

22. Ganal. M, Riede. I and Hemleben. V. Organization and sequence analysis of two related satellite DNAs in cucumber (*Cucumis sativus* L.). Journal of Molecular Evolution. 1986;23:23-30.

23. Ganal. M and Hemleben. V. Insertion and amplification of a DNA sequence in satellite DNA of *Cucumis sativus* L. (cucumber). Theor Appl Genet. 1988;75:357-61.

24. Han Y, Zhang Z, Liu J, Lu J, Huang S and Jin W. Distribution of the tandem repeat sequences and karyotyping in cucumber (*Cucumis sativus* L.) by fluorescence in situ hybridization. Cytogenet Genome Res. 2008;122:80-8.

25. Haas BJ, Salzberg SL, Zhu W, Pertea M, Allen JE, Orvis J, et al. Automated eukaryotic gene structure annotation using EVidenceModeler and the Program to Assemble Spliced Alignments. Genome Biol. 2008;9:R7.

26. Murray MG and Thompson WF. Rapid isolation of high molecular weight plant DNA. Nucleic Acids Res. 1980;8:4321-6.

27. Belton JM, McCord RP, Gibcus JH, Naumova N, Zhan Y and Dekker J. Hi-C: a comprehensive technique to capture the conformation of genomes. Methods. 2012;58:268-76.

28. Wingett S, Ewels P, Furlan-Magaril M, Nagano T, Schoenfelder S, Fraser P, et al. HiCUP: pipeline for mapping and processing Hi-C data. F1000Res. 2015;4:1310.

29. Raymond O, Gouzy J, Just J, Badouin H, Verdenaud M, Lemainque A, et al. The Rosa genome provides new insights into the domestication of modern roses. Nat Genet. 2018;50:772-7.

30. Koren S, Walenz BP, Berlin K, Miller JR, Bergman NH and Phillippy AM. Canu: scalable and accurate long-read assembly via adaptive k-mer weighting and repeat separation. Genome Res. 2017;27:722-36.

31. Altschul SF, Gish W, Miller W, Myers EW and Lipman DJ. Basic local alignment search tool. J Mol Biol. 1990;215:403-10.

32. Kurtz S, Phillippy A, Delcher AL, Smoot M, Shumway M, Antonescu C, et al. Versatile and open software for comparing large genomes. Genome Biol. 2004;5:R12.

33. Tang H, Zhang X, Miao C, Zhang J, Ming R, Schnable JC, et al. ALLMAPS: robust scaffold ordering based on

multiple maps. Genome Biol. 2015;16:3.

34. Coombe L, Zhang J, Vandervalk BP, Chu J, Jackman SD, Birol I, et al. ARKS: chromosome-scale scaffolding of human genome drafts with linked read kmers. BMC Bioinformatics. 2018;19:234.

35. Xu Z and Wang H. LTR_FINDER: an efficient tool for the prediction of full-length LTR retrotransposons. Nucleic Acids Res. 2007;35:W265-8.

36. Edgar RC. MUSCLE: a multiple sequence alignment method with reduced time and space complexity. BMC Bioinformatics. 2004;5:113.

37. Rice P, Longden I and Bleasby A. EMBOSS: the European Molecular Biology Open Software Suite. Trends Genet. 2000;16:276-7.

38. Nystedt B, Street NR, Wetterbom A, Zuccolo A, Lin YC, Scofield DG, et al. The Norway spruce genome sequence and conifer genome evolution. Nature. 2013;497:579-84.

39. Majoros WH, Pertea M and Salzberg SL. TigrScan and GlimmerHMM: two open source ab initio eukaryotic gene-finders. Bioinformatics. 2004;20:2878-9.

40. Johnson AD, Handsaker RE, Pulit SL, Nizzari MM, O'Donnell CJ and de Bakker PI. SNAP: a web-based tool for identification and annotation of proxy SNPs using HapMap. Bioinformatics. 2008;24:2938-9.

41. Wei G, Tian P, Zhang F, Qin H, Miao H, Chen Q, et al. Integrative Analyses of Nontargeted Volatile Profiling and Transcriptome Data Provide Molecular Insight into VOC Diversity in Cucumber Plants (*Cucumis sativus*). Plant Physiol. 2016;172:603-18.

42. Qiu L, Jiang B, Fang J, Shen Y, Fang Z, Rm SK, et al. Analysis of transcriptome in hickory (*Carya cathayensis*), and uncover the dynamics in the hormonal signaling pathway during graft process. BMC Genomics. 2016;17:935.

43. Iwata H and Gotoh O. Benchmarking spliced alignment programs including Spaln2, an extended version of Spaln that incorporates additional species-specific features. Nucleic Acids Res. 2012;40:e161.

44. Li H and Durbin R. Fast and accurate short read alignment with Burrows-Wheeler transform. Bioinformatics. 2009;25:1754-60.

45. Trapnell C, Pachter L and Salzberg SL. TopHat: discovering splice junctions with RNA-Seq. Bioinformatics. 2009;25:1105-11.

46. Trapnell C, Williams BA, Pertea G, Mortazavi A, Kwan G, van Baren MJ, et al. Transcript assembly and quantification by RNA-Seq reveals unannotated transcripts and isoform switching during cell differentiation. Nat Biotechnol. 2010;28:511-5.

47. Li L, Stoeckert CJ, Jr. and Roos DS. OrthoMCL: identification of ortholog groups for eukaryotic genomes. Genome

Res. 2003;13:2178-89.

Figure1

Click here to access/download;Figure;Figure1.pdf

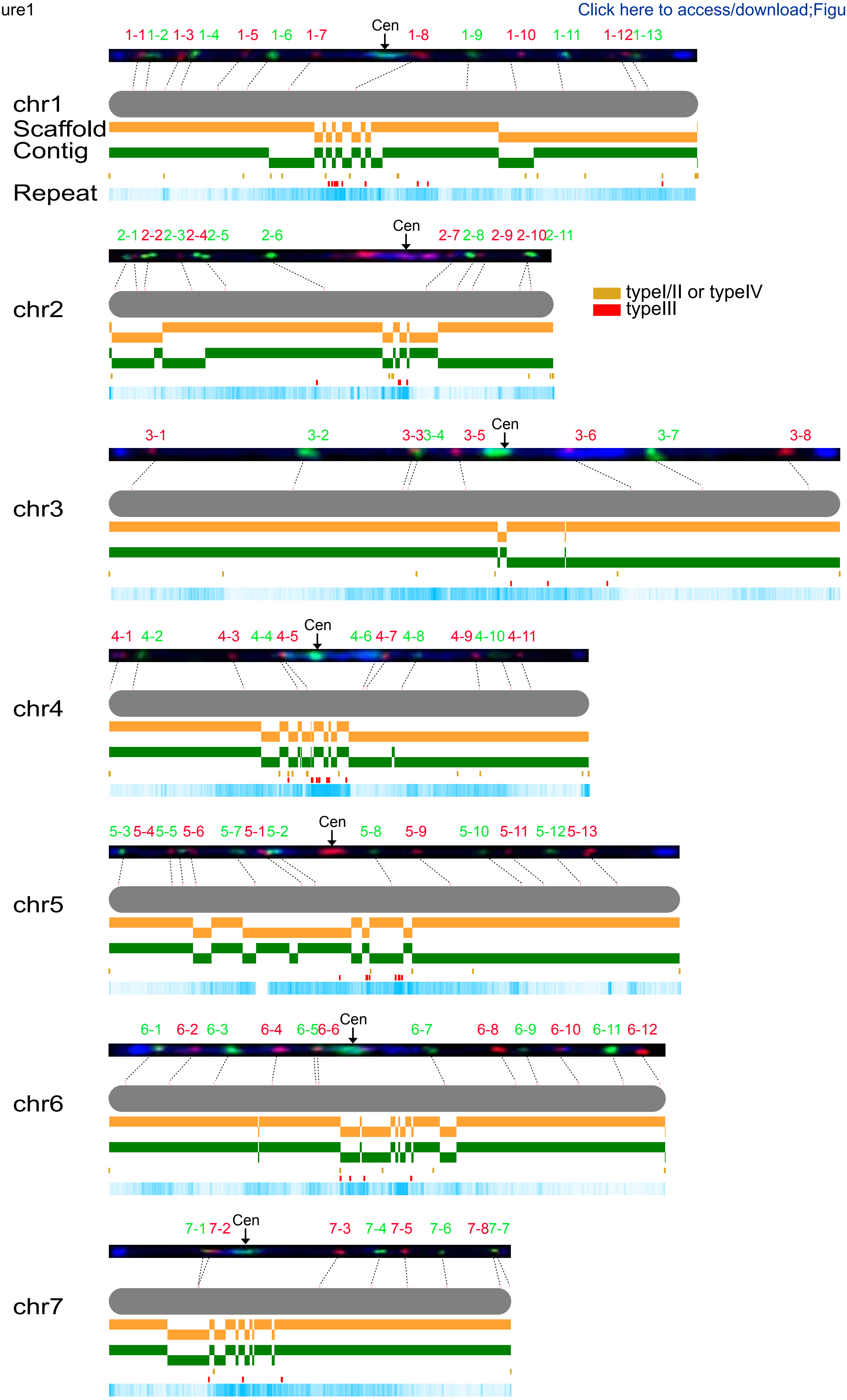

Click here to access/download;Figure;Figure2.pdf

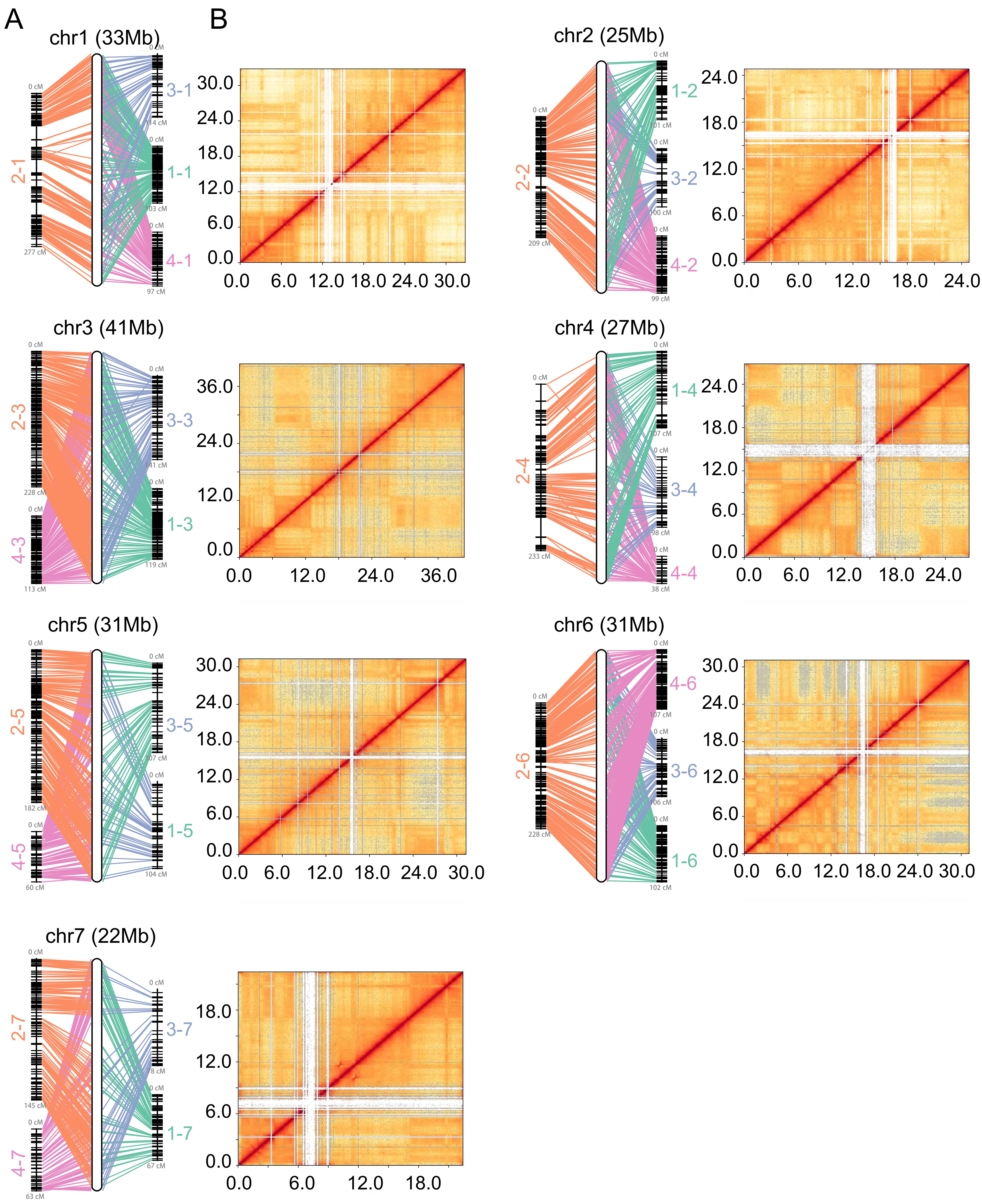

Figure3

Click here to access/download;Figure;Figure3.pdf

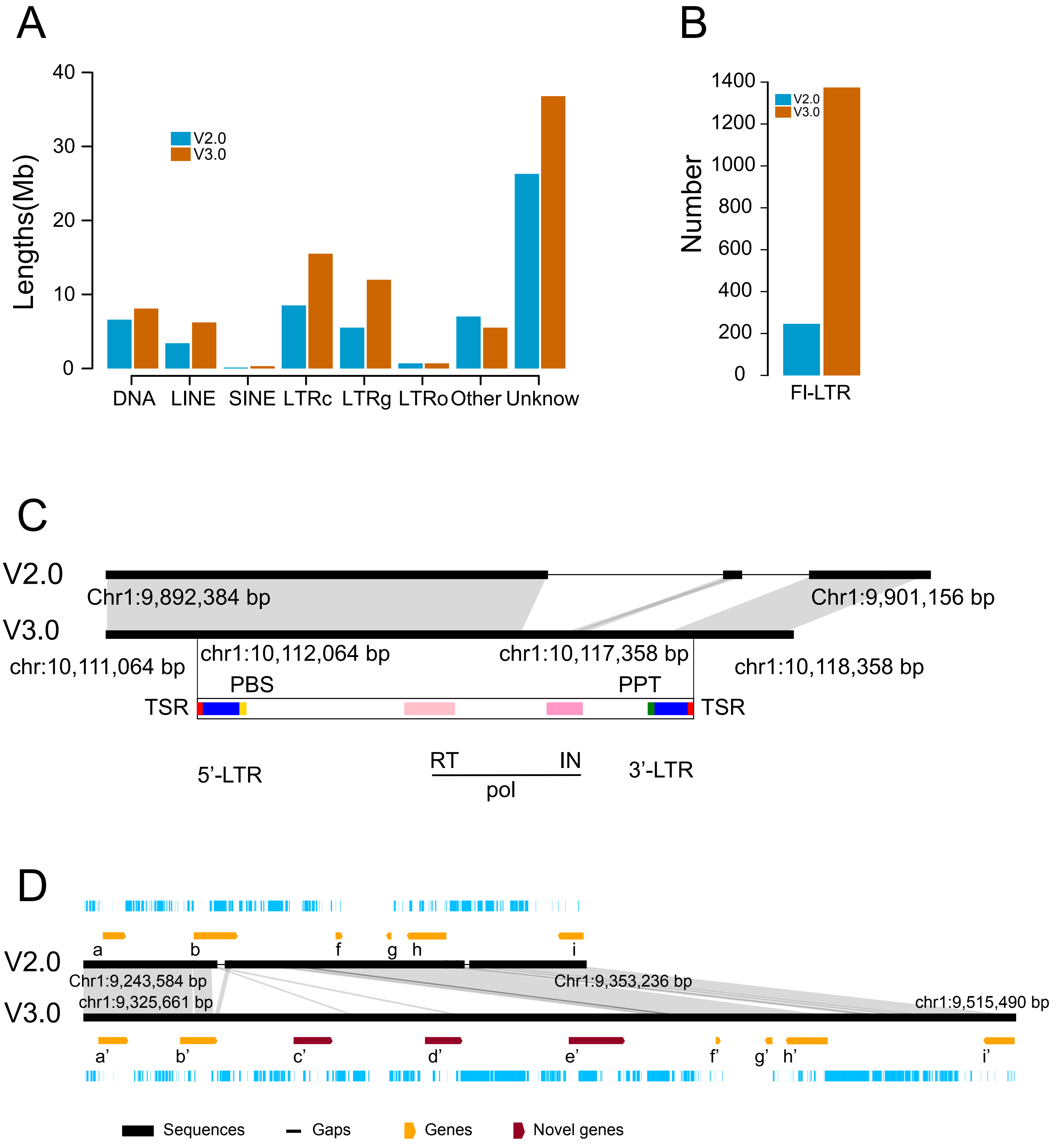

Figure4

Click here to access/download;Figure;Figure4.

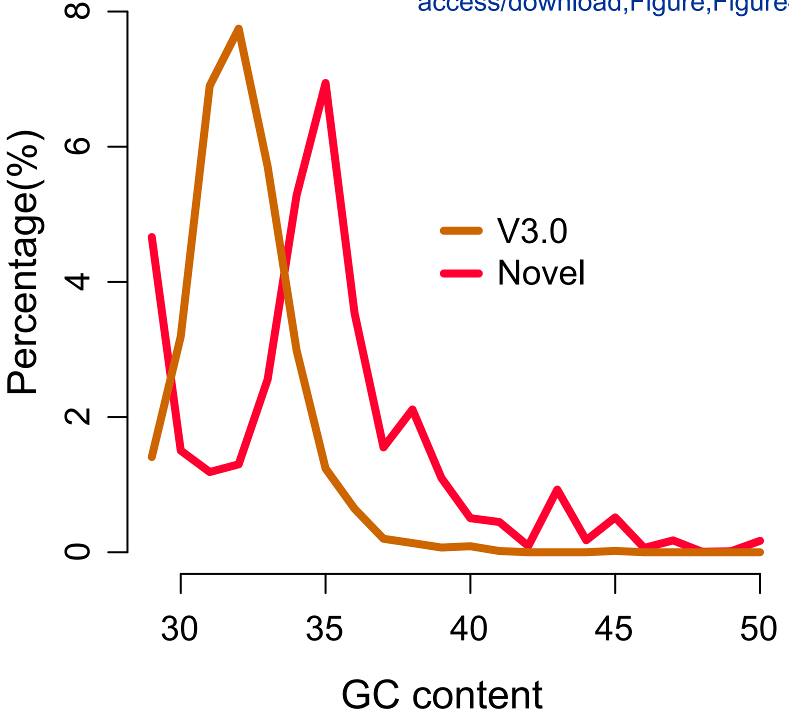

Click here to access/download
**Supplementary Material**
Supplemental_Gigascience.docx

Table1

Click here to access/download
**Supplementary Material**
Supplemetal S6.xlsx

Click here to access/download
**Supplementary Material**
Supplemental Table S7.xlsx

Click here to access/download
**Supplementary Material**
To Reviewer_GigaScience.docx

Personal Cover Click here to access/download;Personal Cover;cucumber V3 CV 20190313.docx

Dear Dr. Hongling Zhou

We greatly appreciate the dedication of the reviewers and the editor to help us to improve the manuscript. All the data and custom scripts mentioned in the MS are available without restriction. According to the reviewer's comments and suggestions, we have revised our manuscript and provide a point-by-point response to the editor/reviewers' concerns. Within the 'Response to Reviewers' box, only text message can be added, the corresponding figures can not be showed. To provide more evidence and data to reviewers, we used several figures in the point-by-point response to the reviewers' concerns. In addition to the text within 'Response to Reviewers' box, the file containing the needed figures was submitted as a supplementary file (To Reviewer_GigaScience.docx).

Thank you in advance for considering this work.

Sincerely yours,

Zhonghua Zhang
Institute of Vegetables and Flowers, Chinese Academy of Agricultural Sciences
No. 12, Zhong Guan Cun Nan Da Jie, Beijing, 100081, China
Tel: +86-10-62117612
Mobile Phone: +8613699205910
Email: zhangzhonghua@caas.cn
