# Supplementary material for: A chromosome-scale genome assembly of cucumber (Cucumis sativus L.)
Source: Gigascience. 2019 Jun 18;8(6):giz072. doi: 10.1093/gigascience/giz072 (PMC6582320; doi:10.1093/gigascience/giz072)
Supplement: giz072_GIGA-D-18-00507_Revision_2 [file giz072_giga-d-18-00507_revision_2.pdf]

GigaScience

A chromosome-scale genome assembly of cucumber (Cucumis sativus L.)

--Manuscript Draft--

**Manuscript Number:**	GIGA-D-18-00507R2
**Full Title:**	A chromosome-scale genome assembly of cucumber (Cucumis sativus L.)
**Article Type:**	Data Note
**Funding Information:**	China National Key Research and Development Program for Crop Breeding (2016YFD0100307) — Prof. Zhonghua Zhang<br>National Natural Science Foundation of China (31322047,31772304) — Prof. Zhonghua Zhang<br>National Youth Top-notch Talent Support Program in China (None) — Prof. Zhonghua Zhang<br>Science and Technology Innovation Program of Chinese Academy of Agricultural Science (None) — Prof. Zhonghua Zhang
**Abstract:**	Background: Accurate and complete reference genome assemblies are fundamental for biological research. Cucumber is an important vegetable crop and model system for sex determination and vascular biology. Low coverage Sanger sequences and high coverage short Illumina sequences have been used to assemble draft cucumber genomes, but the incompleteness and low quality of these genomes limit their use in comparative genomics and genetic research. A high-quality and complete cucumber genome assembly is therefore essential.<br><br>Findings: We assembled single-molecule real-time (SMRT) long reads to generate a much improved cucumber reference genome. This version contains 174 contigs with a total length of 226.2 Mb and an N50 of 8.9 Mb, and provides 29.0 Mb more sequence data than previous versions. Using 10X Genomics and high-throughput chromosome conformation capture (Hi-C) data, 89 contigs (~211.0 Mb) were directly linked into seven pseudo-chromosome sequences. The newly assembled regions show much higher GC or AT content than found previously, which is likely to have been inaccessible to Illumina sequencing. The new assembly contains 1374 full-length long terminal retrotransposons (LTRs), and 1,078 novel genes including 239 tandemly duplicated genes. For example, we found four tandemly duplicated tyrosylprotein sulfotransferases, not a single copy of the gene as was found previously and in most other plants.<br>Conclusion: This high-quality genome presents novel features of the cucumber genome, and will serve as a valuable resource for genetic research in cucumber and plant comparative genomics.
**Corresponding Author:**	Zhonghua Zhang<br><br>CHINA
**Corresponding Author Secondary Information:**	
**Corresponding Author's Institution:**	
**Corresponding Author's Secondary Institution:**	
**First Author:**	Qing Li
**First Author Secondary Information:**	
**Order of Authors:**	Qing Li<br>Hongbo Li<br>Wu Huang

Powered by Editorial Manager® and ProduXion Manager® from Aries Systems Corporation

	Yuanchao Xu
	Qian Zhou
	Shenhao Wang
	Jue Ruan
	Sanwen Huang
	Zhonghua Zhang
**Order of Authors Secondary Information:**	
**Response to Reviewers:**	We greatly appreciate the dedication of the reviewers and the editor to help us to improve the manuscript. According to the reviewer and editor's comments and suggestions, we have revised our manuscript and upload them again.
**Additional Information:**	
**Question**	**Response**
Are you submitting this manuscript to a special series or article collection?	No
**Experimental design and statistics**<br><br>Full details of the experimental design and statistical methods used should be given in the Methods section, as detailed in our Minimum Standards Reporting Checklist. Information essential to interpreting the data presented should be made available in the figure legends.<br><br>Have you included all the information requested in your manuscript?	Yes
**Resources**<br><br>A description of all resources used, including antibodies, cell lines, animals and software tools, with enough information to allow them to be uniquely identified, should be included in the Methods section. Authors are strongly encouraged to cite Research Resource Identifiers (RRIDs) for antibodies, model organisms and tools, where possible.<br><br>Have you included the information requested as detailed in our Minimum Standards Reporting Checklist?	Yes
**Availability of data and materials**	Yes

All datasets and code on which the conclusions of the paper rely must be either included in your submission or deposited in publicly available repositories (where available and ethically appropriate), referencing such data using a unique identifier in the references and in the "Availability of Data and Materials" section of your manuscript.<br><br>Have you have met the above requirement as detailed in our Minimum Standards Reporting Checklist?	

Powered by Editorial Manager® and ProduXion Manager® from Aries Systems Corporation

Manuscript
Click here to access/download;Manuscript;507-cucumber_paper_edit-0520.docx
Click here to view linked References

A chromosome-scale genome assembly of cucumber (*Cucumis sativus* L.)

Qing Li[1,#], Hongbo Li[1,#], Wu Huang[1,2,#], Yuanchao Xu[1], Qian Zhou[1,2], Shenhao Wang[3], Jue Ruan[2], Sanwen Huang[2], Zhonghua Zhang[1,*]

[1]Institute of Vegetables and Flowers, Chinese Academy of Agricultural Sciences, No.12, Haidian District, Beijing 100081, China

[2]Agricultural Genomics Institute at Shenzhen, Chinese Academy of Agricultural Sciences, No. 7, Pengfei Road, Dapeng District, Shenzhen 518124, China

[3]College of Horticulture, Northwest A&F University, Yangling, Shanxi 712100, China

[#] These authors contributed equally to this work.

*Correspondence address: Zhonghua Zhang, Institute of Vegetables and Flowers, Chinese Academy of Agricultural Sciences, No.12 Zhongguancun South St., Haidian District Beijing 10081, P.R. China; Tel: +86-10-62117612 or +86-13-699205910; Email: zhangzhonghua@caas.cn

ORCIDs

Qing Li: 0000-0002-2397-5922; Hongbo Li: 0000-0003-1579-4600; Wu Huang: 0000-0002-5015-7167; Shenhao Wang: 0000-0003-1422-020X; Jue Ruan: 0000-0003-3713-3192; Sanwen Huang: 0000-0002-8547-5309; Zhonghua Zhang: 0000-0002-1034-227X

Abstract

Background: Accurate and complete reference genome assemblies are fundamental for biological research. Cucumber is an important vegetable crop and model system for sex determination and vascular biology. Low coverage Sanger sequences and high coverage short Illumina sequences have been used to assemble draft cucumber genomes, but the incompleteness and low quality of these genomes limit their use in comparative genomics and genetic research. A high-quality and complete cucumber genome assembly is therefore essential.

Findings: We assembled single-molecule real-time (SMRT) long reads to generate a much improved cucumber reference genome. This version contains 174 contigs with a total length of 226.2 Mb and an N50 of 8.9 Mb, and provides 29.0 Mb more sequence data than previous versions. Using 10X Genomics and high-throughput chromosome conformation capture (Hi-C) data, 89 contigs (~211.0 Mb) were directly linked into seven pseudo-chromosome sequences. The newly assembled regions show much higher GC or AT content than found previously, which is likely to have been inaccessible to Illumina sequencing. The new assembly contains 1374 full-length long terminal retrotransposons (LTRs), and 1,078 novel genes including 239 tandemly duplicated genes. For example, we found four tandemly duplicated tyrosylprotein sulfotransferases, not a single copy of the gene as was found previously and in most other plants.

Conclusion: This high-quality genome presents novel features of the cucumber genome, and will serve as a valuable resource for genetic research in cucumber and plant comparative genomics.

Keywords: Cucumber; PacBio; Hi-C; Genomics; Chromosome-scale assembly

Background

Accurate and complete reference genome assembly is essential for genetic and genome-wide studies of individual and multiple species. Cucumber (*Cucumis sativus* L., NCBI: txid3659), is an important vegetable crop and a model plant for sex determination and vascular biology. Four genome assemblies of cucumber, including one wild and three cultivated accessions, have been released since 2009 [1-6], and were mainly assembled using Illumina short sequences. Compared with the estimated genome size of 350 Mb [4, 5], these assemblies range between 197 and 203 Mb in length; therefore approximately 150 Mb of sequence data is still missing. Cytogenetic and sequence information suggests that about 100 Mb of satellite sequences, which comprise very large arrays of tandemly repeated DNAs with lengths of 177 or 366 bp, are present in cucumber centromeric/telomeric regions, and these cannot be assembled using current sequencing technology. Current assemblies also have lots of other missing sequences, and this will hamper genetic-based gene isolation, the identification of variations and epigenetic modification sites, and comparative analyses at the population level and across closely related species. The contig and scaffold N50 sizes of the released cucumber genome assembly (version 2.0) are only 30.0 kb and 1.4 Mb, respectively [2], leaving more than 10,000 gaps. Missing sequences and low contiguity

limit the applications of this genome assembly in comparative genomics and genetic research. Therefore, a high-quality and complete cucumber genome assembly is essential.

Repetitive sequences such as transposable elements pose the largest challenge for generating a high-quality genome assembly, especially for plant genomes [7]. The nature of short reads generated by Illumina sequencing technology means that similar repetitive sequences are often collapsed into a single copy. To overcome this limitation, the development of single-molecule real-time (SMRT) sequencing technologies such as Pacific Biosciences (PacBio) and Oxford Nanopore, which generate long reads of more than 10 kb in size, has advanced in recent years. High-quality genome assemblies for several plants and animals have been generated using these technologies [8-13]. Repetitive sequences in cucumber are estimated to account for 30% of the genome [4], so it is necessary to improve the currently available assembly using long-read sequencing technology.

Scaffolding technologies are critical to accurately order and orient assembled contigs. In past decades, read information from a variety of mate-pair libraries with different insert sizes has been widely used for scaffolding. However, preparing mate-pair library is expensive, and the read information is sometimes also confused by repetitive elements. In recent years, new cost-effective

and accurate technologies, including 10X Genomics, optical mapping and high-throughput chromosome conformation capture (Hi-C), have been developed. These can aid scaffolding by providing long-range contiguity information ranging from ~50 kb to several megabases [6, 12, 14-16]. These new technologies will greatly benefit the contiguity of the cucumber genome assembly.

Data Description

Here, we describe the assembly of an improved reference genome assembly for cucumber by combining the read sequence data from PacBio, 10X Genomics and Hi-C technologies. Comparing the new assembly to the previously released version revealed much improvement in terms of genome completeness and contiguity. This work also presents numerous novel sequences, such as protein-coding genes and intact retrotransposons, thus provides a robust reference sequence for cucumber genetics.

Genome sequencing and assembly

The genome of the 'Chinese long' inbred line 9930 was assembled several years ago based on Illumina and Sanger sequences [2, 4, 5]. We sequenced this same line using newer technologies; specifically, PacBio, 10X Genomics, and Hi-C. A total of 16.2 Gb PacBio read sequences, representing 46.2-fold genome coverage with a sub-read N50 length of 10.8 kb were generated (Additional File 1). To fully utilize the PacBio data, meta-assembly was performed based on two CANU pre-assemblies and four FALCON pre-assemblies, resulting in 195 contigs spanning 232.3 Mb in length. Comparing the final assembly with the pre-assemblies showed the complementarity of the six initial assemblies (Additional file 2). Assembled contigs containing potential bacteria and plastid contamination were eliminated. Using FinisherSC [17], we aligned raw PacBio reads to the resulting contigs, merged any contigs that could be connected, and the gaps were filled by reads. Illumina sequences were mapped to the assembled sequences to correct any potential sequencing errors (Additional file 3). A total of 49,157 single base pair substitutions and 156,931 small insertion/deletions (indels) were corrected using Pilon [18]. Using four genetic maps [3, 19-21], obvious assembly errors were detected, and these contigs were split. All contigs were aligned against the previous assembly (version 2.0), and no obvious errors were observed.

Finally, a total of 174 contigs were obtained with a total length of 226.2 Mb and an N50 length of 8.9 Mb (Additional file 4). This represents an approximately 234.8-fold improvement in contiguity than the previous assembly.

To build scaffolds, we generated 20.2 Gb linked reads with long-range information of 50 kb DNA fragments using the 10X Genomics platform, and 68.5 Gb long-range contact reads from Hi-C (Additional file 1). Linked reads connected 174 contigs into 157 scaffolds, resulting in an N50 length of 11.5 Mb. On the basis of these scaffolds, we further linked them into 85 super-scaffolds with an N50 of 31.1 Mb using Hi-C data (Additional file 4). Among these super-scaffolds, seven with a total length of 211.0 Mb corresponded directly to the seven cucumber chromosomes, thus providing an additional 19.1 Mb of sequence data for the seven pseudo-chromosome sequences (Figure 1) relative to the genome v2.0. Lacking Hi-C contact information means that the remaining 78 super-scaffolds (15.2 Mb) cannot be clustered into any of the seven chromosomes, suggesting that these could be mainly covered by repetitive sequences. Therefore, we present here more complete pseudo-chromosome sequences for the cucumber reference genome.

Evaluation of the genome quality

To assess the quality of the new genome assembly (v3.0), we mapped 6.0 Gb new Illumina and previous Sanger reads (Additional file 3) to the final assembled sequences. Only 53,179 substitutions and 30,546 small indels were identified as homozygous variations (index >0.9). Thus, the error rates for single base pair and small indels are estimated to be below 0.00024 and 0.00014, respectively, which indicates that v3.0 has high accuracy at the single base-pair level.

The genome sequences are highly consistent with genetic maps and Hi-C data, which show the high accuracy of contiguity for the assembly (Figure 2). The orders of genetic markers are consistent with the assembly sequences, with a correlation coefficient of 0.98 on average. From the long-range Hi-C contact information, we can see that most regions show close contacts with nearby sequences, and only the centromeric/telomeric regions have few contacts with other genomic segments.

Integrating the genome assembly with the cytogenetic map [22] reveals the high level of completeness of v3.0 (Figure 1). Most of the centromeric and telomeric sequences are absent from each of the seven chromosomes. The main components of the centromere are satellite type III, and

these are detected at the ends of the super-scaffolds around the centromeres, indicating their boundaries. Among the 14 ends of the seven chromosomes, 13 have satellite type I/II/IV components; this constitutes the majority of the telomere, indicating the telomeric boundary. This assembly comprises almost all the genome sequences except for the centromeric and telemetric regions, which are largely made up of satellite sequences and account for ~30% (~105 Mb) of total nuclear DNA [23, 24], thus cannot be assembled using current sequencing technologies [25].

We also explored the consistency between v2.0 and v3.0 genomes using whole-genome alignment (Additional file 5). Many novel sequences appear to be inserted into genome v3.0. The distal sequences on chromosome 5 of v2.0 are translocated to the correct position in v3.0, which is consistent with a previous report [21, 22]. In addition, two inversions on chromosomes 4 and 6, which constitute assembly errors in v2.0, were corrected in v3.0. This is supported by the data in the Hi-C heat map (Figure 2).

To assess the completeness of gene space, we downloaded 121.7 Gb of RNA-seq sequences generated from 39 samples (Additional file 6), including a variety of tissues such as root, stem, leaf, flower, and fruit, and mapped them to assemblies v2.0 and v3.0, respectively. Compared with

v2.0, 3.2 Gb additional RNA-seq sequences were mapped in v3.0, resulting in 932.2 kb additional expressed genomic regions. This new assembly therefore represents a higher completeness in terms of gene space.

Genome annotation reveals novel repetitive sequences and genes

In v3.0, we identified 82.0 Mb of repetitive sequences, representing 36.43% of the genome (Additional file 7). This is approximately 27.6 Mb more than was predicted in v2.0 (54.4 Mb). Among these repetitive sequences, long terminal retrotransposons (LTRs) are the most abundant, and their sizes were markedly increased in v3.0 (Figure 3A). A total of 1374 full-length LTRs (FL-LTRs) were predicted in v3.0; five times more (267) than in v2.0 (Figure 3B). Most of these FL-LTRs were partially assembled in v2.0, thus they were not annotated as FL-LTRs. For example, a FL-LTR on chromosome 1 was not predicted because of the absence of pol-domain and long terminal repeats in v2.0 (Figure 3C). Insert time analysis of these FL-LTRs reveals that most of them occurred recently in cucumber, explaining the complexity of these regions during the assembly process (Additional file 8) [7].

In v3.0, 24,317 protein-coding genes were predicted by combining three methods: *ab initio*, protein homology-based, and transcriptome sequences, using the EVidenceModeler pipeline [26]. Compared with the predicted genes in v2.0, 1078 genes (Additional file 9) were newly assembled in v3.0, and 2693 were newly predicted in v3.0 but were not predicted in v2.0 because of sequencing gaps, errors or annotation pipeline bias. Of the newly assembled genes, 931 are expressed in at least one of the above 39 samples with RNA-seq data, indicating their high reliability. Compared with all genes, these genes are characterized by short average length and lower average exon number (Additional file 10). Based on the alignments of genes in v3.0 and v2.0, we also identified 1970 fragmented genes in V2.0 that correspond to 932 genes in v3.0. Conversely, 687 genes in v2.0 were split into 337 in v3.0 (Additional file 11). Along with the pseudo-chromosomes, distribution of 1078 novel genes in v3.0 indicates that 239 are tandemly duplicated genes. For example, in v2.0, only one tyrosylprotein sulfotransferase (TPST), was predicted. In most plants, including Arabidopsis and tomato, this is a single copy gene; however, in cucumber v3.0, four tandemly duplicated genes were obtained (Figure 3D). Two predicted TPSTs in the wild cucumber genome [6] also support the presence of multiple TPSTs in the

cucumber genome [4]. Therefore, the new genome provides a more complete gene set for functional genomic research in cucumber.

Features of novel sequences in assembly v3.0

To explore the features of the novel sequences in the new assembly, we analyzed them using Illumina reads and the newly assembled genes. At the whole genome level, sequences with a GC content of approximately 32.8% were dominantly abundant; however, the distribution of GC content among novel sequences peaked at approximately 35.0% (Figure 4). The newly assembled genes also show a similar GC distribution (Additional file 12). This suggests that sequences with abnormal GC content could be only generated using the PacBio sequencing technology. Among the new genes, more than 30 domains, including pectinesterase inhibitor (IPR034086, pectinesterase inhibitor, plant; IPR006501, pectinesterase inhibitor domain, etc.), zinc finger, and CCHC-type domain (IPR036875) were significantly enriched ($P<0.005$) (Additional file 13). These results indicate that the PacBio sequencing technology is advantageous for some types of genes.

Conclusion

By combining long-read sequences generated by PacBio, long-range information generated by 10X Genomics, and long-range Hi-C contact reads, we provide a high quality cucumber reference genome for the community. Many repetitive sequences and genes have been identified and added to the assembly, especially sequences with high GC or high AT content, and genes with certain domains. More tandemly duplicated genes were assembled in the new genome. These data provide a valuable resource for comparative genomics, epigenetics, gene isolation, and transposon research.

Methods

PacBio sequencing

High quality genomic DNA was extracted from young leaves of ‘Chinese long’ inbred line 9930 cucumber, using a modified CTAB method [27]. Genomic DNA was sheared to a size range of 15–40 kb using a Megaruptor (Diagenode) device, and was then used for single-molecule real time (SMRT) library preparation as recommended by Pacific Biosciences. Two SMRTbellTM

templates were prepared in 2014 and 2016, respectively. The first library was sequenced on a PacBio RSII platform, generating 1,470,953 reads (11.0 Gb). The second library was sequenced on a PacBio Sequel platform, generating 628,153 reads (5.2 Gb).

10X Genomics linked-read sequencing

A total of 0.3 ng high-molecular-weight DNA was prepared and loaded onto a Chromium Controller chip with 10X Chromium reagents and gel beads, following the recommended protocols [28]. On average, the loaded DNA molecule was ~50 kb in length. There are about 1 million droplets on a Chromium Controller chip. Within each droplet, several DNA molecules were sheared, and the sheared DNA fragments were tagged with the same barcode. Then, all barcoded DNA fragments within these droplets were sequenced on an Illumina Hiseq X Ten sequencer to produce 2 ×150 bp paired-end sequences.

Hi-C read sequencing

Leaves of cucumber line 9930 were fixed with 1% formaldehyde solution, and chromatin was cross-linked and digested using restriction enzyme HindIII. The 5′ overhangs were filled in with biotinylated nucleotides, and free blunt ends were then ligated. After ligation, crosslinks were reversed and the DNA was purified from protein. Purified DNA was treated to remove biotin that was not internal to ligated fragments. The DNA was then sheared into fragment sizes of ~350 bp. Two sequencing libraries were prepared as described previously [29]. The libraries were sequenced on an Illumina HiSeq X Ten platform. For each library, a total of 223 million paired-end reads of 150 bp in length were generated, representing 195.5-fold coverage of the total cucumber genome. A detailed quality control (QC) report for the Hi-C sequencing was yielded by HiCUP (HiCUP, RRID:SCR_005569) [30].

De novo assembly of PacBio reads

Meta-assembly of PacBio reads from SMRT sequencing was performed as previously described [31]. Briefly, meta-assembled contigs were generated using CANU 1.7 (Canu, RRID:SCR_015880) [32] by combining results from two CANU and four FALCON/til-r

assemblies in which the number of contigs ranged from 589–1094 with a contig N50 length between 2.4 Mb and 3.6 Mb (see Additional file 14 for detailed information). Assembled contigs were aligned against bacterial genomes and cucumber plasmid genomes from GenBank using BLAST [33]. If more than 70% of a contig showed >95% identity with a bacterial or plasmid genome, it was eliminated. Using the FinisherSC pipeline [17] with default parameters, contigs that could be connected by raw PacBio reads were determined, and gaps were filled by reads. To increase the accuracy of contig sequences, previously generated Illumina and Sanger reads (Additional file 3) were aligned to the contigs. Potential sequence errors in the form of single base pair substitutions and insertion/deletions (indels) were corrected by running Pilon (Pilon, RRID:SCR_014731) [18] two times, with the parameters: --fix all --chunksize 20000000 --mindepth 0.4 --K 65 --gapmargin 150000 --vcf --changes --tracks --minmq 10. Corrected contigs were also aligned against the previous genome assembly (v2.0) using MUMmer [34] with default parameters, and were anchored onto the seven linkage groups of the four genetic maps [3, 19-21] using ALLMAPS [35]. Conflicting contigs with the orders of molecular markers from the four

genetic maps were manually checked and split using the alignment results against v2.0. Hi-C data were also aligned to the contigs to check and correct misassemblies.

Scaffold construction

The final contigs were connected into scaffolds using 10X linked reads by ARKS [36] with the following parameters: m=20–20000 threads=20 a=0.9. By aligning the sequences of genetic markers and Hi-C data to the assembled scaffolds, scaffolds conflicting with the orders of molecular markers or long-range contact information were split. Then, the chromosome-level super-scaffolds were constructed on the basis of the genome-wide chromatin interaction information using the 3d-dna pipeline [14] with the parameters: -m haploid -i 15000 -r 0. This resulted in seven chromosome-level super-scaffolds, representing seven cucumber pseudo-chromosomes, and 78 short-length super-scaffolds that could not be clustered because they lacked interactions with the seven chromosome-level super-scaffolds.

Pseudo-chromosome construction

The seven chromosome-level super-scaffolds were anchored onto the seven linkage groups of the four genetic maps [3, 19-21], and orientated into the seven pseudo-chromosomes using ALLMAPS [35] with default parameters. The pseudo-chromosomes were further integrated with the cytogenetic map by mapping the marker sequences and satellite sequences (Type I/II/III/IV) onto the assembly using BLASTN (BLASTN, RRID:SCR_001598, v2.2.15) at an e-value cutoff of 0.05. Satellite sequences are abundantly distributed within centromeric and telomeric regions, so the positions of centromeres and telomeres were marked accordingly.

Genome annotation

Repetitive sequences

RepeatModeler (RepeatModeler, RRID:SCR_015027 [37] was used to search *de novo* for repetitive sequences within genome assemblies v3.0 and v2.0. Identified repeats and the TIGR plant repeat database [38] were then used to identify and mask the repeats in v3.0 and v2.0 using RepeatMasker (RepeatMasker, RRID:SCR_012954 [39]. The repeats were classified into different types based on RepeatMasker annotation. Full-length LTR retrotransposons (FL-LTRs) were also

identified using LTR_Finder (LTR_Finder, RRID:SCR_015247, v1.0.6) [40], with the command line 'ltr_finder genome.fa -s tRNAdb/Athal-tRNAs.fa -a ps_scan > result.txt'. The long terminal repeats of FL-LTRs were aligned with MUSCLE (MUSCLE, RRID:SCR_011812) [41], and the nucleotide distance (D) was estimated using the Kimura two-parameter (K2p) (transition–transversion ratio) criterion, as implemented in the distmat program of EMBOSS (EMBOSS, v6.60, RRID:SCR_008493) [42]. The insertion time (T) of an LTR retrotransposon was calculated using the formula: $T= D/2\mu$, where μ = 4.5e-9, and rate of nucleotide substitution (μ) was inferred according to Nystedt's method [43].

Protein-coding genes

Putative protein-coding genes were predicted using EVidenceModeler (EVidenceModeler, RRID:SCR_014659) [26] by integrating several *ab initio* gene predictors, including Augustus (Augustus, RRID:SCR_008417 [44]), GlimmerHMM (GlimmerHMM, RRID:SCR_002654) [45] and SNAP (SNAP, RRID:SCR_007936) [46], as well as RNA-seq data and homologous proteins

from other plant species. A total of 121.7 Gb RNA-seq sequences generated from 39 samples (Additional file 6), including tissues such as root, stem, leaf, flower and fruit [2, 47, 48] were used for gene prediction. In addition, genes in v2.0 that were not predicted in v3.0 were added into the protein-coding gene set using Spaln [49].

Functional annotation of protein-coding genes

All predicted proteins were aligned against proteins found in UniProt [50] and The Arabidopsis Information Resource (TAIR, [51]) databases. Predicted proteins were annotated as the best-matched protein. Functional annotation was also performed using InterProScan (InterProScan, RRID:SCR_005829). Gene Ontology (GO) terms were assigned according to InterPro classification.

Comparative analyses between assemblies v2.0 and v3.0

Evaluating the accuracy of the genome

The accuracy of genome assembly quality was assessed by aligning previous Sanger reads and 6.0 Gb of new Illumina reads to the corrected contigs using BWA (BWA, RRID:SCR_010910) [52]. Genomic variations were called using GenomeAnalysisTK [53] with default parameters. Considering that the sequenced cucumber 9930 is a highly inbred line, we expected a very low heterozygous rate: index>0.9.

Whole-genome alignment

Whole-genome alignment of v3.0 and v2.0 genomes were conducted (Additional file 5) using the nucmer program within MUMmer software (version 4.0.0beta2) [34], with parameters “-l 100 -c 100”. Then, show-coords was used to show and filter nucmer results with the parameters: “o -l -r -I 99 -L 1000”. The figure (Additional file 5) was plotted using the Python package svgwrite [54].

Mapping RNA-seq data

All downloaded RNA-seq reads were mapped to genome assemblies v2.0 and v3.0 using TopHat 2.1.1 (TopHat, RRID:SCR_013035) with default parameters [55]. On the basis of the alignments, transcripts were assembled using Cufflinks 2.2.1 (Cufflinks, RRID:SCR_014597) without genome guidance [56].

Identification of novel genes in v3.0

Coding sequences of predicted genes in v3.0 were aligned against those in v2.0 using BLAST and *vice versa*. Combined with syntenic information, gene pairs were determined based on the alignments. The corresponding genes were classified as one-to-one, one-to-multiple, or multiple-to-multiple using Python scripts. For the remaining genes in v3.0, gene sequences, including introns, were aligned against the v2.0 genome. If the matched region did not meet the threshold of coverage >50% and identity >95%, then the query gene was considered to be novel in v3.0. Otherwise, the sequences of matched regions in v2.0 were extracted and then aligned against the v3.0 genome. If the matched region in v3.0 covered the whole query gene and the identity was

more than 95%, we considered the v3.0 query gene to have an unpredicted counterpart in v2.0. Genes not classified above were also considered to be novel genes.

GC content

Genome sequences were split into multiple non-overlapping 100 kb windows. For each window, the GC content was calculated using a Python script. For the novel sequences in v3.0, the GC content of each DNA fragment was independently calculated.

InterPro domain enrichment

To identify enriched InterPro domains for the novel genes, the observed number of each domain among novel genes was compared with the expected number among all genes using the Chi-square test. InterPro domains with P-values < 0.005 were regarded as being enriched.

Identification of tandemly duplicated genes (TDG)

OrthoMCL [57] was used to identify orthologous groups in v3.0 genes. Genes in the same orthologous group and located next to each other on one chromosome were considered to be TDGs.

Availability of supporting data

The sequence data supporting the results of this article are available in the National Center for Biotechnology Information (NCBI) Sequence Read Archive with accession number SRP139269 (PacBio: SRX5437838 and SRX5437837; Hi-C: SRX3918394, SRX3918395; 10X: SRX3918396). Genome sequences and the corresponding annotations, in GFF3 format, are both available from an International Cucurbits Genomics Initiative (ICUGI) FTP server [58] Supporting data and materials are also available in the *GigaScience* GigaDB database [59].

Declarations

List of abbreviations

FL-LTR, full-length long terminal retrotransposon; Hi-C, high-throughput chromosome conformation capture; indel, insertion/deletion (of bases); LTR, long terminal retrotransposon; NCBI, National Center for Biotechnology Information; PacBio, Pacific Biosciences; RNA-seq, RNA sequencing; SMRT, single-molecule real-time; TPST, tyrosylprotein sulfotransferase

Ethics approval and consent to participate

Not applicable.

Consent for publication

Not applicable.

Competing interests

The authors declare that they have no competing interests.

Funding

This work was supported by the China National Key Research and Development Program for Crop Breeding (grant number 2016YFD0100307 to Z.Z.), the National Science Fund for Excellent Young Scholars (grant number 31322047 to Z.Z.), the National Natural Science Foundation of China (grant number 31772304 to Z.Z.), and the National Youth Top-notch Talent Support Program in China (Z.Z.). This work was also supported by the Science and Technology Innovation Program of Chinese Academy of Agricultural Science (CAAS-ASTIP-IVFCAAS).

Authors' contributions

Z.Z. conceived and designed the research. S.W. and W.H. participated in the material preparation. W.H., J.R. and H.L. performed the genome assembly and scaffolding. Q.L., H.L., Q.Z., and Y.X. performed the annotation and comparative analysis. Z.Z. wrote the manuscript. S.H. revised the manuscript. All authors read and approved the final version of the manuscript.

Acknowledgments

We would like to thank Qingyong Yang for help with the Hi-C data analysis.

References

1. Woycicki R, Witkowicz J, Gawronski P, Dabrowska J, Lomsadze A, Pawelkowicz M, et al. The genome sequence of the North-European cucumber (*Cucumis sativus* L.) unravels evolutionary adaptation mechanisms in plants. PLoS One. 2011;6:e22728.
2. Li Z, Zhang Z, Yan P, Huang S, Fei Z and Lin K. RNA-Seq improves annotation of protein-coding genes in the cucumber genome. BMC Genomics. 2011;12:540.
3. Yang L, Koo D, Li Y, Zhang X, Luan F, Havey M, et al. Chromosome rearrangements during domestication of cucumber as revealed by high-density genetic mapping and draft genome assembly. Plant J. 2012;71:895-906.
4. Huang S, Li R, Zhang Z, Li L, Gu X, Fan W, et al. The genome of the cucumber, *Cucumis sativus* L. Nat Genet. 2009;41:1275-81.
5. Huang S, Li R, Zhang Z, Li L, Gu X, Fan W, et al. Genomic data for the domestic cucumber (*Cucumis sativus var. sativus* L.). GigaScience. 2011, http://dx.doi.org/10.5524/100025
6. Qi J, Liu X, Shen D, Miao H, Xie B, Li X, et al. A genomic variation map provides insights

into the genetic basis of cucumber domestication and diversity. Nat Genet. 2013;45:1510-5.

7. Maumus F and Quesneville H. Impact and insights from ancient repetitive elements in plant genomes. Curr Opin Plant Biol. 2016;30:41-6.

8. Bickhart DM, Rosen BD, Koren S, Sayre BL, Hastie AR, Chan S, et al. Single-molecule sequencing and chromatin conformation capture enable de novo reference assembly of the domestic goat genome. Nat Genet. 2017;49:643-50.

9. Daccord N, Celton JM, Linsmith G, Becker C, Choisne N, Schijlen E, et al. High-quality de novo assembly of the apple genome and methylome dynamics of early fruit development. Nat Genet. 2017;49:1099-106.

10. Du H, Yu Y, Ma Y, Gao Q, Cao Y, Chen Z, et al. Sequencing and de novo assembly of a near complete indica rice genome. Nat Commun. 2017;8:15324.

11. Gordon D, Huddleston J, Chaisson MJ, Hill CM, Kronenberg ZN, Munson KM, et al. Long-read sequence assembly of the gorilla genome. Science. 2016;352:aae0344.

12. Jiao WB, Accinelli GG, Hartwig B, Kiefer C, Baker D, Severing E, et al. Improving and

correcting the contiguity of long-read genome assemblies of three plant species using optical mapping and chromosome conformation capture data. Genome Res. 2017;27:778-86.

13. Jiao Y, Peluso P, Shi J, Liang T, Stitzer MC, Wang B, et al. Improved maize reference genome with single-molecule technologies. Nature. 2017;546:524-7.

14. Dudchenko O, Batra SS, Omer AD, Nyquist SK, Hoeger M, Durand NC, et al. *De novo* assembly of the Aedes aegypti genome using Hi-C yields chromosome-length scaffolds. Science. 2017;356:92-5.

15. Yeo S, Coombe L, Warren RL, Chu J and Birol I. ARCS: scaffolding genome drafts with linked reads. Bioinformatics. 2018;34:725-31.

16. Zhang G, Liu K, Li Z, Lohaus R, Hsiao Y, Niu S, et al. The Apostasia genome and the evolution of orchids. Nature. 2017;549:379-83.

17. Lam KK, LaButti K, Khalak A and Tse D. FinisherSC: a repeat-aware tool for upgrading de novo assembly using long reads. Bioinformatics. 2015;31:3207-9.

18. Walker BJ, Abeel T, Shea T, Priest M, Abouelliel A, Sakthikumar S, et al. Pilon: an

integrated tool for comprehensive microbial variant detection and genome assembly improvement. PLoS One. 2014;9:e112963.

19. Ren Y, Zhang Z, Liu J, Staub JE, Han Y, Cheng Z, et al. An integrated genetic and cytogenetic map of the cucumber genome. PLoS One. 2009;4:e5795.

20. Zhang W, Pan J, He H, Zhang C, Li Z, Zhao J, et al. Construction of a high density integrated genetic map for cucumber (*Cucumis sativus* L.). Theor Appl Genet. 2012;124:249-59.

21. Zhou Q, Miao H, Li S, Zhang S, Wang Y, Weng Y, et al. A sequencing-based linkage map of cucumber. Molecular Plant. 2015;8:961-3.

22. Sun J, Zhang Z, Zong X, Huang S, Li Z and Han Y. A high-resolution cucumber cytogenetic map integrated with the genome assembly. BMC Genomics. 2013;14:461.

23. Ganal. M, Riede. I and Hemleben. V. Organization and sequence analysis of two related satellite DNAs in cucumber (*Cucumis sativus* L.). J Mol Evol. 1986;23:23-30.

24. Ganal. M and Hemleben. V. Insertion and amplification of a DNA sequence in satellite DNA of *Cucumis sativus* L. (cucumber). Theor Appl Genet. 1988;75:357-61.

25. Han Y, Zhang Z, Liu J, Lu J, Huang S and Jin W. Distribution of the tandem repeat sequences and karyotyping in cucumber (*Cucumis sativus* L.) by fluorescence in situ hybridization. Cytogenet Genome Res. 2008;122:80-8.

26. Haas BJ, Salzberg SL, Zhu W, Pertea M, Allen JE, Orvis J, et al. Automated eukaryotic gene structure annotation using EVidenceModeler and the program to assemble spliced alignments. Genome Biol. 2008;9:R7.

27. Murray MG and Thompson WF. Rapid isolation of high molecular weight plant DNA. Nucleic Acids Res. 1980;8:4321-6.

28. 10X Genomics. https://www.10xgenomics.com.

29. Belton JM, McCord RP, Gibcus JH, Naumova N, Zhan Y and Dekker J. Hi-C: a comprehensive technique to capture the conformation of genomes. Methods. 2012;58:268-76.

30. Wingett S, Ewels P, Furlan-Magaril M, Nagano T, Schoenfelder S, Fraser P, et al. HiCUP: pipeline for mapping and processing Hi-C data. F1000Res. 2015;4:1310.

31. Raymond O, Gouzy J, Just J, Badouin H, Verdenaud M, Lemainque A, et al. The Rosa

genome provides new insights into the domestication of modern roses. Nat Genet. 2018;50:772-7.

32. Koren S, Walenz BP, Berlin K, Miller JR, Bergman NH and Phillippy AM. Canu: scalable and accurate long-read assembly via adaptive k-mer weighting and repeat separation. Genome Res. 2017;27:722-36.

33. Altschul SF, Gish W, Miller W, Myers EW and Lipman DJ. Basic local alignment search tool. J Mol Biol. 1990;215:403-10.

34. Kurtz S, Phillippy A, Delcher AL, Smoot M, Shumway M, Antonescu C, et al. Versatile and open software for comparing large genomes. Genome Biology. 2004;5:R12.

35. Tang H, Zhang X, Miao C, Zhang J, Ming R, Schnable JC, et al. ALLMAPS: robust scaffold ordering based on multiple maps. Genome Biol. 2015;16:3.

36. Coombe L, Zhang J, Vandervalk BP, Chu J, Jackman SD, Birol I, et al. ARKS: chromosome-scale scaffolding of human genome drafts with linked read kmers. BMC Bioinformatics. 2018;19:234.

37. RepeatModeler. http://www.repeatmasker.org/RepeatModeler/.

38. Ouyang S and Buell CR. The TIGR Plant Repeat Databases: a collective resource for the identification of repetitive sequences in plants. Nucleic Acids Res. 2004;32:D360-3.

39. RepeatMasker. http://www.repeatmasker.org.

40. Xu Z and Wang H. LTR_FINDER: an efficient tool for the prediction of full-length LTR retrotransposons. Nucleic Acids Res. 2007;35:W265-8.

41. Edgar RC. MUSCLE: a multiple sequence alignment method with reduced time and space complexity. BMC Bioinformatics. 2004;5:113.

42. Rice P, Longden I and Bleasby A. EMBOSS: the European Molecular Biology Open Software Suite. Trends Genet. 2000;16:276-7.

43. Nystedt B, Street NR, Wetterbom A, Zuccolo A, Lin YC, Scofield DG, et al. The Norway spruce genome sequence and conifer genome evolution. Nature. 2013;497:579-84.

44. Augustus. http://augustus.gobics.de.

45. Majoros WH, Pertea M and Salzberg SL. TigrScan and GlimmerHMM: two open source ab initio eukaryotic gene-finders. Bioinformatics. 2004;20:2878-9.

46. Johnson AD, Handsaker RE, Pulit SL, Nizzari MM, O'Donnell CJ and de Bakker PI. SNAP:

a web-based tool for identification and annotation of proxy SNPs using HapMap. Bioinformatics. 2008;24:2938-9.

47. Wei G, Tian P, Zhang F, Qin H, Miao H, Chen Q, et al. Integrative analyses of nontargeted volatile profiling and transcriptome data provide molecular insight into VOC diversity in cucumber plants (*Cucumis sativus*). Plant Physiol. 2016;172:603-18.

48. Qiu L, Jiang B, Fang J, Shen Y, Fang Z, Rm SK, et al. Analysis of transcriptome in hickory (*Carya cathayensis*), and uncover the dynamics in the hormonal signaling pathway during graft process. BMC Genomics. 2016;17:935.

49. Iwata H and Gotoh O. Benchmarking spliced alignment programs including Spaln2, an extended version of Spaln that incorporates additional species-specific features. Nucleic Acids Res. 2012;40:e161.

50. UniProt. https://www.uniprot.org.

51. The Arabidopsis Information Resource (TAIR). https://www.arabidopsis.org.

52. Li H and Durbin R. Fast and accurate short read alignment with Burrows-Wheeler transform. Bioinformatics. 2009;25:1754-60.

53. GenomeAnalysisTK. http://www.broadinstitute.org/gatk.

54. Python svgwrite. https://pypi.org/project/svgwrite/.

55. Trapnell C, Pachter L and Salzberg SL. TopHat: discovering splice junctions with RNA-Seq. Bioinformatics. 2009;25:1105-11.

56. Trapnell C, Williams BA, Pertea G, Mortazavi A, Kwan G, van Baren MJ, et al. Transcript assembly and quantification by RNA-Seq reveals unannotated transcripts and isoform switching during cell differentiation. Nat Biotechnol. 2010;28:511-5.

57. Li L, Stoeckert CJ, Jr. and Roos DS. OrthoMCL: identification of ortholog groups for eukaryotic genomes. Genome Res. 2003;13:2178-89.

58. Cucurbit genomics. ftp://cucurbitgenomics.org/pub/cucurbit/genome/cucumber/Chinese_long/.

59 Li Q; Li H; Huang W; Xu Y; Zhou Q; Wang S; Ruan J; Huang S; Zhang Z (2019): Supporting data for "A chromosome-scale genome assembly of cucumber (*Cucumis sativus* L.)" GigaScience Database. http://dx.doi.org/10.5524/100603

Figure legends

Figure 1. Landscape of the seven pseudo-chromosome (chr) sequences.

All included contigs are shown. Cytogenetic map [22] is integrated with the sequences. Arrows mark positions of the centromeres (Cen). The distribution of satellite and repetitive sequences along the contigs is illustrated below. Fosmid clones are marked in green and red on the seven chromosomes, and the imaginary lines connect the physical locations and approximate locations of assembled chromosomes.

Figure 2. Correlation of genome assembly with genetic maps and Hi-C data.

A. Integrated genetic and physical maps of the cucumber genome assembly. Super-scaffolds of the genome assembly (middle) were anchored to the four linkage groups (left and right): map.1 (green) [3], map.2 (orange) [21], map.3 (light blue) [20], map.4 (pink) [19].

B. Heat map of Hi-C contact information. Pixel colors represent different normalized counts of Hi-C links between 30 kb non-overlapping windows for all seven chromosomes (chr) on a logarithmic scale.

Figure 3. Novel repetitive sequences and genes in assembly v3.0.

A. Sizes of various types of repetitive sequences in the v2.0 and v3.0 assemblies. DNA, DNA transposons; LINE, Long interspersed nuclear elements; SINE, Short interspersed nuclear elements; LTRc, Copia long terminal repeat retrotransposons; LTRg, Gypsy long terminal repeat retrotransposons; LTRo, Other LTR categories; Unknown, unknown type.

B. The number of full-length long terminal retrotransposons (FL-LTRs) in v2.0 and v3.0.

C. A newly predicted FL-LTR in v3.0. TSR, Target site repeat; PBS, Primer binding site; PPT, Primer polypurine tract; IN, Integrase; RT, Reverse transcriptase.

D. An example showing the newly assembled multiple tyrosylprotein sulfotransferase (*TPST*) genes in V3.0. b'–e' are all *TPST* genes, corresponding to CsaV3_1G013960, CsaV3_1G013970, CsaV3_1G013980, and CsaV3_1G013990, respectively.

Figure 4. Distribution of GC content for the whole genome and novel sequences in v3.0.

Additional files

Additional file 1. Summary of sequencing data from PacBio, 10X Genomics and Hi-C platforms.

Additional file 2. Distribution of gaps across the meta-assembly and the six initial assemblies. The out circle relates to the seven pseudo-chromosomes of cucumber. Circles a–f indicate the contig tracks of the meta-assembly and six initial assemblies, of which gaps are colored in white. a, Meta assembly; b, CANU1 assembly; c, CANU2 assembly; d, FALCON1 assembly; e, FALCON2 assembly; f, FALCON3 assembly; g, FALCON4 assembly.

Additional file 3. Summary of the previous sequence reads generated from Illumina and Sanger libraries.

Additional file 4. Genome assembly statistics.

Scaffolds were built using 10X Genomics linked reads based on assembled contigs, and were

clustered and ordered into super-scaffolds based on Hi-C data.

Additional file 5. Whole-genome synteny for genomes of v3.0 and v2.0.

Bold lines in orange and bold lines in blue indicate chromosomes of v3.0 and v2.0, respectively, and gold lines indicate alignment between the v2.0 and v3.0 genomes.

Additional file 6. Sequence Read Archive (SRA) IDs of the RNA-seq data used in this study.

Additional file 7. Length and percentage of various repetitive sequences in v3.0.

Additional file 8. Distribution of the times of long terminal retrotransposon (LTR) insertion events in v2.0 and v3.0.

Additional file 9. Annotation of the newly assembled genes in v3.0.

Additional file 10. Box-plot for length of genes, coding sequences, and introns in the whole set of genes and newly predicted genes.

The average length of genes, exons and introns are labeled in the figure.

Additional file 11. Different alignment types of genes between v2.0 and v3.0.

a. Based on the alignments of genomes v3.0 and v2.0, 1970 fragmented genes were found in v2.0, which correspond to 932 genes in V3.0.

b. 687 genes in v2.0 are split into 337 in v3.0

Additional file 12. GC content of novel genes and the whole genome.

Additional file 13. Enriched InterPro terms for the novel genes in v3.0.

Additional file 14. Summary of the software, parameters, and results of the meta-assembly process.

Figure1

Click here to access/download;Figure;Figure1_correct.pdf

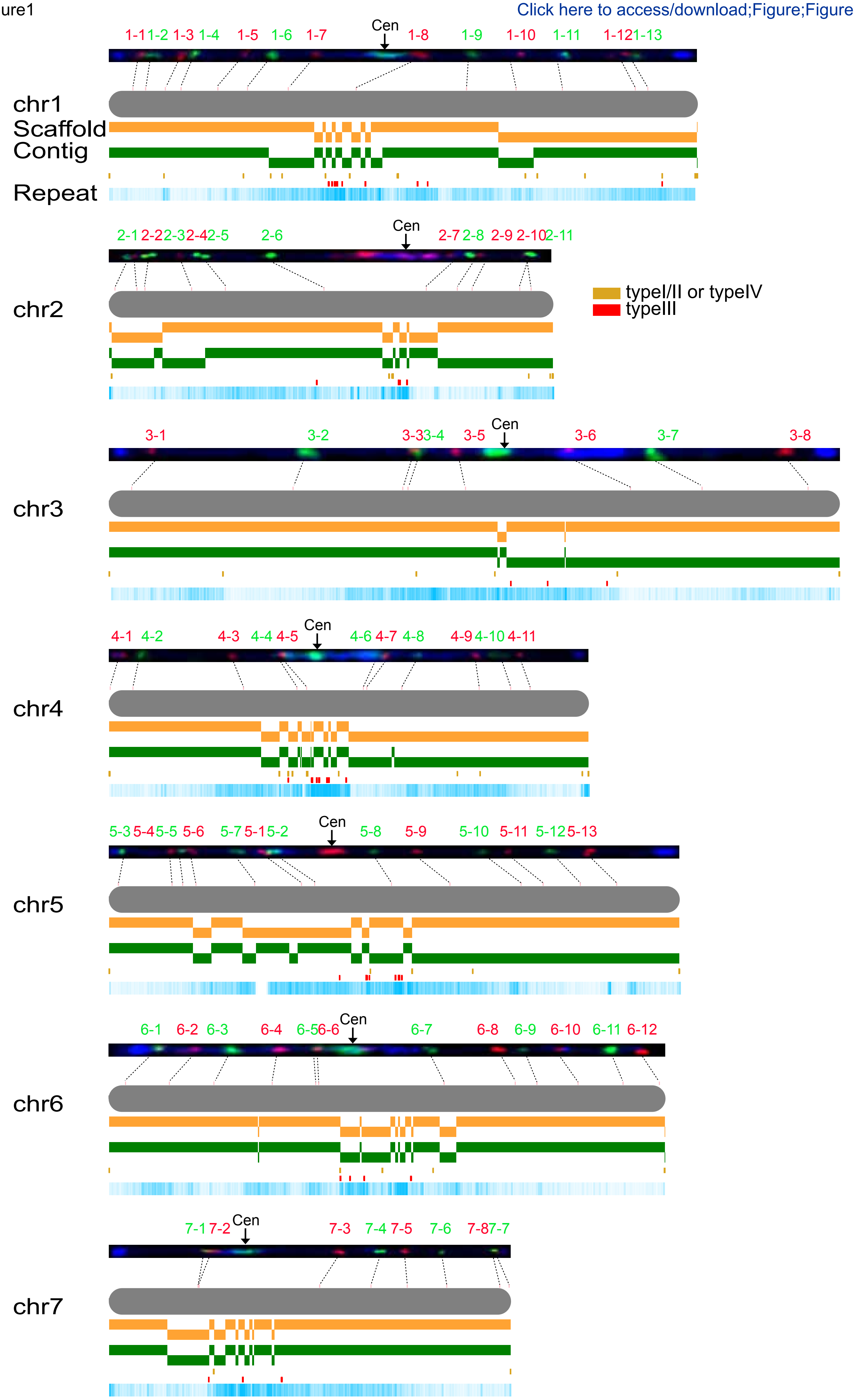

Figure2

Click here to access/download;Figure;Figure2_correct.pdf

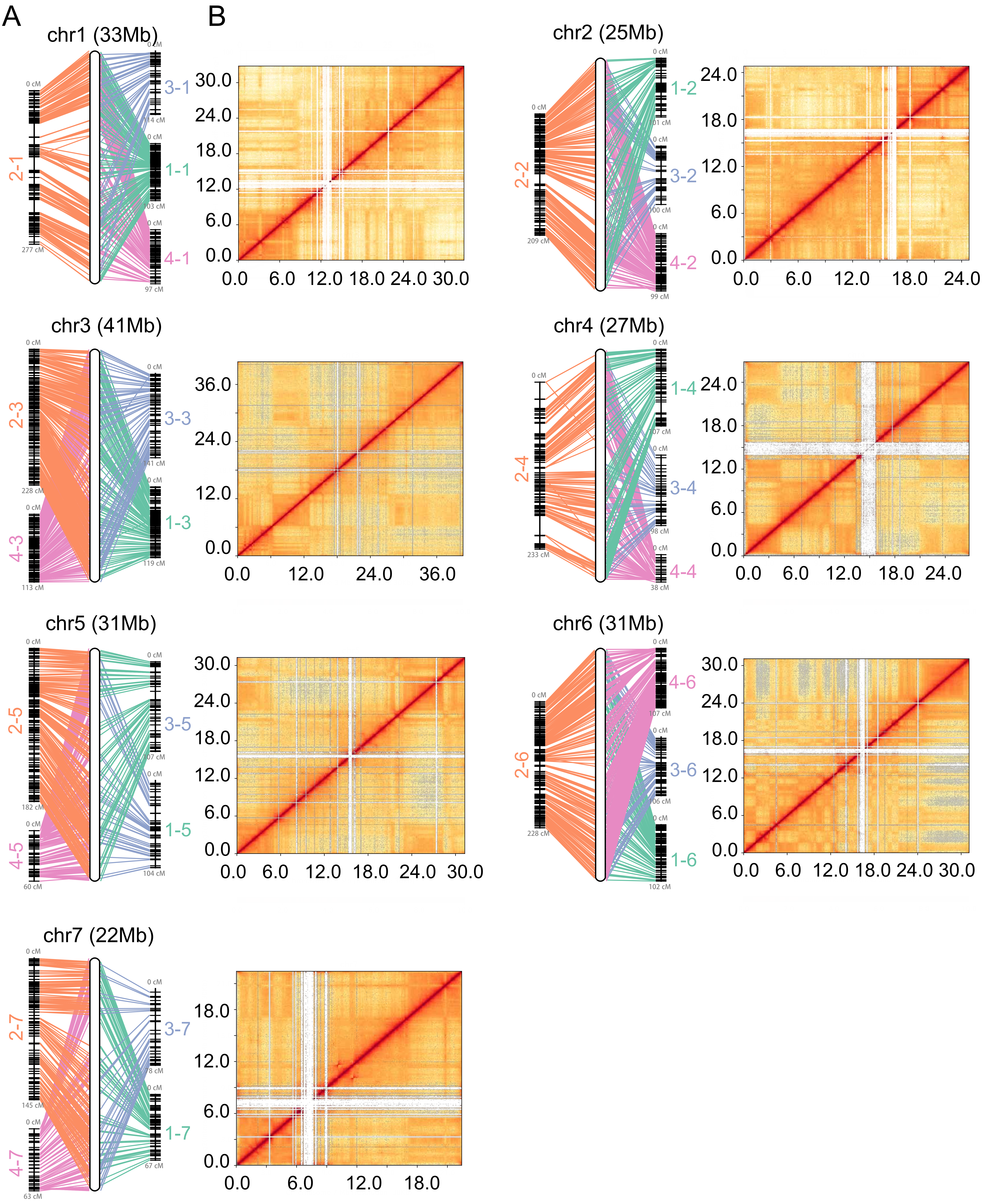

Figure3

Click here to access/download;Figure;Figure3_correct.pdf

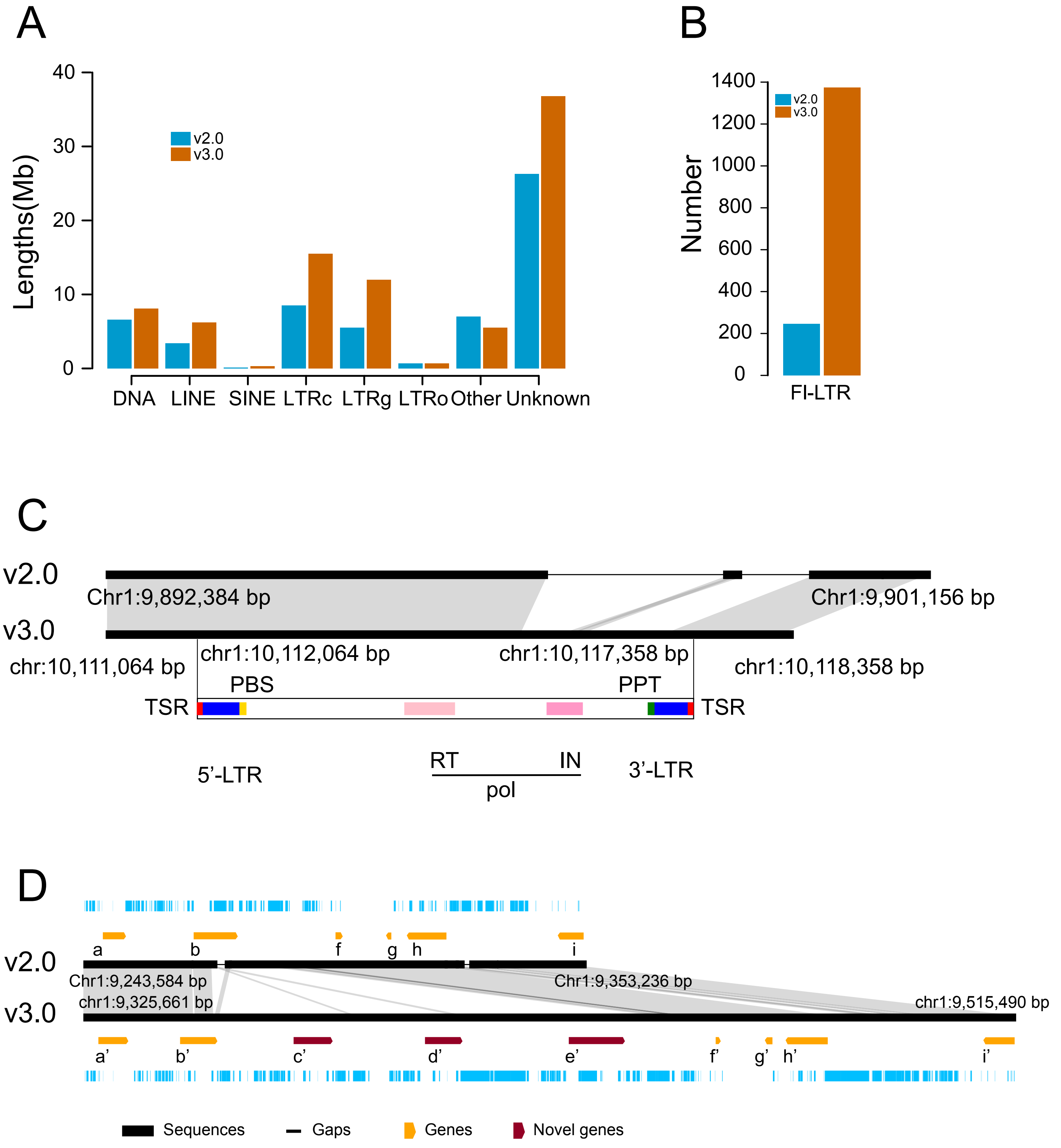

Figure4

Click here to access/download;Figure;Figure4

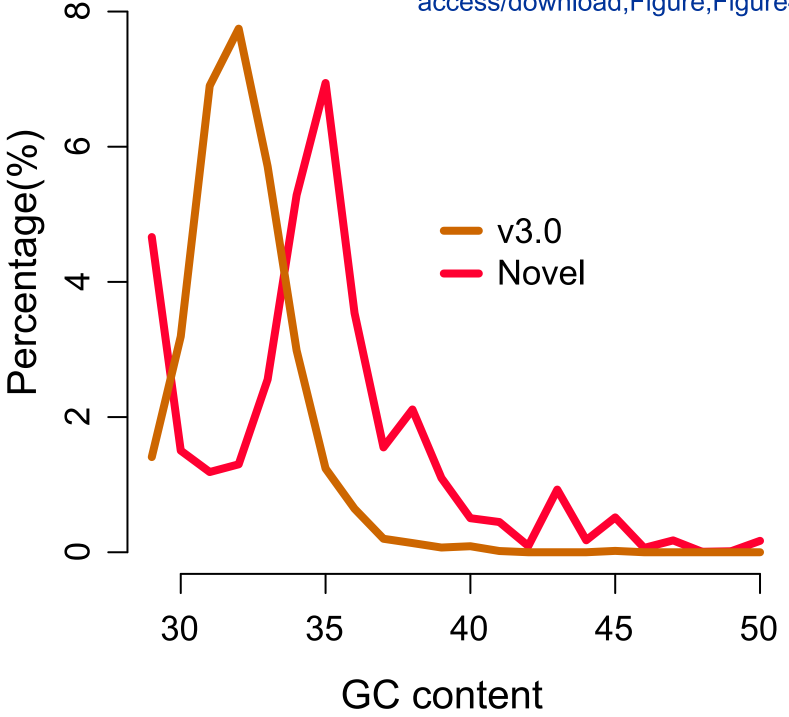

Additional file1

Click here to access/download
Supplementary Material
Additional file 1.docx

Additional file2

Click here to access/download
Supplementary Material
Additional file 2.pdf

Additional file3

Click here to access/download
Supplementary Material
Additional file 3.docx

Additional file4

Click here to access/download
Supplementary Material
Additional file 4.docx

Additional file5

Click here to access/download
Supplementary Material
Additional file 5.pdf

Additional file6

Click here to access/download
Supplementary Material
Additional file 6.docx

Additional file7

Click here to access/download
Supplementary Material
Additional file 7.docx

Additional file8

Click here to access/download
Supplementary Material
Additional file 8.pdf

Additional file9

Click here to access/download
Supplementary Material
Additional file 9.xlsx

Additional file10

Click here to access/download
Supplementary Material
Additional file 10.pdf

Additional file11

Click here to access/download
Supplementary Material
Additional file 11.pdf

Additional file12

Click here to access/download
Supplementary Material
Additional file 12.pdf

Additional file13

Click here to access/download
Supplementary Material
Additional file 13.xlsx

Additional file14

Click here to access/download
Supplementary Material
Additional file 14.docx

Personal Cover Click here to access/download;Personal Cover;cucumber V3 CV 20190520.docx

Dear Dr. Hongling Zhou

We greatly appreciate the dedication of the reviewers and the editor to help us to improve the manuscript. According to the reviewer and editors's comments and suggestions, we have revised our manuscript and upload them again.

Thank you in advance for considering this work.

Sincerely yours,

Zhonghua Zhang
Institute of Vegetables and Flowers, Chinese Academy of Agricultural Sciences
No. 12, Zhong Guan Cun Nan Da Jie, Beijing, 100081, China
Tel: +86-10-62117612
Mobile Phone: +8613699205910
Email: zhangzhonghua@caas.cn
