# Supplementary material for: A chromosome-scale genome assembly of cucumber (Cucumis sativus L.)
Source: Gigascience. 2019 Jun 18;8(6):giz072. doi: 10.1093/gigascience/giz072 (PMC6582320; doi:10.1093/gigascience/giz072)
Supplement: giz072_Supplemental_Files [file giz072_supplemental_files.zip › Additional file 1.docx]

**Additional file 1**

|  | PacBio | 10X | Hi-C |
| --- | --- | --- | --- |
| Number of Bases | 16,190,613,962 | 20,234,933,100 | 68,432,467,500 |
| Number of Reads | 2,099,106 | 134,899,554 | 456,216,450 |
| N50 Read Length (bp) | 10,853 | 150 | 150 |
| Mean Reads Length (bp) | 7,713 | 150 | 150 |
