# Supplementary material for: A chromosome-scale genome assembly of cucumber (Cucumis sativus L.)
Source: Gigascience. 2019 Jun 18;8(6):giz072. doi: 10.1093/gigascience/giz072 (PMC6582320; doi:10.1093/gigascience/giz072)
Supplement: giz072_Supplemental_Files [file giz072_supplemental_files.zip › Additional file 14.docx]

**Additional file 14**

| Step | Assembly | Software | Parameters | Total length (bp) | No. of contigs | N50 length (bp) | Min length (bp) | Max length (bp) |
| --- | --- | --- | --- | --- | --- | --- | --- | --- |
| Contig assemblies | CANU1 | CANU 1.7 | genomeSize=367.0m corOutCoverage=100 -pacbio-raw | 235,590,438 | 1,071 | 2,534,221 | 1,066 | 8,569,825 |
|  | CANU2 | CANU 1.7 | genomeSize=367.0m corOutCoverage=40 -pacbio-raw | 233,830,228 | 1,094 | 2,409,471 | 1,068 | 12,402,379 |
|  | FALCON1 | FALCON/til-r-20180523 | --minpci 97 --deltapci 1.5 --minovl 1000 --minwing 1000 | 233,624,436 | 673 | 3,391,143 | 2,505 | 12,994,526 |
|  | FALCON2 | FALCON/til-r-20180523 | --minpci 97 --deltapci 1.5 --minovl 1500 --minwing 1500 | 231,912,996 | 633 | 3,363,682 | 2,505 | 12,994,526 |
|  | FALCON3 | FALCON/til-r-20180523 | --minpci 97.5 --deltapci 2 --minovl 2000 --minwing 2000 | 228,647,723 | 589 | 3,363,804 | 2,152 | 10,746,046 |
|  | FALCON4 | FALCON/til-r-20180523 | --minpci 98 --deltapci 2 --minovl 1500 --minwing 1500 | 226,619,428 | 590 | 3,604,840 | 2,505 | 10,742,967 |
| Meta-assembly | Meta | CANU 1.7 | -trim-assemble genomeSize=367.0m cnsConsensus=utgcns minOverlapLength=10000 minReadLength=10000 -pacbio-corrected | 232,317,497 | 195 | 9,113,993 | 14,269 | 26,778,834 |
