# Supplementary material for: A chromosome-scale genome assembly of cucumber (Cucumis sativus L.)
Source: Gigascience. 2019 Jun 18;8(6):giz072. doi: 10.1093/gigascience/giz072 (PMC6582320; doi:10.1093/gigascience/giz072)
Supplement: giz072_Supplemental_Files [file giz072_supplemental_files.zip › Additional file 3.docx]

**Additional file 3**

| Sequencing Method |  | Clone Insert Size | Total  Lengths  (Gb) | Sequence  Depth |
| --- | --- | --- | --- | --- |
| Sanger | Bac end | 100 kb | 0.01 | 0.06 |
|  | Fosmid | 40 kb | 0.14 | 0.63 |
|  |  | 2-3 kb | 0.88 | 3.90 |
|  | Plasmid | 4-5 kb | 0.14 | 0.64 |
|  |  | 5-7 kb | 0.15 | 0.65 |
| Illumina | | 167 bp | 16.61 | 73.46 |
|  |  | 170 bp | 14.15 | 62.54 |
|  |  | 760 bp | 10.64 | 47.05 |
|  |  | 5 K | 0.89 | 3.96 |
