# Supplementary material for: A chromosome-scale genome assembly of cucumber (Cucumis sativus L.)
Source: Gigascience. 2019 Jun 18;8(6):giz072. doi: 10.1093/gigascience/giz072 (PMC6582320; doi:10.1093/gigascience/giz072)
Supplement: giz072_Supplemental_Files [file giz072_supplemental_files.zip › Additional file 4.docx]

**Additional file 4**

|  | Contig | Scaffold | Super-scaffold |
| --- | --- | --- | --- |
| Assembly length (Mb) | 226.2 | 226.2 | 226.2 |
| No. Ns (kb) | 0 | 0.2 | 35.2 |
| No. Contigs | 174 | - | - |
| Contig N50 (Mb) | 8.9 | - | - |
| Largest Contig (Mb) | 21.7 | - | - |
| No. Scaffolds | - | 157 | 85 |
| Scaffold N50 (Mb) | - | 11.5 | 31.1 |
| Scaffold L50 |  | 8 | 4 |
| Largest Scaffold (Mb) | - | 21.7 | 40.9 |
