# Supplementary material for: A chromosome-scale genome assembly of cucumber (Cucumis sativus L.)
Source: Gigascience. 2019 Jun 18;8(6):giz072. doi: 10.1093/gigascience/giz072 (PMC6582320; doi:10.1093/gigascience/giz072)
Supplement: giz072_Supplemental_Files [file giz072_supplemental_files.zip › Additional file 6.docx]

**Additional file 6**

| SRA Study | Bases (G) | Tissue | Runs |
| --- | --- | --- | --- |
| SRP048926^1^ | 40.7 | Pedicel, stalk, fruit | 6 |
| SRP071224^2^ | 49.5 | Petiole of Old Leaf, Old leaf, Petiole of Young Leaf, Young Leaf, Stem, Root, True Leaf of 4-Week-Old Seedlings,  Flesh of 3-Week-Old Fruit，  Peel of 3-Week-Old Fruit，Flesh of 2-Week-Old Fruit，  Cotyledon of 4-Week-Old Seedlings，Peel of 2-Week-Old Fruit, Flesh of 1-Week-Old Fruit  , Peel of 1-Week-Old Fruit,  Flesh of Unfertilized Ovary, Peel of Unfertilized Ovary, Unfertilized Ovary (not grew), Male Flower, Male Flower Bud, Female Floewr, Tendril, Hypocotyl of 4-Week-Old Seedlings, Root of 4-Week-Old Seedlings | 23 |
| SRP008779^3^ | 31.5 | root, stem, leaf, male flower, female flower, ovary, expanded ovary under fertilization (7 days after flowering), expanded ovary not fertilized (7 days after flowering), base part of tendril, and tendril | 10 |

^1^ 3 tissues RNA data from [1]

^2^ 23 tissues RNA data from [2]

^3^ 10 tissues RNA data from [3]

1. Zhao J, Li Y, Ding L, Yan S, Liu M, Jiang L, et al. Phloem transcriptome signatures underpin the physiological differentiation of the pedicel, stalk and fruit of cucumber (*Cucumis sativus* L.). Plant Cell Physiol. 2016;57:19-34.

2. Wei G, Tian P, Zhang F, Qin H, Miao H, Chen Q, et al. Integrative Analyses of Nontargeted Volatile Profiling and Transcriptome Data Provide Molecular Insight into VOC Diversity in Cucumber Plants (*Cucumis sativus*). Plant Physiol. 2016;172:603-18.

3. Li Z, Zhang Z, Yan P, Huang S, Fei Z and Lin K. RNA-Seq improves annotation of protein-coding genes in the cucumber genome. BMC Genomics. 2011;12:540.
