# Supplementary material for: A chromosome-scale genome assembly of cucumber (Cucumis sativus L.)
Source: Gigascience. 2019 Jun 18;8(6):giz072. doi: 10.1093/gigascience/giz072 (PMC6582320; doi:10.1093/gigascience/giz072)
Supplement: giz072_Supplemental_Files [file giz072_supplemental_files.zip › Additional file 7.docx]

**Additional file 7**

| Classification | Length (bp) | Percentage (%) |
| --- | --- | --- |
| DNA | 8,146,528 | 3.60 |
| SINE | 311,052 | 0.14 |
| LINE | 6,249,968 | 2.76 |
| LTR/Copia | 15,538,363 | 6.87 |
| LTR/Gypsy | 11,959,227 | 5.29 |
| Others LTR | 709,874 | 0.31 |
| Low_complexity | 1,210,492 | 0.54 |
| Simple_repeat | 3,697,033 | 1.63 |
| RC/Helitron | 303,497 | 0.13 |
| Retroposon | 82,450 | 0.04 |
| Satellite | 19,650 | 0.01 |
| RNA | 204,844 | 0.09 |
| Unknown | 36,795,358 | 16.3 |
| Total | 85,228,336 | 37.7 |
