# Supplementary figures and images for: A chromosome-scale genome assembly of cucumber (Cucumis sativus L.)
Source: Gigascience. 2019 Jun 18;8(6):giz072. doi: 10.1093/gigascience/giz072 (PMC6582320; doi:10.1093/gigascience/giz072)

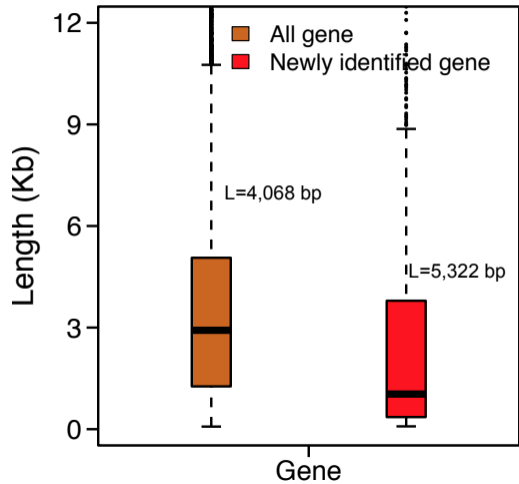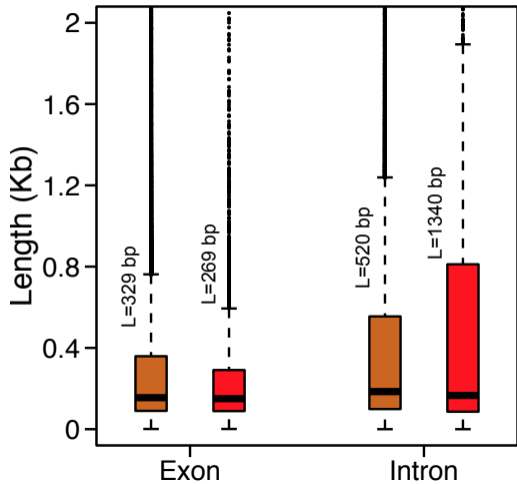

Supplement: giz072_Supplemental_Files [file giz072_supplemental_files.zip › Additional file 10.pdf]

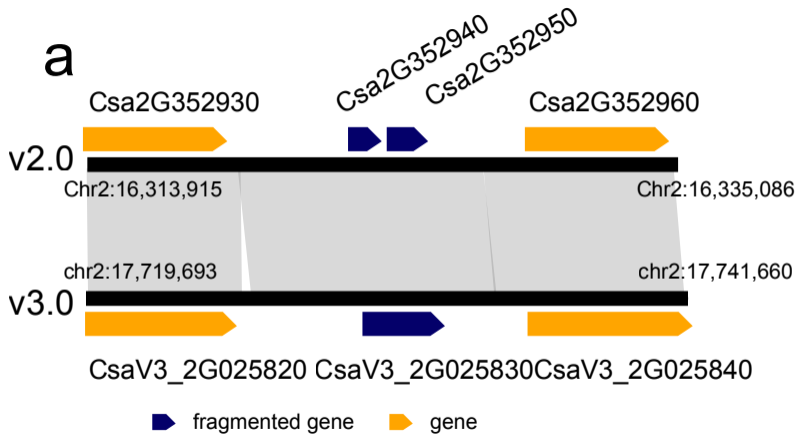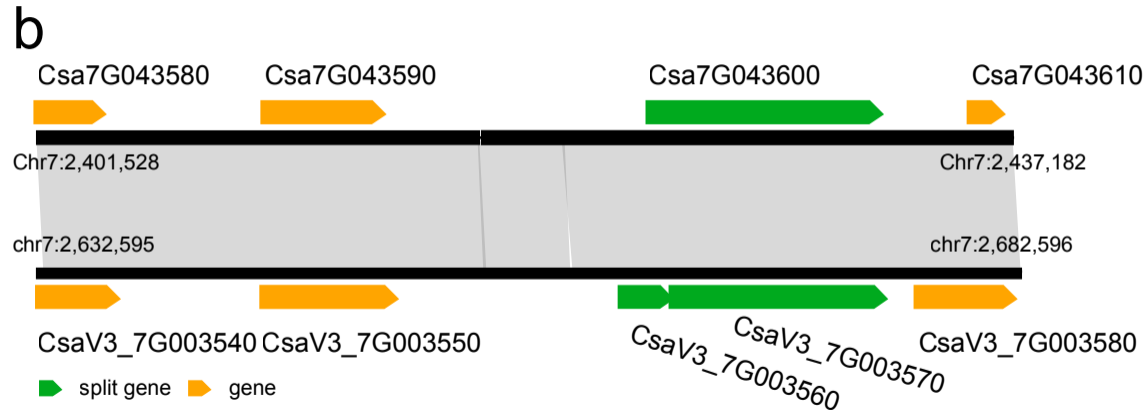

Supplement: giz072_Supplemental_Files [file giz072_supplemental_files.zip › Additional file 11.pdf]

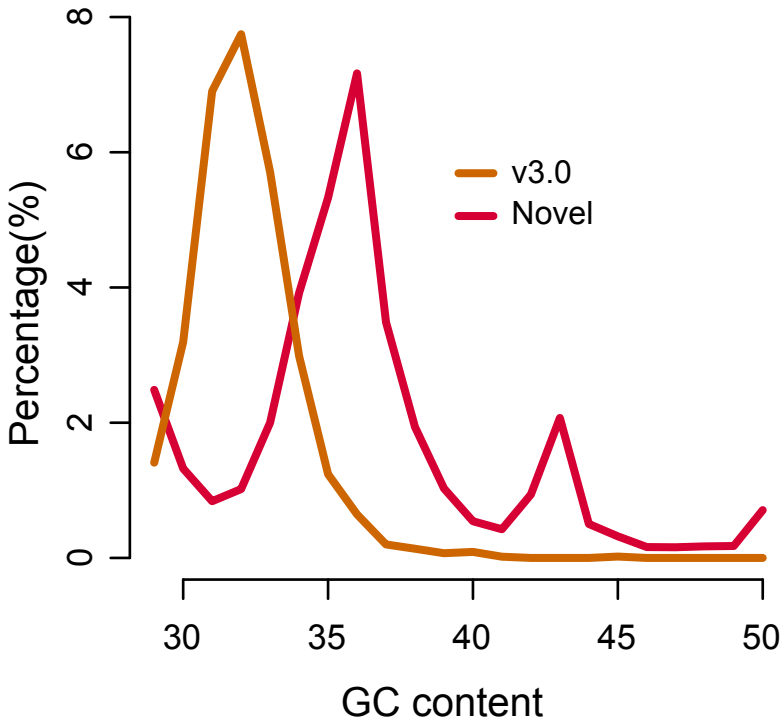

Supplement: giz072_Supplemental_Files [file giz072_supplemental_files.zip › Additional file 12.pdf]

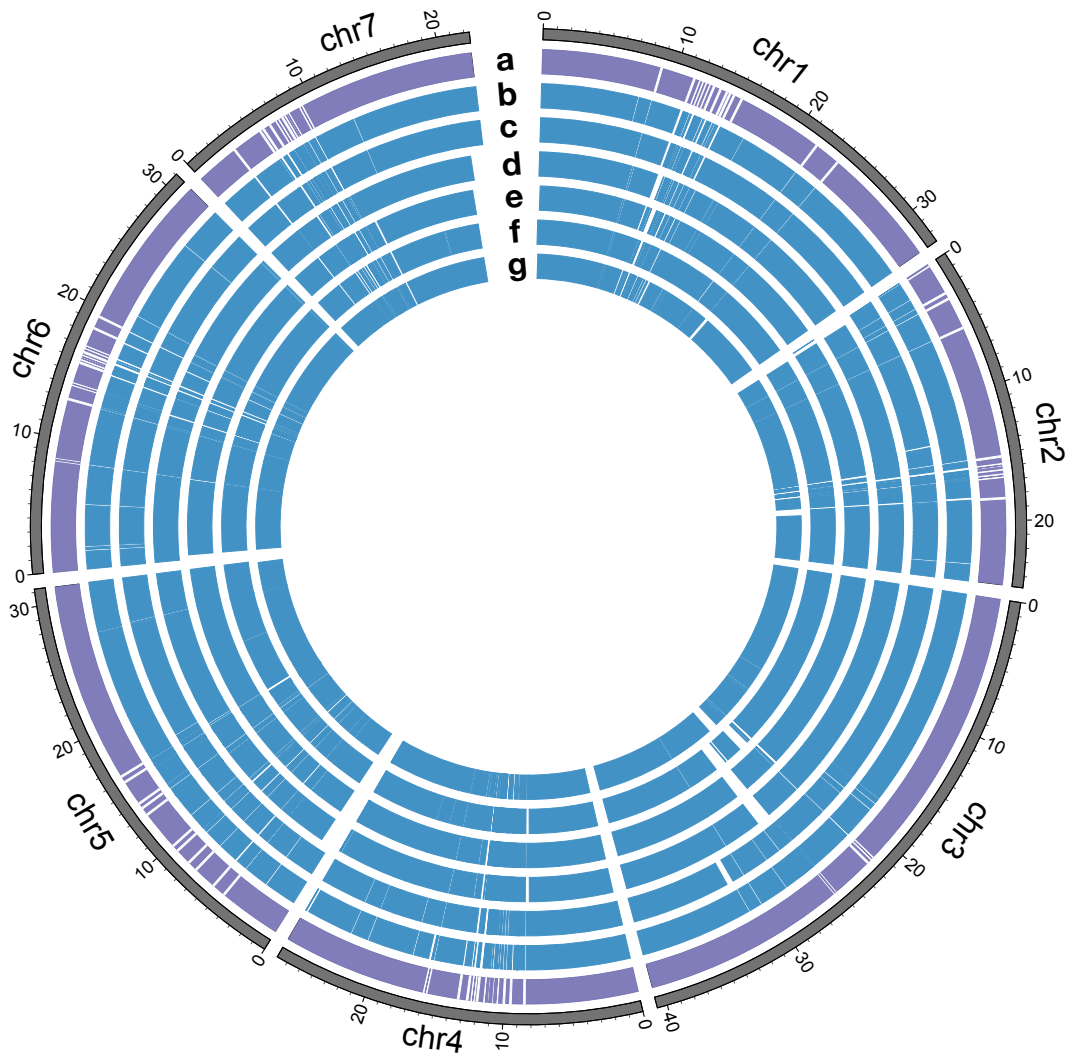

Supplement: giz072_Supplemental_Files [file giz072_supplemental_files.zip › Additional file 2.pdf]

0 1 41 Mb

Chr1

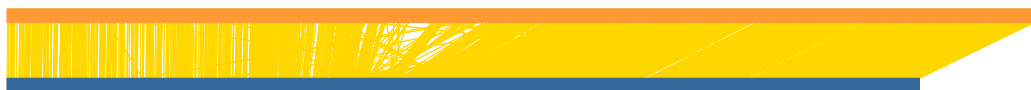

Chr2

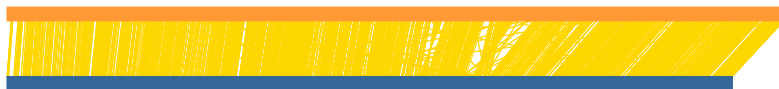

v3.0

v2.0

map

Chr3

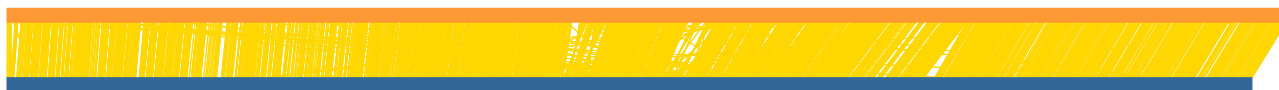

Chr4

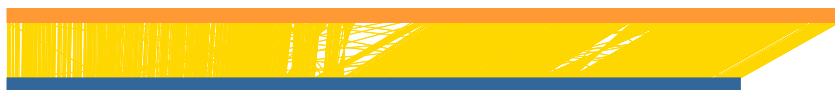

Chr5

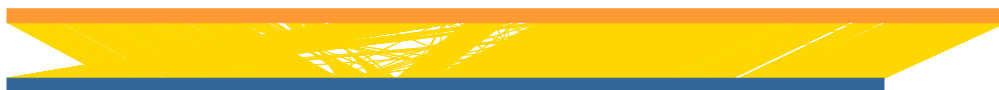

Chr6

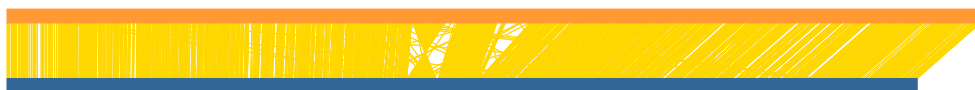

Chr7

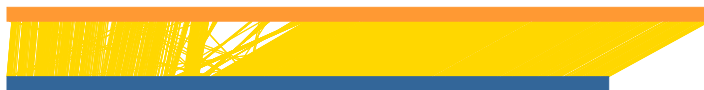

Supplement: giz072_Supplemental_Files [file giz072_supplemental_files.zip › Additional file 5.pdf]

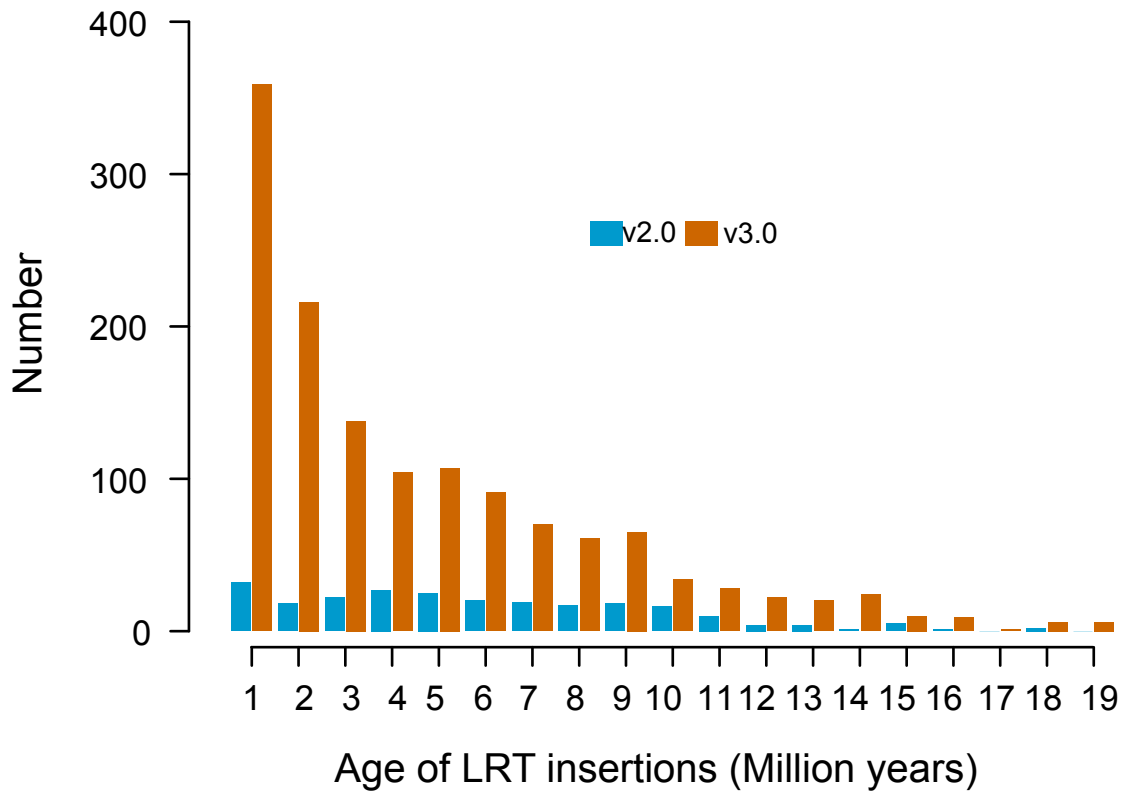

Supplement: giz072_Supplemental_Files [file giz072_supplemental_files.zip › Additional file 8.pdf]
