# Supplementary material for: A chromosome-scale genome assembly of cucumber (Cucumis sativus L.)
Source: Gigascience. 2019 Jun 18;8(6):giz072. doi: 10.1093/gigascience/giz072 (PMC6582320; doi:10.1093/gigascience/giz072)
Supplement: giz072_Response_to_Reviewer_Comments_Original_Submission [file giz072_response_to_reviewer_comments_original_submission.pdf]

Author's Response To Reviewer Comments

Close

Dear Dr. Hongling Zhou

We greatly appreciate the dedication of the reviewers and the editor to help us to improve the manuscript. According to their comments, we have revised our manuscript and we provide a point-by-point response to the editor/reviewers' concerns and indicate how we have modified the manuscript.

Reviewer reports:

Reviewer #1:
24-1-2019

The authors have kindly offered access to the genome sequence and they also have explained that the genome sequence will be available in SRA by the time the paper is published. This completely solves the issue regarding the genome sequence. However, I still have doubts regarding the annotation. Will be the annotation made available in the genebank along with the sequence? As a GFF file in ICUGI? With a GFF file attached to the manuscript as supplementary material? Any of those options would be fine, because they will ease the use of the genome sequence by the community. I hope this issue is clarified soon and so the community can enjoy the great advance presented by the authors. I have no doubts that this paper will be a methodological reference.
Response:
We thank the reviewer for the comments and suggestions. The genome sequences and the corresponding annotations in GFF3 format are available at ICUGI ftp server: ftp://cucurbitgenomics.org/pub/cucurbit/genome/cucumber/Chinese_long/. To further ease the use of the genome by the community, the genome and annotation can also be accessed via Genome Browser at ICUGI.

21-1-2019

The reported V3.0 of the cucumber genome is not just a very useful tool for the cucumber community, but a great standard to follow in future genome assembly projects.
The methods used are appropriate and the results just great.
The manuscript is well written and clear. I have just detected a minor mistake, the authors have misspell telomeric as telemeric. (Although there could be more mistakes, I am not a native English speaker).
Response：
Thanks for your correction. we corrected all the misspell words in the manuscript.

Are the methods appropriate to the aims of the study, are they well described, and are necessary controls included?
Response:
We believe that we used appropriate methods to achieve our aims. Meta-assembled contigs were generated using CANU 1.7 [1] by combining results from two CANU and four FALCON/til-r assemblies with proper parameters (Supplemental Table S8). As a result, we obtained a more completed and accurate genome containing 174 contigs with a total size of 226.2 Mb and an N50 length of 8.9 Mb. To increase the accuracy of the contig sequences, the previously generated Illumina and Sanger reads (Supplemental Table S2) were aligned to the contigs. The potential sequence errors including single nucleotide polymorphism (SNP) and insertion/deletions (InDel) were corrected using Pilon [3] two times with the parameters: --fix all --chunksize 20000000 --mindepth 0.4 --K 65 --gapmargin 150000 --vcf --changes --tracks --minmq 10. The conflicting contigs with the orders of molecular markers from the four genetic maps were manually checked and split using the alignment results against the previous genome assembly (V2.0). Hi-C data were also aligned to the contigs for checking and correcting mis-assemblies. To clarify the methods more clearly, we added more details of genome assembly and annotation in the

method section.

Given the experience of the authors I would encourage them to add a brief paragraph recommending which of the current technologies would they use in other future genome assembly projects, specially when the resources are limited and not all approaches could be carried out.
Response:
Thanks for the reviewer's suggestions. Some technologies in this manuscript have been used in several genome projects, and will be used for more species in the future. Recommending technologies for other genome projects should be a long story. In this manuscript, we think it is better to focus on the improved cucumber genome assembly.

I am also glad to see that the authors have used SRA and ICUGI to make the data available. This is a critical point, the usefulness of a genome assembly depends critically on the data availability, and in this case these are the best two avenues. I haven't been able to find the genome assembly, but I guess that it is not yet public because the manuscript is still under review, but, of course, the sequence should be made public in SRA by the time the manuscript is finally accepted. Also the genome sequence would be much less useful if the annotation is not provided in a easy to use format. So, with the sequence a GFF v3 file with the annotation should be provided.
Response:
The genome sequences and annotations in GFF format are both available in ICUGI under the ftp server: ftp://cucurbitgenomics.org/pub/cucurbit/genome/cucumber/Chinese_long/. The corresponding sequences have been uploaded to SRA and will be made public.

Reviewer #2: This manuscript describes a much improved genome sequence of cucumber assembled from PacBio long reads, 10X linked reads and Hi-C contact maps. Overall the work is well performed and the manuscript is well written. I have made some minor changes and provided some comments directly in the word file of the manuscript, which is attached.
Response:
We thank the reviewer for corrections, and point-to-point responses were added in the revised manuscript.

Reviewer #3: Review of Li et al

The 'Data Note' describes a new assembly of the reference cucumber genome using long read PacBio data and provides a comparison with the previous Illumina based assembly. In general, the note describes the process well and details the main differences between the two assemblies.

As a minor comment, the introduction emphasizes the fact that the current available assemblies have ~150 Mb of missing data, yet this new assembly has only added at most 29 Mb, of which 28 Mb are repeat sequence. So although I agree that the authors have added to the available reference it is not as significant as one would have hoped with long read data.

I would recommend a few clarifications and improvements, which I think would be useful.
Response:
The highly repeated satellite sequences in cucumber, which account for ~30% (~105Mb) of the total nuclear DNA [10, 11], mainly distribute in telomeric, centromeric regions [12]. Given the highly repeated nature of the sequences, PacBio reads are not long enough to encompass these regions, and thus most of the satellite sequences were lost in our assembly. In that case, most boundaries of these genomic segments were assembled, as we can see from the low-contact regions in Hi-C heat map (Main text, Fig. 2). This should be the reason that PacBio reads could not add more extra sequences as we expected. We added some description in the revised version.

It might be interesting to show the actual alignment of the two genomes as a figure.
Response:
We conducted whole-genome alignment between genomes 9930_V3 and 9930_V2 using nucmer program of MUMmer software (version 4.0.0beta2) [4] with parameters "-l 100 -c 100". Based on the alignments, the figure was plotted using python package: svgwrite (https://pypi.org/project/svgwrite/). Poorly-aligned and gap regions mainly reside in centromeres and heterochromatin. A translocation region in chromosome 5 between 9930 V3.0 and 9930 V2.0 was shown, which has been proved to be a mis-assembly in the genome of 9930 V2.0 [13, 14]. Two inversions in chromosome 4 and 6 were validated to be assembly errors in 9930 V2.0 because no obviously abnormal chromatin interaction was identified in these regions as shown in the Hi-C heat map (Main text, Fig. 2). We added the alignment

figure into the supplementary figures (Supplemental Figure S2).

With respect to the assembly can the authors indicate how many contigs had to be split based on genetic data and/or HiC.
Response:
A total of three contigs were split based on the genetic map (Segkk155 Segkk31 Segkk90), and eight (Segkk107 Segkk113 Segkk118 Segkk148 Segkk17 Segkk35 Segkk45 Segkk56) due to the inconsistency with the Hi-C heat map.

For evaluation of the genome quality, how did the authors assess the errors, was this again using Pilon, since this is not described in the materials and methods. Also the authors only mention homozygous errors, what was the rate of heterozygous errors, these could be indicative of problematic assembly regions.
Response：
We used Pilon to polish the genome assembly. To evaluate the genome accuracy, we aligned previous Sanger and 6.0 Gb new Illumina reads onto the contigs using BWA [15]. Based on the alignments, genomic variations were called using GenomeAnalysisTK (http://www.broadinstitute.org/gatk) with default parameters. Considering that the sequenced cucumber 9930 is a high inbreed line, we would expect an extremely low heterozygous rate. And thus only homozygous variations were considered as errors. The heterozygous variations may derive from the incorrect alignments, or sequencing errors, or assembly errors. In fact, only 33 SNPs were identified as heterozygous by filtering the SNP index: 0.3<=SNP index<=0.7.

When looking at the gene annotation, the authors mention 2,693 newly predicted genes that were newly predicted compared to V2, but were present in V3. What were the error types that caused these genes not to be annotated in V2?
Response：
These can be classified into two categories. (1) The whole or partial sequences in V2.0 are absent because of limit of the Illumina sequencing technology. The incorrected or uncompleted assembly may destroy the gene structure and effect the prediction of genes. (2) More RNA-seq data and known protein sequences from uniport (Supplemental Table S4) were used, and some genes with new evidence were predicted.

Also in the materials and methods it is mentioned that there were genes annotated in V2 that were not annotated in V3 but were added to the V3 annotation. How many genes fell into this category and was there an explanation for this - errors in the V3 assembly?
Response：
We updated the annotation pipeline for predicting gene models in genome V3.0. A total of 2,617 genes in V2.0 were not predicted in V3.0. Their genomic sequences in V3.0 are identical with those in V2.0, and thus these should not be derived from the assembly errors. We are also confusing about the results. Aligning these genes against Uniprot protein database found that 84.5% can’t find the homologs. We speculate that these may be due to the different sensitivity of updated pipeline. To facilitating the community researchers to find the corresponding genes of V3.0 and V2.0, we still added these genes into the final gene models.

In general, the figure legends need improving (see below) but for the TPST gene figure the actual gene should be indicated in the figure. I am assuming it is b'? It does seem slightly odd that there is sequence data present in V2 at this point but it is not homologous with V3 and there is no apparent gap in the V2 assembly at this point - are the author sure that this is not an assembly error? Can they see single long reads that encompass multiple genes?
Response：
The figure legends have been revised. According to the genomic synteny between V2.0 and V3.0, the other three TPST genes are located in the gaps in V2.0. To further confirm the multiple copies of TPST, we aligned the PacBio long reads onto the assembled genome using minimap2 [17] with parameters “-ax map-pb”, and show these result in Response Fig.3 using python package: svgwrite (https://pypi.org/project/svgwrite/). As a result, we found high coverage of PacBio reads that encompass multiple genes.

I am not sure the addition of methylation sites is meaningful since it basically reflects the increase in repeats.

Response：
Thanks for your comments. We remove this in the revised manuscript.

Figure 1 legend: Need to explain the different tracks, what are the colours and numbers indicating in the cytogenetic map? What are the orange and red marks? Etc.

Figure 3 legend: Unknown instead of Unknow. In c) what are the acronyms - TSR, PBS, PPT etc. In d) Which is the TPST gene?

Figure 5 legend: Not sure this figure is particularly informative, but what does the y-axis mean - it indicates Lengths (Mb) but in the text it is stated that the value is number of methylation sites.
Response:
We modified the manuscript according to the reviewer's suggestions.

Reviewer #4: Review of the manuscript titled "A chromosome-scale genome assembly of cucumber (Cucumis sativus L.)".
In this manuscript the authors presented a new PacBio chromosome scale genome assembly of the cucumber genome (called V3). The study employed data from different sequencing technologies (Sanger sequencing, PacBio, 10X, Hi-C and Illumina) for de-novo assembly, scaffolding, polishing and gene prediction analysis. Different genetic linkage maps were used for the anchoring and orienting the genome into chromosomes.
The manuscript offers a valuable resource including a high-quality chromosome scale reference genome, a new gene prediction and annotation. However, there are some points that should be appropriately addressed before the publication. See below.

Major: (In general, it would have been helpful if page number were added to the manuscript)
1. Will require some English revisions. Some clear examples: P5 L10-17. "has been served", this sentence in too long. P5 L28-36, sentence too long. P5 L40 "the applications" should be the application. There are more cases throughout the manuscript. I suggest to have a professional reviewer to edit the manuscript.
Response:
We modified the manuscript according to the reviewer's suggestions.

2. The PacBio Sequel raw data are not deposited into NCBI SRA. All the other data PacBio RSII, Hi-C, 10X data are deposited and available.
Response:
The PacBio data are available in NCBI SRA under accession SRP139269 (SRX5437838 for PacBio RSII and SRX5437837 for PacBio Sequel).

3. Missing references: in some parts of the manuscript, would be better to add reference(s):
a. P7, L1: In cucumber, the repetitive sequences are estimated to account for 30%, (please add a ref)
b. P9, L27: Two predicted TPSTs in the wild cucumber genome also support multiple TPSTs in the cucumber genome (please add a ref)
c. P11, L2: NCBI's Short Read Archive (please add a ref or a link)
Response:
We modified the manuscript according to the reviewer's suggestions.

4. Although authors conducted some analysis for identification of novel predicted genes in the newly assembled genome v3 comparing to the v2 genome assembly, but no comparative analysis checking the improved gene models in v3 vs v2 (checking for fragmented gene models, split genes models etc.) is conducted. I would suggest to add this analysis in the manuscript, the improved genome could have improved the structure of predicted genes especially with the use of IsoSeq data.
Response：
Based on the alignments of genome V3.0 and V2.0, we identified 1,970 fragmented genes in V2.0 which correspond to 932 genes in V3.0. Conversely, 687 genes in V2.0 are split into 337 in V3.0. These were added in the revised manuscript (Supplemental Figure S5).
In the future, we will further improve the gene models using IsoSeq data.

5. Although authors report that 239 out of 1,078 novel genes were tandemly duplicated in V3.0 genome "Distribution analysis of the 1,078 novel genes in V3.0 along the pseudo-chromosomes showed that 239 are tandemly duplicated genes", however no description about how these analysis were conducted is

presented in the manuscript.
Response：
To identify the tandemly duplicated genes in these novel genes, we detected all tandemly duplicated genes (TDGs) in V3.0 genome. Next, OrthoMCL [20] was used to identify orthologous groups in V3.0 genes. If the genes are in the same orthologous group and located next to each other on one chromosome, they are considered as TDGs. These were added into the method section.

6. Authors reported that four copies of TPST genes were predicted and identified in the v3 genome while this gene is in single copy in most other plants, proposing that this gene is responsible for some specific traits in cucurbits. In another place authors also report sequencing gaps around the flanking regions of this gene in melon and watermelons, but in none of the above cases no further information is reported. I believe that at least the ID of these genes, their chromosomal position/distribution, and some plots demonstrating what is presented should be presented.
Response：
Thanks for your suggestion. We added the information of TPST genes, include the gene ID, chromosomal position and function description as supplemental file (Supplemental Table S6).

7. Please also include the L50 number in Supplemental table S4
Response:
We have added the L50 number in Supplemental table S3. The L50 number of Scaffold and Super-scaffold are 8 and 4, respectively (Supplemental table S3).

8.In the introduction the authors indicated that about 150Mb assembly are missing in the V2 assembly. The authors should comment and could explore the PacBio data for this missing portion of the genome.
Response:
The highly repeated satellite sequences in cucumber, which account for ~30% (~105Mb) of the total nuclear DNA [10, 11], mainly distribute in telomeric, centromeric regions [12]. Given the highly repeated nature of the sequences, PacBio reads are not long enough to encompass these regions, and thus most of the satellite sequences were lost in our assembly. In that case, most boundaries of these genomic segments were assembled, as we can see from the low-contact regions in Hi-C heat map (Main text, Fig. 2). This should be the reason that PacBio reads could not add more extra sequences as we expected. We added some description in the revised version.

Minor:
1. Legends in some of the plots are not clear
a. fig1: please specify that numbers (eg. 1-1) correspond to BAC-clone?
b. The font by the color legend (eg. the repeat type) is too small
c. I could not also find the Fosmid tracks in fig1.
d. Please separate the Hic Plot and Linkage map plots by a. and b. and reflect this change in the figure legend.
2. P5, L1: "chromosome scale assembly" => "chromosome scale genome assembly"
3. P6, L30: "additional 19.1 Mb sequences": please specify that is comparing to the v2 genome assembly…
4. P6, L32: "lacking contact information" => "lacking Hi-C contact information"
5. P7, L21: the sentence is confusing. Please rephrase (e.g. using such technologies, the contiguity of the cucumber genome assembly can be significantly increased)
6. P7, L36: "genetics": you mean "genetic studies"?
7. P8, L30: SRA ID is missing
8. P8, L51: "markedly" => "remarkably" or "significantly"
9. P8, L60: "Dating"? please rephrase, the message is not clear
10. P9 L10-14:
a. Move the "(Supplemental table S7)" to the end of the paragraph
Response:
We modified the manuscript according to the reviewer’s suggestions.

b. if you are listing those newly assembled, please also list those newly predicted with the coordinates and structure if possible
Response：
We added the information of these genes, include the gene ID, chromosomal position and function description as supplemental file (Supplemental Table S6).

c. a simple statistic for the newly predicted genes (avg. ln, average # of exons etc.) wuold be useful
Response：
We compared the average length and number of exons and intron of genes between the whole set of genes and newly predicted ones. The average number of exons per gene in whole and newly genes are five and four, respectively. They were added into the revised manuscript (Supplemental Figure S4).

11. P9, L34: "contact" is not clear
12. P9, L43-45: cannot be mapped? What do you mean by this? The use of Illumina reads here is confusing and not clear. Authors might want to rephrase the sentence.
13. P11, L2: "NCBI's Small Read Archive" => "NCBI Short Read Archive"
14. P11, L57-58: "(See supplemental table S3 for detailed information)", Please follow the same format when referring to the supplemental information
15. Please also include the L50 number in Supplemental table S4
Response:
We modified the manuscript according to the reviewer’s suggestions.

Reference
1. Koren S, Walenz BP, Berlin K, Miller JR, Bergman NH and Phillippy AM. Canu: scalable and accurate long-read assembly via adaptive k-mer weighting and repeat separation. Genome Res. 2017;27 5:722-36. doi:10.1101/gr.215087.116.
2. Lam KK, LaButti K, Khalak A and Tse D. FinisherSC: a repeat-aware tool for upgrading de novo assembly using long reads. Bioinformatics. 2015;31:3207-9.
3. Walker BJ, Abeel T, Shea T, Priest M, Abouelliel A, Sakthikumar S, et al. Pilon: an integrated tool for comprehensive microbial variant detection and genome assembly improvement. PLoS One. 2014;9:e112963.
4. Kurtz S, Phillippy A, Delcher AL, Smoot M, Shumway M, Antonescu C, et al. Versatile and open software for comparing large genomes. Genome Biol. 2004;5:R12.
5. Yang LM, Koo DH, Li YH, Zhang XJ, Luan FS, Havey MJ, et al. Chromosome rearrangements during domestication of cucumber as revealed by high-density genetic mapping and draft genome assembly. Plant J. 2012;71:895-906.
6. Ren Y, Zhang Z, Liu J, Staub JE, Han Y, Cheng Z, et al. An integrated genetic and cytogenetic map of the cucumber genome. PLoS One. 2009;4:e5795.
7. Zhang WW, Pan JS, He HL, Zhang C, Li Z, Zhao JL, et al. Construction of a high density integrated genetic map for cucumber (Cucumis sativus L.). Theor Appl Genet. 2012;124:249-59.
8. Zhou Q, Miao H, Li S, Zhang S, Wang Y, Weng Y, et al. A Sequencing-Based Linkage Map of Cucumber. Mol Plant. 2015;8:961-3.
9. Tang H, Zhang X, Miao C, Zhang J, Ming R, Schnable JC, et al. ALLMAPS: robust scaffold ordering based on multiple maps. Genome Biol. 2015;16:3. doi:10.1186/s13059-014-0573-1.
10. GanalI M and Hemleben R. Organization and sequence analysis of two related satellite DNAs in cucumber (Cucumis sativus L.). Journal of Molecular Evolution. 1986;23 1:23-30.
11. GanalI M and Hemleben R. Insertion and amplification of a DNA sequence in satellite DNA of Cucumis sativus L. (cucumber). Theoretical and Applied Genetics. 1988;75 2:357-61.
12. Han YH, Zhang ZH, Liu JH, Lu JY, Huang SW and Jin WW. Distribution of the tandem repeat sequences and karyotyping in cucumber (Cucumis sativus L.) by fluorescence in situ hybridization. Cytogenet Genome Res. 2008;122 1:80-8. doi:10.1159/000151320.
13. Zhou Q, Miao H, Li S, Zhang S, Wang Y, Weng Y, et al. A sequencing-based linkage map of cucumber. Molecular plant. 2015;8 6:961-3.
14. Sun JY, Zhang ZH, Zong X, Huang SW, Li ZY and Han YH. A high-resolution cucumber cytogenetic map integrated with the genome assembly. Bmc Genomics. 2013;14 doi:Artn 46110.1186/1471-2164-14-461.
15. Li H and Durbin R. Fast and accurate short read alignment with Burrows-Wheeler transform. Bioinformatics. 2009;25:1754-60.
16. Gotoh O. A space-efficient and accurate method for mapping and aligning cDNA sequences onto genomic sequence. Nucleic Acids Res. 2008;36:2630-8.
17. Li H. Minimap2: pairwise alignment for nucleotide sequences. Bioinformatics. 2018;34 18:3094-100. doi:10.1093/bioinformatics/bty191.
18. Li H and Durbin R. Fast and accurate short read alignment with Burrows-Wheeler transform. Bioinformatics. 2009;25 14:1754-60. doi:10.1093/bioinformatics/btp324.
19. Thorvaldsdottir H, Robinson JT and Mesirov JP. Integrative Genomics Viewer (IGV): high-performance genomics data visualization and exploration. Brief Bioinform. 2013;14 2:178-92.

doi:10.1093/bib/bbs017.
20. Li L, Stoeckert CJ, Jr. and Roos DS. OrthoMCL: identification of ortholog groups for eukaryotic genomes. Genome Res. 2003;13:2178-89.

Close
