# Supplementary material for: A chromosome-scale genome assembly of cucumber (Cucumis sativus L.)
Source: Gigascience. 2019 Jun 18;8(6):giz072. doi: 10.1093/gigascience/giz072 (PMC6582320; doi:10.1093/gigascience/giz072)
Supplement: giz072_Response_to_Reviewer_Comments_Revision_1 [file giz072_response_to_reviewer_comments_revision_1.pdf]

Author's Response To Reviewer Comments

Close

We greatly appreciate the dedication of the reviewers and the editor to help us to improve the manuscript. According to the reviewer and editor’s comments and suggestions, we have revised our manuscript and upload them again.

Close
