# Supplementary material for: A chromosome-scale genome assembly of cucumber (Cucumis sativus L.)
Source: Gigascience. 2019 Jun 18;8(6):giz072. doi: 10.1093/gigascience/giz072 (PMC6582320; doi:10.1093/gigascience/giz072)
Supplement: giz072_Reviewer_1_Report_Original_Submission — JosÃ© Blanca -- 1/24/2019 Reviewed [file giz072_reviewer_1_report_original_submission.pdf]

Reviewer Report

Title: A chromosome-scale genome assembly of cucumber (Cucumis sativus L.)

Version: Original Submission **Date:** 1/24/2019

Reviewer name: JosÃ© Blanca

Reviewer Comments to Author:

24-1-2019
The authors have kindly offered access to the genome sequence and they also have explained that the genome sequence will be available in SRA by the time the paper is published. This completely solves the issue regarding the genome sequence. However, I still have doubts regarding the annotation. Will be the annotation made available in the genebank along with the sequence? As a GFF file in ICUGI? With a GFF file attached to the manuscript as supplementary material? Any of those options would be fine, because they will ease the use of the genome sequence by the community. I hope this issue is clarified soon and so the community can enjoy the great advance presented by the authors. I have no doubts that this paper will be a methodological reference.

21-1-2019
The reported V3.0 of the cucumber genome is not just a very useful tool for the cucumber community, but a great standard to follow in future genome assembly projects.
The methods used are appropriate and the results just great.
The manuscript is well written and clear. I have just detected a minor mistake, the authors have misspell telomeric as telemeric. (Although there could be more mistakes, I am not a native English speaker).
Are the methods appropriate to the aims of the study, are they well described, and are necessary controls included?
Given the experience of the authors I would encourage them to add a brief paragraph recommending which of the current technologies would they use in other future genome assembly projects, specially when the resources are limited and not all approaches could be carried out.
I am also glad to see that the authors have used SRA and ICUGI to make the data available. This is a critical point, the usefulness of a genome assembly depends critically on the data availability, and in this case these are the best two avenues. I haven't been able to find the genome assembly, but I guess that it is not yet public because the manuscript is still under review, but, of course, the sequence should be made public in SRA by the time the manuscript is finally accepted. Also the genome sequence would be much less useful if the annotation is not provided in a easy to use format. So, with the sequence a GFFv3 file with the annotation should be provided.

Level of Interest

Please indicate how interesting you found the manuscript: Choose an item.

Quality of Written English

Please indicate the quality of language in the manuscript: Choose an item.

Declaration of Competing Interests

Please complete a declaration of competing interests, considering the following questions:

- Have you in the past five years received reimbursements, fees, funding, or salary from an organisation that may in any way gain or lose financially from the publication of this manuscript, either now or in the future?
- Do you hold any stocks or shares in an organisation that may in any way gain or lose financially from the publication of this manuscript, either now or in the future?
- Do you hold or are you currently applying for any patents relating to the content of the manuscript?
- Have you received reimbursements, fees, funding, or salary from an organization that holds or has applied for patents relating to the content of the manuscript?
- Do you have any other financial competing interests?
- Do you have any non-financial competing interests in relation to this paper?

If you can answer no to all of the above, write 'I declare that I have no competing interests' below. If your reply is yes to any, please give details below.

I declare that I have no competing interests

I agree to the open peer review policy of the journal. I understand that my name will be included on my report to the authors and, if the manuscript is accepted for publication, my named report including any attachments I upload will be posted on the website along with the authors' responses. I agree for my report to be made available under an Open Access Creative Commons CC-BY license (http://creativecommons.org/licenses/by/4.0/). I understand that any comments which I do not wish to be included in my named report can be included as confidential comments to the editors, which will not be published.

I agree to the open peer review policy of the journal

To further support our reviewers, we have joined with Publons, where you can gain additional credit to further highlight your hard work (see: https://publons.com/journal/530/gigascience). On publication of this paper, your review will be automatically added to Publons, you can then choose whether or not to claim your Publons credit. I understand this statement.

Yes Choose an item.
