# Supplementary material for: A chromosome-scale genome assembly of cucumber (Cucumis sativus L.)
Source: Gigascience. 2019 Jun 18;8(6):giz072. doi: 10.1093/gigascience/giz072 (PMC6582320; doi:10.1093/gigascience/giz072)
Supplement: giz072_Reviewer_3_Report_Original_Submission — Isobel Parkin -- 2/7/2019 Reviewed [file giz072_reviewer_3_report_original_submission.pdf]

Reviewer Report

Title: A chromosome-scale genome assembly of cucumber (Cucumis sativus L.)

Version: Original Submission Date: 2/7/2019

Reviewer name: Isobel Parkin

Reviewer Comments to Author:

Review of Li et al
The 'Data Note' describes a new assembly of the reference cucumber genome using long read PacBio data and provides a comparison with the previous Illumina based assembly. In general the note describes the process well and details the main differences between the two assemblies.
As a minor comment, the introduction emphasizes the fact that the current available assemblies have ~150 Mb of missing data, yet this new assembly has only added at most 29 Mb, of which 28 Mb are repeat sequence. So although I agree that the authors have added to the available reference it is not as significant as one would have hoped with long read data.
I would recommend a few clarifications and improvements, which I think would be useful.
It might be interesting to show the actual alignment of the two genomes as a figure.
With respect to the assembly can the authors indicate how many contigs had to be split based on genetic data and/or HiC.
For evaluation of the genome quality, how did the authors assess the errors, was this again using Pilon, since this is not described in the materials and methods. Also the authors only mention homozygous errors, what was the rate of heterozygous errors, these could be indicative of problematic assembly regions.
When looking at the gene annotation, the authors mention 2,693 newly predicted genes that were newly predicted compared to V2, but were present in V3. What were the error types that caused these genes not to be annotated in V2?
Also in the materials and methods it is mentioned that there were genes annotated in V2 that were not annotated in V3 but were added to the V3 annotation. How many genes fell into this category and was there an explanation for this - errors in the V3 assembly?
In general the figure legends need improving (see below) but for the TPST gene figure the actual gene should be indicated in the figure. I am assuming it is b'? It does seem slightly odd that there is sequence data present in V2 at this point but it is not homologous with V3 and there is no apparent gap in the V2 assembly at this point - are the author sure that this is not an assembly error? Can they see single long reads that encompass multiple genes?
I am not sure the addition of methylation sites is meaningful since it basically reflects the increase in repeats.
Figure 1 legend: Need to explain the different tracks, what are the colours and numbers indicating in the cytogenetic map? What are the orange and red marks? Etc..
Figure 3 legend: Unknown instead of Unknow. In c) what are the acronyms - TSR, PBS, PPT etc. In d) Which is the TPST gene?

Figure 5 legend: Not sure this figure is particularly informative, but what does the y-axis mean - it indicates Lengths(Mb) but in the text it is stated that the value is number of methylation sites.

Level of Interest

Please indicate how interesting you found the manuscript: Choose an item.

Quality of Written English

Please indicate the quality of language in the manuscript: Choose an item.

Declaration of Competing Interests

Please complete a declaration of competing interests, considering the following questions:

- Have you in the past five years received reimbursements, fees, funding, or salary from an organisation that may in any way gain or lose financially from the publication of this manuscript, either now or in the future?
- Do you hold any stocks or shares in an organisation that may in any way gain or lose financially from the publication of this manuscript, either now or in the future?
- Do you hold or are you currently applying for any patents relating to the content of the manuscript?
- Have you received reimbursements, fees, funding, or salary from an organization that holds or has applied for patents relating to the content of the manuscript?
- Do you have any other financial competing interests?
- Do you have any non-financial competing interests in relation to this paper?

If you can answer no to all of the above, write 'I declare that I have no competing interests' below. If your reply is yes to any, please give details below.

I declare that I have no competing interests.

I agree to the open peer review policy of the journal. I understand that my name will be included on my report to the authors and, if the manuscript is accepted for publication, my named report including any attachments I upload will be posted on the website along with the authors' responses. I agree for my report to be made available under an Open Access Creative Commons CC-BY license (http://creativecommons.org/licenses/by/4.0/). I understand that any comments which I do not wish to be included in my named report can be included as confidential comments to the editors, which will not be published.

I agree to the open peer review policy of the journal

To further support our reviewers, we have joined with Publons, where you can gain additional credit to further highlight your hard work (see: https://publons.com/journal/530/gigascience). On publication of this paper, your review will be automatically added to Publons, you can then choose whether or not to claim your Publons credit. I understand this statement.

Yes Choose an item.
