# Supplementary material for: A chromosome-scale genome assembly of cucumber (Cucumis sativus L.)
Source: Gigascience. 2019 Jun 18;8(6):giz072. doi: 10.1093/gigascience/giz072 (PMC6582320; doi:10.1093/gigascience/giz072)
Supplement: giz072_Reviewer_4_Report_Original_Submission — Massimo Iorizzo -- 2/19/2019 Reviewed [file giz072_reviewer_4_report_original_submission.pdf]

Reviewer Report

Title: A chromosome-scale genome assembly of cucumber (Cucumis sativus L.)

Version: Original Submission **Date:** 2/19/2019

Reviewer name: Massimo Iorizzo

Reviewer Comments to Author:

Review of the manuscript titled "A chromosome-scale genome assembly of cucumber (Cucumis sativus L.)".

In this manuscript the authors presented a new PacBio chromosome scale genome assembly of the cucumber genome (called V3). The study employed data from different sequencing technologies (Sanger sequencing, PacBio, 10X, Hi-C and Illumina) for de-novo assembly, scaffolding, polishing and gene prediction analysis. Different genetic linkage maps were used for the anchoring and orienting the genome into chromosomes.

The manuscript offers a valuable resource including a high-quality chromosome scale reference genome, a new gene prediction and annotation. However, there are some points that should be appropriately addressed before the publication. See below.

Major: (In general, it would have been helpful if page number were added to the manuscript)

1. Will require some English revisions. Some clear examples: P5 L10-17. "has been served", this sentence in too long. P5 L28-36, sentence too long. P5 L40 "the applications" should be the application. There are more cases throughout the manuscript. I suggest to have a professional reviewer to edit the manuscript.
2. The PacBio Sequel raw data are not deposited into NCBI SRA. All the other data PacBio RSII, Hi-C, 10X data are deposited and available.
3. Missing references: in some parts of the manuscript, would be better to add reference(s):
a. P7, L1: In cucumber, the repetitive sequences are estimated to account for 30%, (please add a ref)
b. P9, L27: Two predicted TPSTs in the wild cucumber genome also support multiple TPSTs in the cucumber genome (please add a ref)
c. P11, L2: NCBI's Short Read Archive (please add a ref or a link)
4. Although authors conducted some analysis for identification of novel predicted genes in the newly assembled genome v3 comparing to the v2 genome assembly, but no comparative analysis checking the improved gene models in v3 vs v2 (checking for fragmented gene models, split genes models etc.) is conducted. I would suggest to add this analysis in the manuscript, the improved genome could have improved the structure of predicted genes especially with the use of IsoSeq data.
5. Although authors report that 239 out of 1,078 novel genes were tandemly duplicated in V3.0 genome "Distribution analysis of the 1,078 novel genes in V3.0 along the pseudo-chromosomes showed that 239 are tandemly duplicated genes", however no description about how these analysis were conducted is presented in the manuscript.
6. Authors reported that four copies of TPST genes were predicted and identified in the v3 genome while this gene is in single copy in most other plants, proposing that this gene is responsible for some specific traits in cucurbits. In another place authors also report sequencing gaps around the flanking

regions of this gene in melon and watermelons, but in none of the above cases no further information is reported. I believe that at least the ID of these genes, their chromosomal position/distribution, and some plots demonstrating what is presented should be presented.
7. Please also include the L50 number in Supplemental table S4
8. In the introduction the authors indicated that about 150Mb assembly are missing in the V2 assembly. The authors should comment and could explore the PacBio data for this missing portion of the genome.
Minor:
1. Legends in some of the plots are not clear
a. fig1: please specify that numbers (eg. 1-1) correspond to BAC-clone?
b. The font by the color legend (eg. the repeat type) is too small
c. I could not also find the Fosmid tracks in fig1.
d. Please separate the Hic Plot and Linkage map plots by a. and b. and reflect this change in the figure legend.
2. P5, L1: "chromosome scale assembly" => "chromosome scale genome assembly"
3. P6, L30: "additional 19.1 Mb sequences": please specify that is comparing to the v2 genome assembly…
4. P6, L32: "lacking contact information" => "lacking Hi-C contact information"
5. P7, L21: the sentence is confusing. Please rephrase (e.g. using such technologies, the contiguity of the cucumber genome assembly can be significantly increased)
6. P7, L36: "genetics": you mean "genetic studies"?
7. P8, L30: SRA ID is missing
8. P8, L51: "markedly" => "remarkably" or "significantly"
9. P8, L60: "Dating"? please rephrase, the message is not clear
10. P9 L10-14:
a. Move the "(Supplemental table S7)" to the end of the paragraph
b. if you are listing those newly assembled, please also list those newly predicted with the coordinates and structure if possible
c. a simple statistic for the newly predicted genes (avg. ln, average # of exons etc.) wuold be useful
11. P9, L34: "contact" is not clear
12. P9, L43-45: cannot be mapped? What do you mean by this? The use of Illumina reads here is confusing and not clear. Authors might want to rephrase the sentence.
13. P11, L2: "NCBI's Small Read Archive" => "NCBI Short Read Archive"
14. P11, L57-58: "(See supplemental table S3 for detailed information)", Please follow the same format when referring to the supplemental information
15. Please also include the L50 number in Supplemental table S4

Level of Interest

Please indicate how interesting you found the manuscript: Choose an item.

Quality of Written English

Please indicate the quality of language in the manuscript: Choose an item.

Declaration of Competing Interests

Please complete a declaration of competing interests, considering the following questions:

- Have you in the past five years received reimbursements, fees, funding, or salary from an organisation that may in any way gain or lose financially from the publication of this manuscript, either now or in the future?
- Do you hold any stocks or shares in an organisation that may in any way gain or lose financially from the publication of this manuscript, either now or in the future?
- Do you hold or are you currently applying for any patents relating to the content of the manuscript?
- Have you received reimbursements, fees, funding, or salary from an organization that holds or has applied for patents relating to the content of the manuscript?
- Do you have any other financial competing interests?
- Do you have any non-financial competing interests in relation to this paper?

If you can answer no to all of the above, write 'I declare that I have no competing interests' below. If your reply is yes to any, please give details below.

I declare that I have no competing interests

I agree to the open peer review policy of the journal. I understand that my name will be included on my report to the authors and, if the manuscript is accepted for publication, my named report including any attachments I upload will be posted on the website along with the authors' responses. I agree for my report to be made available under an Open Access Creative Commons CC-BY license (http://creativecommons.org/licenses/by/4.0/). I understand that any comments which I do not wish to be included in my named report can be included as confidential comments to the editors, which will not be published.

I agree to the open peer review policy of the journal

To further support our reviewers, we have joined with Publons, where you can gain additional credit to further highlight your hard work (see: https://publons.com/journal/530/gigascience). On publication of this paper, your review will be automatically added to Publons, you can then choose whether or not to claim your Publons credit. I understand this statement.

Yes Choose an item.
